# The photochemical alkylation and reduction of heteroarenes†
†Electronic supplementary information (ESI) available. See DOI: 10.1039/c7sc03768f



**DOI:** 10.1039/c7sc03768f

**Published:** 2017-09-11

**Authors:** T. McCallum, S. P. Pitre, M. Morin, J. C. Scaiano, L. Barriault

**Affiliations:** a Centre for Catalysis , Research and Innovation , Department of Chemistry and Biomolecular Sciences , University of Ottawa , 10 Marie Curie , Ottawa , ON K1N 6N5 , Canada . Email: jscaiano@uottawa.ca ; Email: lbarriau@uottawa.ca

## Abstract

The functionalization of heteroarenes has been integral to the structural diversification of medicinally active molecules such as quinolines, pyridines, and phenanthridines. Electron-deficient heteroarenes are electronically compatible to react with relatively nucleophilic free radicals such as hydroxyalkyl. However, the radical functionalization of such heteroarenes has been marked by the use of transition-metal catalyzed processes that require initiators and stoichiometric oxidants. Herein, we describe the photochemical alkylation of quinolines, pyridines and phenanthridines, where through direct excitation of the protonated heterocycle, alcohols and ethers, such as methanol and THF, can serve as alkylating agents. We also report the discovery of a photochemical reduction of these heteroarenes using only iPrOH and HCl. Mechanistic studies to elucidate the underlying mechanism of these transformations, and preliminary results on catalytic methylations are also reported.

## Abstract 2

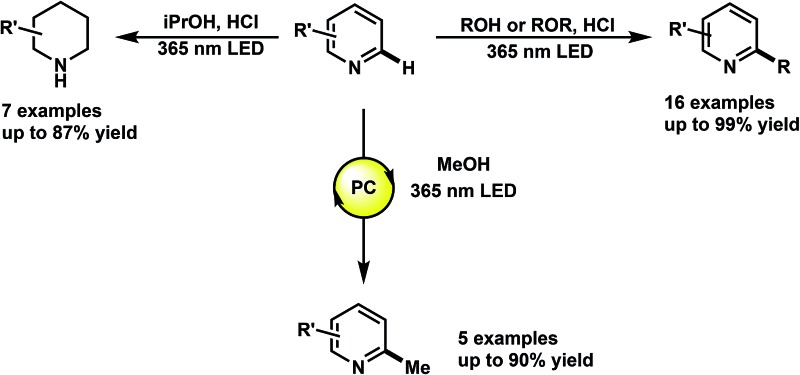
The functionalization of heteroarenes has been integral to the structural diversification of medicinally active molecules such as quinolines, pyridines, and phenanthridines.

## Introduction

Over the past decade, the rapidly increasing development of photochemical based organic transformations has been driven by research in photoredox catalysis. Transition-metal photocatalysts and organic dyes have granted chemists access to ever increasing alternatives to classical radical chemistry transformations as well as unconventional reactivity in the discovery of new organic transformations.^1^ Implementation of photochemical processes is preferential to the classical, thermal methods of radical generation, as they often eliminate the need for radical initiators, stoichiometric additives and harsh reaction conditions.

**Fig. 1:**
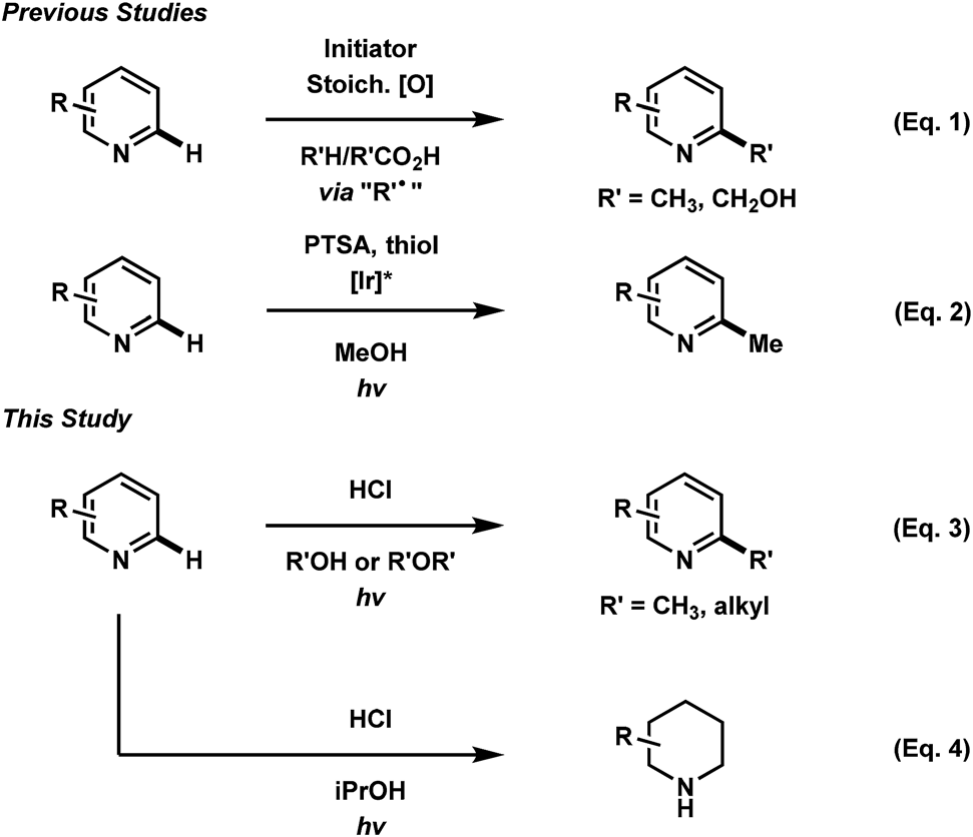
Previous and present work in direct heteroarene alkylation.

The functionalization of heteroaromatic scaffolds is an important topic in organic chemistry for its applications in the synthesis of biologically active molecules and other medicinally relevant studies such as structure–activity relationships.^2^ The Minisci reaction has been famously important in the alkyl/aryl functionalization of electron-deficient heteroaromatic scaffolds.^3^ In this regard, a relatively nucleophilic radical (alkyl or aryl) is generated by an initiation process, where upon addition to an electron-deficient heteroaromatic (usually protonated), can undergo rearomatization by an oxidation event, leading to the functionalized product. In the original report,^3^ the silver mediated decarboxylation or carboxylic acids in presence of persulfates was described, however this reaction suffers from byproduct formation, low yields in some cases and the need for relatively harsh conditions (Fig. 1, eqn (1)). Since then, a variety of mild methodological advancements have been made using many functional groups.^4^ One such functionalization that has been particularly challenging to organic chemists is the methylation of heteroaromatic scaffolds. Only a few striking protocols exist for this ambitious disconnection, demonstrating the need for further methodological development (eqn (2)).^5^ The use of methanol as a precursor for methyl functionality also presents a significant challenge in organic synthesis as few methodologies have been developed (mostly with transition-metals) for this difficult disconnection.^6^ In light of these reports, a mild and waste-limiting organic-based protocol employing MeOH would provide ideal methylation conditions.^7^ Herein, we report the photochemical activation of protonated heteroarenes for their methylation in methanol (eqn (3)) as well as studies with a variety of other alcohols and ethers. The use of catalytic quantities of 2,4-diphenylquinoline for the methylation of heteroarenes that do not absorb in the UVA region or that degrade upon direct excitation is also disclosed. Additionally, we also report the discovery of an iPrOH mediated reduction of heteroarenes (eqn (4)), which to the best of our knowledge is the first organic mediated photochemical protocol for the reduction of heteroarenes. Finally, mechanistic studies were performed to aid in the elucidation of the overall reaction mechanism is also disclosed.

## Results and discussion

Over the course of our previous study on the photoredox catalyzed alkylative functionalization of heteroarenes such as lepidine (**1a**) using haloalkanes in methanol,^4*i*^ methanesulfonyl chloride was found to afford 2,4-dimethylquinoline (**2a**) in absence of a photoredox catalyst. Furthermore, the use of butyl- or tosyl-functionalized sulfonyl chlorides also gave rise to the methylated product rather than the corresponding sulfonyl alkyl/aryl functionalities that may have derived from the addition or fragmentation of RSO_2_ radicals. Subsequently, it was found that the methylation proceeded to give **2a** (76% yield) in the presence of concentrated hydrochloric acid (*c* = 2.0 M) under UVA LED irradiation (Table 1, entry 1). On one hand, a screening of various co-solvents was performed to test the viability of extending this methodology to a variety of alkanols. It was found that MeCN can be a good co-solvent, yielding **2a** in 77% (entry 5). On the other hand, the optimization of HCl equivalents and reaction concentration gave **2a** in 80% yield using HCl (5 equiv.) in MeOH (0.5 M) (entry 11). Interestingly, the byproduct **4a** was isolated in 40% yield after 60 h of UVA irradiation (entry 12). Starting from **1a** or **2a** gave the same product distribution over the 60 h experiment, indicating that the methylation reaction occurs at a faster rate relative to the onset of photochemical degradation of **2a**. The reaction was also amenable to scale up (10 mmol), producing **2a** in 65% yield (entry 13). Control experiments verified that the transformation required acid as well as UVA LED irradiation for product formation (entries 14 and 15). The use of acids such as TFA, H_2_SO_4_, and HOTf gave little to no conversion of the starting material whereas PTSA lead mostly to degradation (entries 16–19). Finally, the reaction conditions were not hampered when exposed to air atmosphere, indicating that the reaction likely proceeds *via* the excited singlet state (entry 20). Notably, exposing the reaction to oxygen atmosphere resulted in the hydroxymethylated product **3a** in 59% yield (56% isolated, entry 21). In our hands, this reaction only converted **1a** to **2a** when using 2 UVA LEDs at ∼1 mm away from the reaction vessel, resulting in the generation of heat (∼70 °C). Using a temperature controlled setup, the optimized conditions gave no conversion after 16 h at 20 °C, showing the need for both light and heat for the successful transformation of **1a**. A survey of the literature reveals that previous photo-mediated methylation reactions were described in low yield (<10%). These examples employed intense sources of irradiation, possibly leading to the degradation of the methylated heteroarene products.^8^ Given the simplicity of this transformation, we surmised an investigation of reaction scope and mechanistic studies would be beneficial to the understanding of our results.

**Table 1:** Optimization of reaction conditions^*a*^

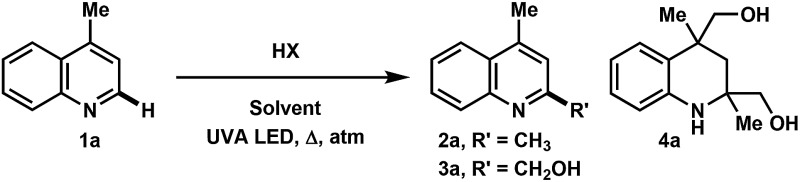										
Entry	HX	HX [equiv.]	Solvent	*M*	atm	*t* [h]	Conv. SM [%]	**2a** [%]	**3a** [%]	**4a** [%]
1	HCl	5	MeOH	0.5	Ar	16	100	76	—	20
2	HCl	5	MeOH : H_2_O (1 : 1)	0.5	Ar	16	39	27	—	—
3	HCl	5	MeOH : DMSO (1 : 1)	0.5	Ar	16	33	16	—	—
4	HCl	5	MeOH : DMF (1 : 1)	0.5	Ar	16	58	29	—	—
5	HCl	5	MeOH : MeCN (1 : 1)	0.5	Ar	16	90	77	—	5
6	HCl	5	MeOH	1.0	Ar	16	76	69	—	5
7	HCl	5	MeOH	0.3	Ar	16	100	51	—	30
8	HCl	5	MeOH	0.1	Ar	16	100	50	—	13
9	HCl	3	MeOH	0.5	Ar	16	97	82	—	7
10	HCl	1	MeOH	0.5	Ar	16	48	27	10	3
**11**	**HCl**	**5**	**MeOH**	**0.5**	**Ar**	**8**	**100**	**87(80)**	**—**	**10**
12	HCl	5	MeOH	0.5	Ar	60	100	35	—	40(40)^*b*^
13	HCl	5	MeOH	0.5	Ar	16	72	65^*c*^	—	5
14	—	—	MeOH	0.5	Ar	16	0	—	—	—
15	HCl	5	MeOH	0.5	Ar	16	0	—^*d*^	—	—
16	TFA	5	MeOH	0.5	Ar	8	10	5	—	—
17	PTSA	5	MeOH	0.5	Ar	8	94	28	12	—
18	H_2_SO_4_	5	MeOH	0.5	Ar	8	12	7	—	—
19	HOTf	5	MeOH	0.5	Ar	8	7	6	—	—
20	HCl	5	MeOH	0.5	Air	8	100	84	—	7
21	HCl	2	MeOH	0.5	O_2_	4	97	38	59(56)	—

^*a*^Procedure: **1a** (0.4 mmol), solvent (0.5 M relative to **1a**), HX (*x* equiv.), Ar or O_2_ degas, irradiation with 2× UVA LEDs (approximately 1 mm from vial, bringing reaction temperature to ∼70 °C). Yields determined by ^1^H NMR analysis reported after basic aqueous work-up (1.0 M NaOH/DCM) using mesitylene as internal standard (isolated yields).

^*b*^Isolated as a 2 : 1 ratio of diastereomers (d.r. determined by ^1^H NMR analysis). The same reaction conditions using **2a** as starting material resulted in the same product distribution.

^*c*^10.0 mmol scale of **1a**.

^*d*^In absence of irradiation and heating to reflux.

In the investigation of the methylation scope under optimized conditions, a variety of heteroarenes were evaluated (Table 2A). Quinaldine and 8-chloroquinaldine gave mostly the corresponding degradation products related to **4a**. Interestingly, 6- and 7-haloquinaldine (X = F, Cl, and Br) gave dimethylquinoline products **2b–e** in 32–68% yields, where the main by-products were found to be similar to **4a** (by-product for X = Cl characterized, see ESI†). 2,6-Diphenylpyridine, fluorinated phenylpyridine and 2-phenylpyridine afforded methylated products **2f**, **2g** and **2h** in 82%, 61% and 59% yields, respectively. Using 410 nm LEDs for 2-phenylquinoline and phenanthridine, methylation was obtained in nearly quantitative yield.

**Table 2:** Scope of heteroarene alkylation and reduction^*a*^

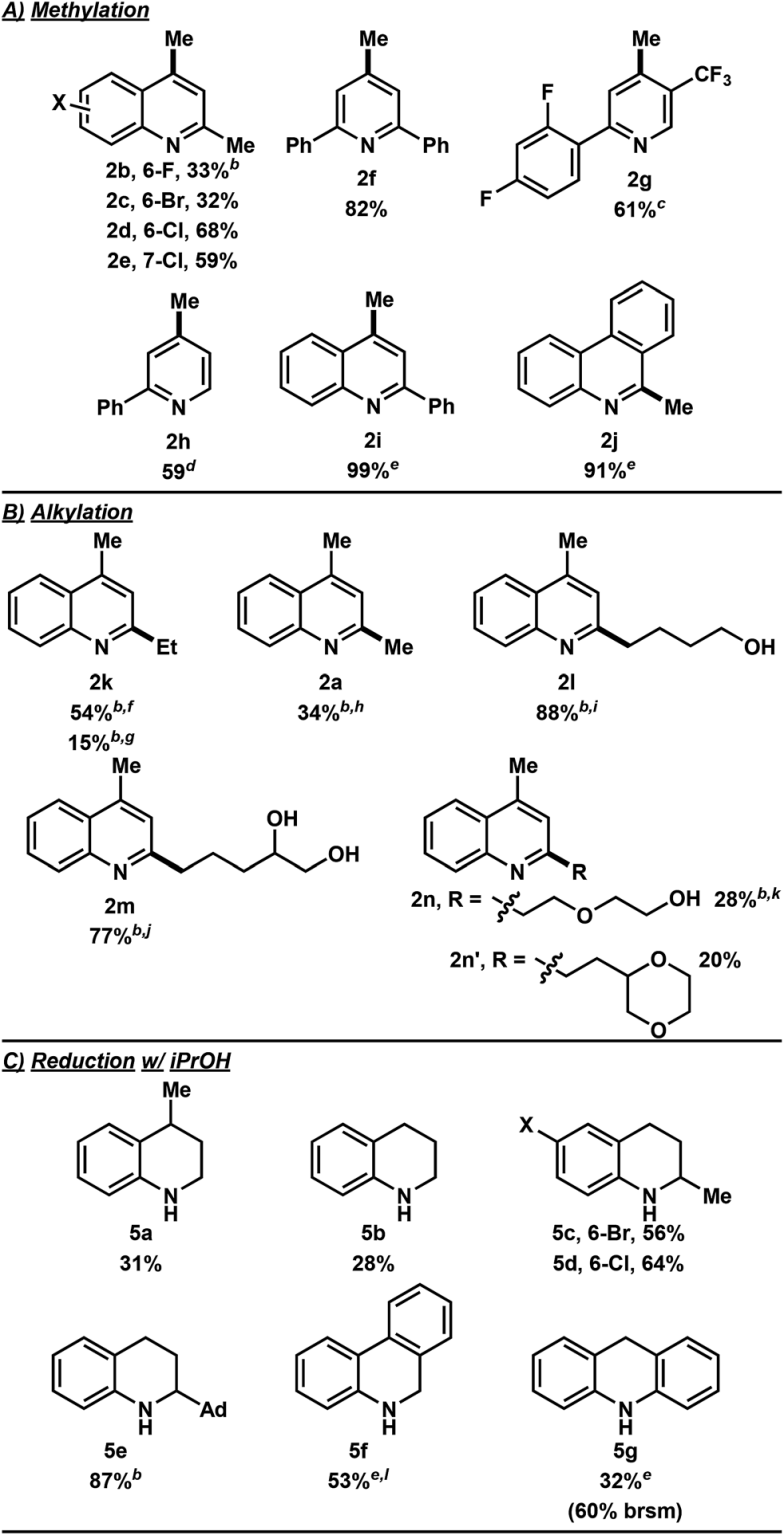

^*a*^Procedure: **1** (0.4 mmol, 1 equiv.), ROH (0.8 mL), HCl (conc. in H_2_O, 2.0 mmol, 150 μL), *c* = 0.42 M, Ar degas, irradiation with 2× UVA LEDs for 8 h. Isolated yields are reported.

^*b*^16 h.

^*c*^40 h.

^*d*^24 h, 20% SM along with 8% 2-methyl-6-phenylpyridine and 6% 2,4-dimethyl-6-phenylpyridine observed.

^*e*^410 nm LED was used for 16 h.

^*f*^ROH = ethanol.

^*g*^Et_2_O used instead of EtOH.

^*h*^MTBE used instead of MeOH.

^*i*^THF was used.

^*j*^Tetrahydrofurfuryl alcohol was used.

^*k*^1,4-Dioxane used, **2k** also isolated in 26% yield.

Extending this methodology to other alcohol and ether coupling partners with lepidine was also evaluated (Table 2B). The reaction run in ethanol gave the corresponding ethylated **2k** in 54% yield whereas the use of diethyl ether gave only 15% of the product. Methyl *tert*-butyl ether (MTBE) was found to give **2a** in 34% yield. Interestingly, THF, known to be the best H-atom donors among ethers,^9^ gave the corresponding addition and fragmentation product **2l** in 88% yield. Tetrahydrofurfuryl alcohol gave **2m** in 77% yield, showing regio- and chemoselectivity towards functionalization at the 5-position of the furan. 1,4-Dioxane gave **2n** in 28% yield, where products **2k** and **2n′** were also isolated in 26% and 20% yields.

To our surprise, when using iPrOH with lepidine, the tetrahydroquinoline product **5a** was observed in 31% yield (Table 2C). This is likely a reflection of the reducing characteristics of the ketyl radical derived from iPrOH.^10^ We then extended this light-enabled reduction to other heteroarenes such as methyl and adamantyl functionalized quinolines to produce the desired heterocycles **5b–e** in moderate to excellent yields. Notably, the bromo functionality in **5c** remained intact.^11^ Using 410 nm LEDs, phenanthridine and acridine also gave reduced heterocycles **5f** and **5g** in 53% and 32% (60% brsm), respectively.

A systematic approach to deuterium labeling experiments was performed to help distinguish plausible mechanistic pathways (Table 3A). As expected, under fully deuterated conditions using CD_3_OD and DCl in D_2_O gave **d_3_-2a** in 73% yield (85% brsm, entry 1). When using CD_3_OH with HCl in H_2_O, the **d_2_-2a** in 78% yield (entry 2). Implementation of CH_3_OD and DCl in D_2_O provided the **d-2a** in 77% yield (entry 3). Interestingly, the conditions do not generate ratios of deuterium incorporation where scrambling of benzylic protons is observed in other methodology.^12^ These results likely indicate that the final product derives from an enamine intermediate that is not under equilibrium and is determined by the nature of the broadly exchanging solvent. In order to test if **3a** is a viable intermediate in the production of the final product (**2a**), **3a** was first subjected to the optimized reaction conditions, yielding **2a** in 30% (entry 4). Examining the product generated from **3a** using CD_3_OH with HCl in H_2_O produced **2a** in 54% yield (entry 5). However, **d-2a** was obtained in 51% using CH_3_OD with DCl in D_2_O (entry 6). These experiments support the hypothesis that **3a** is not likely to be a major intermediate in this process under optimized conditions but gives a similar distribution if it is formed as a minor intermediate. Using a mixture of CH_3_OH and CD_3_OH (1 : 1), a ratio of products **2a** : **d_2_-2a** (65 : 35) was obtained thereby giving a kinetic isotope effect (KIE) of 1.86 (entry 7). In addition, a mixture of **1a** and **d-1a** (51 : 49), was treated under optimal conditions to give a KIE of 1.77 (entry 8, see ESI† for details). To compare the corresponding data for ethers, THF and d_8_-THF (1 : 1) were submitted to the optimal conditions, where a mixture of **2l** and **d_7_-2l** were obtained in 54% yield as a 55.5 : 44.5 ratio, providing an observed KIE of 1.20 (entry 9).

**Table 3:** Deuterium labelling experiments

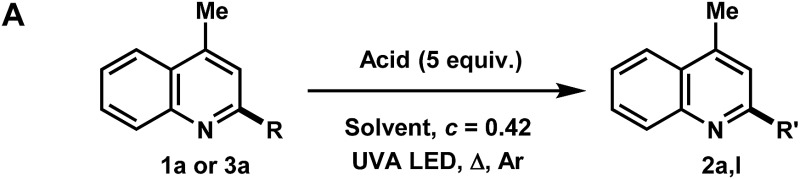						
Entry	R	Acid	Solvent	R′	*t* [h]	**2a** [%]
1	**1a**, H	DCl in D_2_O	CD_3_OD	CD_3_	16	**d_3_-2a**, 73 (85 brsm)
2	**1a**, H	HCl in H_2_O	CD_3_OH	CD_2_H	16	**d_2_-2a**, 78
3	**1a**, H	DCl in D_2_O	CH_3_OD	CDH_2_	16	**d-2a**, 77
4	**3a**, CH_2_OH	HCl in H_2_O	CH_3_OH	CH_3_	8	**2a**, 30
5	**3a**, CH_2_OH	HCl in H_2_O	CD_3_OH	CH_3_	20	**2a**, 54
6	**3a**, CH_2_OH	DCl in D_2_O	CH_3_OD	CDH_2_	20	**d-2a**, 51
7	**1a**, H	HCl in H_2_O	CH_3_OH/CD_3_OH (1 : 1)	CH_3_/CD_2_H (65 : 35)	16	**2a** : **d_2_-2a**, 73 (65 : 35) KIE = 1.86
8	**1a** : **d-1a**, H : D (51 : 49)	HCl in H_2_O	CH_3_OH	CH_3_	1	**2a**, 70 SM H : D (37 : 63) KIE = 1.77^*a*^
9	**1a**, H	HCl in H_2_O	THF/d_8_-THF (1 : 1)	(CH_2_)_4_OH/CHD(CD_2_)_3_OH (57 : 43)	16	**2l** : **d_7_-2l**, 54 (55.5 : 45.5) KIE = 1.20

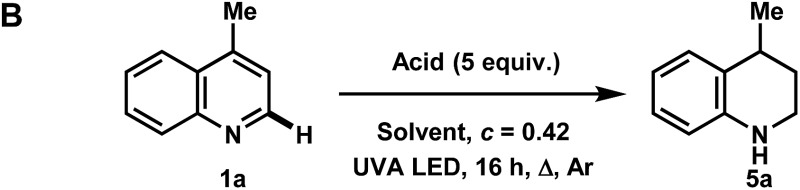			
Entry	Acid	Solvent	Product [%]
1	DCl in D_2_O	(CD_3_)_2_CDOD	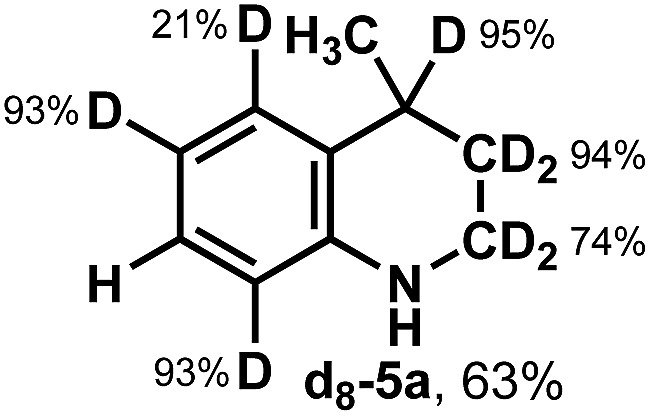
2	HCl in H_2_O	(CD_3_)_2_CDOH	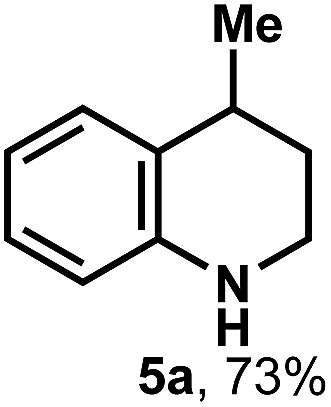
3	DCl in D_2_O	(CH_3_)_2_CHOD	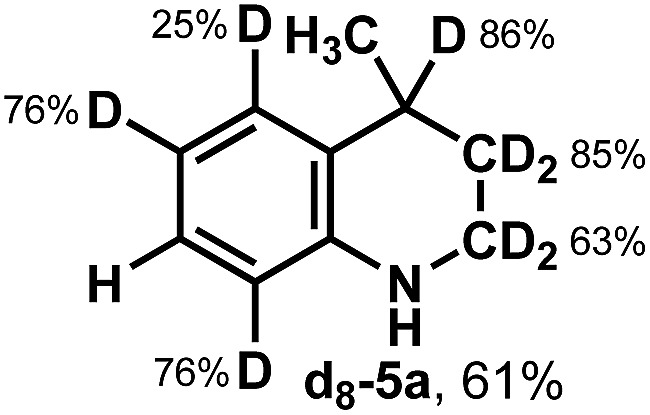

^*a*^Calculated based on remaining SM (see ESI for details).

A similar approach was taken for further understanding of the photochemical reduction of heteroarenes using iPrOH (Table 3B). Using d_8_-iPrOH with DCl in D_2_O for the light mediated reduction of lepidine, **d_8_-5a** was isolated in 63% yield (entry 1). Interestingly, when using d_7_-iPrOH with HCl in H_2_O, deuterium incorporation is not observed in **5a**, isolated in 73% yield (entry 2). Finally, when using iPrOD with DCl in D_2_O (entry 3, 61% yield), **d_8_-5a** was obtained with a similar deuterium distribution as in entry 1. These results are indicative of the excited state protonated lepidine and the following reactive intermediates during formation of **5a** do not undergoing HAT reactions with iPrOH. However, it is possible that HAT may occur on the nitrogen atom, where erosion of deuterium incorporation by the broadly exchanging nature of the N–H bond would occur during purification.

**Fig. 2:**
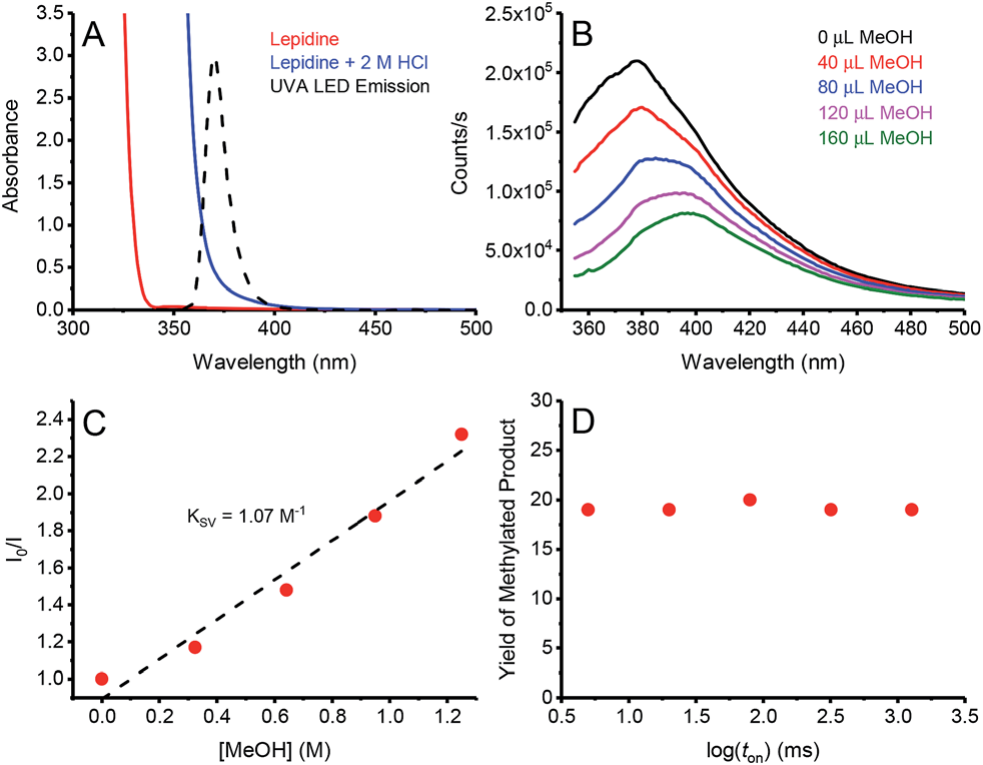
(A) Absorption spectra of lepidine (0.42 M) in MeOH (red), and lepidine (0.42 M) in 2 M HCl in MeOH (blue). When comparing these spectra to the emission profile of the 365 nm LED employed, it becomes evident that lepidine can only be excited in its protonated form under our reaction conditions. (B) Quenching of the emission of lepidine upon increasing concentrations of MeOH in 2 M HCl in MeCN. (C) Corresponding Stern–Volmer plot, the slight curvature likely reflects the small special shift in panel B. (D) Yield of methylated lepidine *versus* the log of the light-on period (*t*_on_). For a full description of the experimental procedure and set-up, see ESI.†

In order to gain insights into the underlying mechanism of these transformations, we performed a series of mechanistic studies. UV-vis absorption studies confirmed lepidine only absorbs at the irradiation wavelengths of our UVA LED in the presence of acid (Fig. 2A). In order to prove that the reaction is initiated by quenching of the lepidine singlet state by MeOH, we performed Stern–Volmer quenching studies. As seen in Fig. 2, the emission of lepidine in 2 M HCl in MeCN was found to be quenched by the addition of MeOH, with a Stern–Volmer constant (*K*_SV_) of 1.07 M^–1^. During our quenching studies, we also observed a slight redshift in the emission maxima of lepidine, which could be due to the change in the environmental conditions upon addition of MeOH. In good agreement, we also observed quenching of lepidine by MeOH when employing H_2_O as the solvent with no change in the emission maximum (see ESI†). Furthermore, the decreased quenching efficiency in H_2_O (0.078 M^–1^) correlate with our previous experimental observations which showed that H_2_O is detrimental to the reactivity of this system (Table 1, entry 2). However, we cannot rule out that a portion of the observed increase in quenching in MeCN is due to the change in the environmental conditions upon addition of MeOH. Clearly a change in the environmental conditions is not the sole cause of the quenched emission, as no quenching would be observed in H_2_O if this were the case. Based on the results of our deuterated studies which showed a KIE of 1.86, we propose that this quenching event proceeds through a proton-coupled electron-transfer (PCeT), forming a protonated lepidine intermediate and a hydroxymethyl radical. Considering the observed KIE of 1.86, it is proposed that this reaction proceeds *via* a sequential PCeT, with electron-transfer occurring first, followed by proton-transfer.^13^ Furthermore, it is unlikely that this quenching event results in the single-electron oxidation of MeOH to form a MeOH radical-cation, as this would not be expected to produce a KIE. It is also possible that this reaction could proceed through a hydrogen atom transfer (HAT) mechanism, however this is also unlikely as we would expect larger KIE values than those observed in this work if a HAT was involved in the rate determining step.^14^

We also examined the steady-state quenching of lepidine's emission by THF, one of the ethers employed in this work (Table 2, **2l**). Once again, quenching of the lepidine emission was observed upon addition of THF (see ESI†), with a *K*_SV_ of 1.08 M^–1^. Due to the observation of minimal KIE in this system (1.25, Table 3A, entry 9), it is proposed that this quenching event proceeds solely through eT, and not through a PCeT event such as the system employing MeOH.

In order to determine if chain propagation is present in the underlying mechanism of these transformations, we decided to perform intermittent illumination studies. It is evident that many in the field of photoredox catalysis understand that one can probe a photo-initiated chain reaction through the use of intermittent illumination. However, as the lifetime of most chains are in the sub-second timescale, current attempts to establish whether or not a photoredox transformation involves a chain by testing the effect of switching the light source on and off on the time scale of minutes are futile. Therefore, a more appropriate test would be the “rotating sector” method, which has been employed in the past to produce intermittent illumination on the appropriate timescale for typical chain reactions.^15^ Recently, the Scaiano group presented an updated version of the “rotating sector” method, employing current LED technologies to pulse LEDs down to the nanosecond timescale, and by using this technique were able to successfully characterize multiple chain reactions.^16^ Using this technique, we were able to demonstrate that the temporal profile of irradiation does not have any effect on the yield of methylated lepidine (Fig. 2D). This indicates the absence of a propagating chain in the underlying mechanism, hinting that a sacrificial amount of lepidine is used as a photocatalyst to promote the photochemical methylation (*vide infra*).

**Fig. 3:**
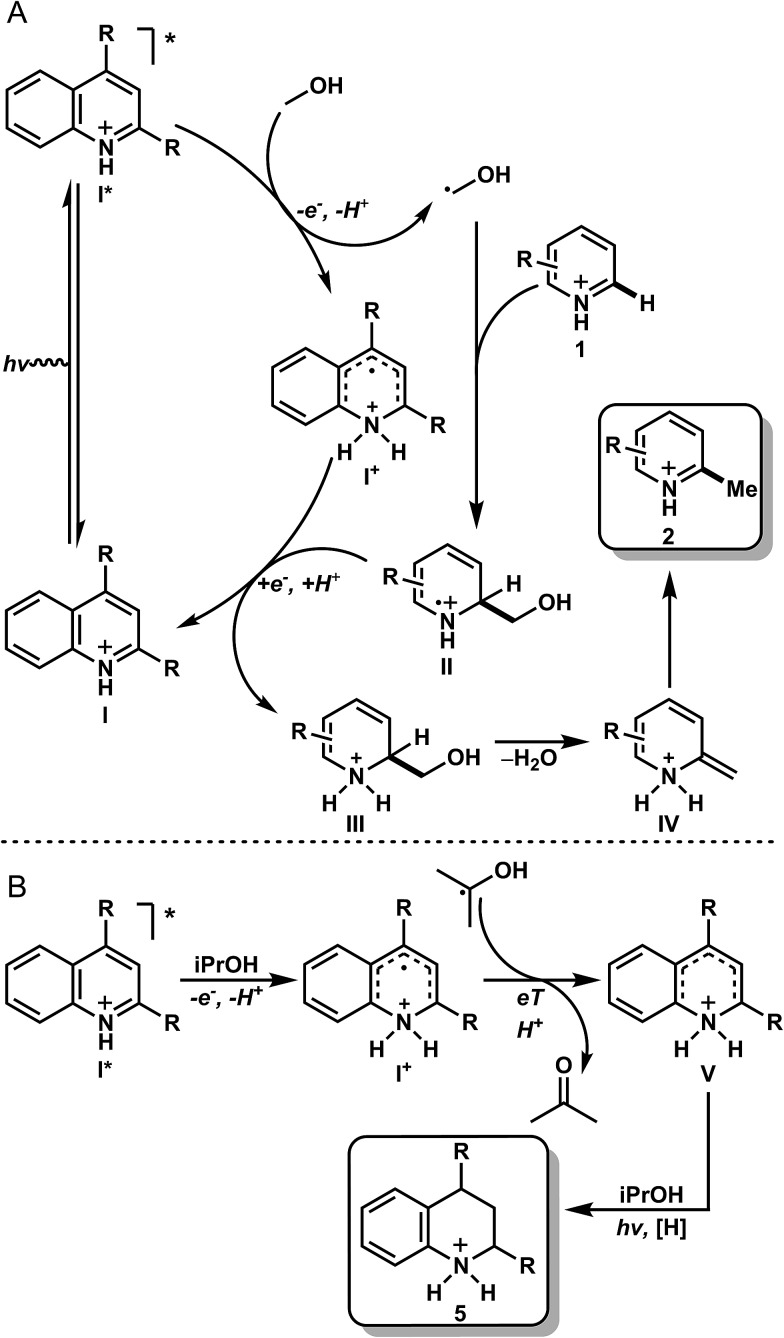
Proposed mechanism for the methylation (A) and reduction (B) of heteroarenes. The same mechanism is operative in both the stoichiometric and catalytic methylation examples.

With the data obtained for the methylation/alkylation of a variety of heteroarenes taken with the mechanistic data, it is proposed that upon protonation, the heteroarene (**I**) is excited to the singlet state (I*), where it can be quenched by MeOH to give a protonated radical intermediate (**I^+^**) and a hydroxymethyl radical (Fig. 3A). This quenching event likely proceeds *via* a sequential PCeT type mechanism stabilized by hydrogen bonding between the heteroarene and methanol, based on the KIEs observed in our deuterated studies. This relatively nucleophilic hydroxymethyl radical can then react readily with an electrophilic protonated heteroarene (**I**), leading to intermediate (**II**). Intermediate (**II**) may then be reduced by intermediate (**I^+^**), giving amino alcohol (**III**) and regenerating the ground state heterocycle (**I**, catalyst or as stoichiometric reactant). This may also proceed *via* PCeT based on the observed KIE in Table 3, entry 8. Intermediate **III** leads to intermediate **IV** by elimination of water, which after tautomerization, gives the methylated product (**2**). Under reducing conditions with iPrOH (Fig. 3B), it is likely that the intermediate (**I^+^**) becomes reduced by the incumbent ketyl radical derived from quenching with iPrOH. Intermediate **V** has several resonance forms under equilibrium, which undergo exchange reactions with the broadly exchanging portion of the solvent as observed with product **d_8_-5a** (Table 3B, entries 1 and 3). Upon further photochemical reduction with iPrOH, the reduced heteroarene **5** is realized. This mechanism of action is distinct from the photomediated reduction of haloarenes and selected arenes HAT mechanism in presence of iPrOH.^17^

Finally, with the results and mechanistic data obtained in this study, we gathered that the methylation of heteroarenes that do not absorb in the UVA may be catalyzed by a heteroarene such as 2,4-diphenylquinoline with 410 nm irradiation (Table 4). Gratifyingly, the transformation using 2,6-di-*tert*-butylpyridine was found to proceed to full conversion in 24 hours using 2.0 mol% of 2,4-diphenylquinoline, yielding 71% of **2o**, whereas less than 10% conversion was observed in the absence of 2,4-diphenylquinoline. Other heteroarenes that were found to either undergo degradation or react poorly in the UVA mediated methodology such as quinaldines were found to be improved in this catalytic process, providing the methylated products **2a–c** in 57%, 71% and 62% yields, respectively. This highlights the advantage of employing a photocatalyst where irradiation wavelengths that avoid direct excitation of the reaction products can be employed. Finally, the photocatalytic methylation of 2,6-diphenylpyridine proceeded to give **2f** in 90% yield. The facile photocatalytic methylation described demonstrates the high potential for this system to have broad applicability in the alkylation of a variety of heteroarenes and will be investigated further.

**Table 4:** Catalytic methylation of heteroarenes^*a*^

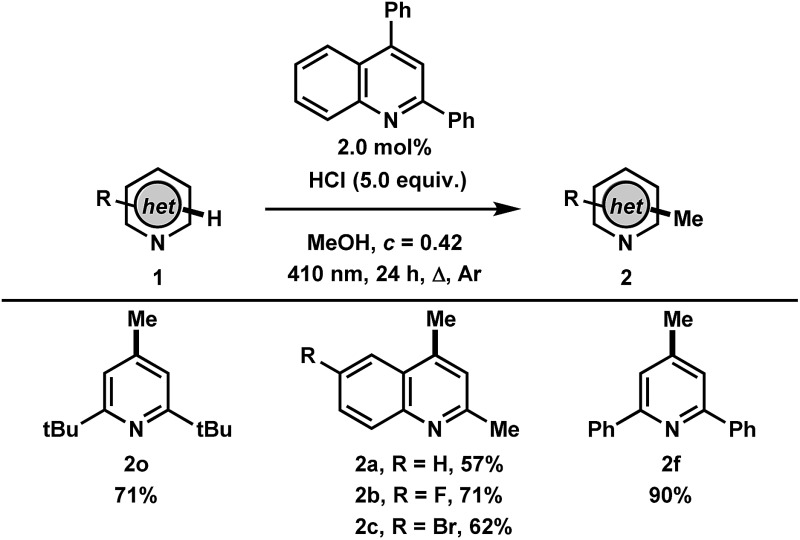

^*a*^Procedure: **1** (0.4 mmol, 1 equiv.), 2,4-diphenylquinoline (0.008 mmol, 2 mol%, 2.3 mg), MeOH (0.8 mL), HCl (conc. in H_2_O, 2.0 mmol, 150 μL), *c* = 0.42 M, Ar degas, irradiation with 1× 410 nm LED for 24 h. Isolated yields are reported.

## Conclusions

In summary, the photochemical alkylation of quinolines, pyridines, and phenanthridine from alcohols and ethers was described. Deuterium labelling studies showed the ability to control the formation of CD_3_, CD_2_H, and CDH_2_ products, indicating the reaction likely proceeds through an enamine intermediate. We also reported the first photochemical organic-mediated reduction of heteroarenes using simply iPrOH and HCl. This study also provides mechanistic insights to a complex reaction pathway and the possibility of a broadly applicable catalytic system that will be disclosed in due course.‡
‡Experimental procedures and analytical data for all new compounds can be found in the ESI†.


## Conflicts of interest

There are no conflicts to declare.
