# Supplementary material for: The photochemical alkylation and reduction of heteroarenes
Source: Chem Sci. 2017 Sep 11;8(11):7412–8. doi: 10.1039/c7sc03768f (PMC5674141; doi:10.1039/c7sc03768f)
Supplement: Supplementary file 1 [file SC-008-C7SC03768F-s001.pdf]

Electronic Supplementary Material (ESI) for Chemical Science.
This journal is © The Royal Society of Chemistry 2017

Electronic Supporting Information for

Photochemical Alkylation and Reduction of Heteroarenes

T. M[c]Callum, S. P. Pitre, M. Morin, J. C. Scaiano* and L. Barriault*

Centre for Catalysis, Research and Innovation, Department of Chemistry and Biomolecular Sciences, University of Ottawa, Ottawa, Ontario, Canada.
*Correspondence to: scaiano@photo.chem.uottawa.ca, lbarriau@uottawa.ca

Table of Contents

1. General Information 2
2. General Procedures 2
    GP1 – Preparation of Alkylated or Reduced Heteroarenes 2
    GP2 – Photocatalytic Methylation of Heteroarenes 2
3. Characterization of Products 3
4. Kinetic Isotope Effect Experiments 14
5. Steady-State Fluorescence Quenching of Lepidine 15
6. Intermittent Illumination Experimental 16
7. References 17
8. NMR Spectra 18

1. General Information

All reactions were performed in Pyrex glassware equipped with a magnetic stir bar, capped with a septum, unless otherwise indicated. All commercial reagents were used without further purification, unless otherwise noted. Reactions were monitored by thin layer chromatography (TLC) analysis. TLC plates were viewed under UV light and stained with potassium permanganate or *p*-anisaldehyde staining solutions. Yields refer to products isolated after purification, unless otherwise stated. Proton nuclear magnetic resonance (^{1}H NMR) spectra were recorded on a Bruker AMX 400 MHz. NMR samples were dissolved in chloroform-*d* (unless specified otherwise) and chemical shifts are reported in ppm referenced to residual undeuterated solvent. Carbon nuclear magnetic resonance (^{13}C NMR) spectra were recorded on the same Bruker instrument (101 MHz). IR spectra were recorded with an Agilent Technologies Cary 630 FTIR Spectrometer equipped with a diamond ATR module. HRMS were obtained on a Kratos Analytical Concept instrument (University of Ottawa Mass Spectrum Centre). UV-vis absorption spectra were recorded using an Agilent Cary 7000.

2. General Procedure

General Procedure 1 (GP1). *Preparation of Alkylated and Reduced Heteroarenes.* To an 8 mL screw-topped Pyrex reaction vessel was added the heteroarene (0.4 mmol, 1.0 equiv), alcohol/ether (0.8 mL) and then concentrated HCl (2.0 mmol, 5.0 equiv, 150 μL). The solution was degassed by sparging under argon for 5 minutes, sealed with parafilm, and irradiated with 2 X UVA LEDs (365 nm) or 1 X 410 nm LEDs (depending on the absorbance profile of the protonated heterocycle) at a distance of 1 mm for 8 hours. Upon completion, the solution was poured into a separatory funnel with 1 M NaOH and extracted with DCM. The combined organic extracts were dried over Na_2SO_4, filtered, and concentrated. The crude residue was further purified by flash column chromatography (0-100% EtOAc:Hex or 0-20% MeOH:DCM), where relevant fractions were combined, concentrated and characterized by proton and carbon NMR (400 and 101 MHz, respectively), HR-MS, and IR.

General Procedure 2 (GP2). *Photocatalytic Methylation of Heteroarenes*. To an 8 mL screw-topped Pyrex reaction vessel was added the heteroarene (0.4 mmol, 1.0 equiv), 2,4-diphenylquinoline (0.008, 2 mol%, 2.3 mg), MeOH (0.8 mL) and then concentrated HCl (2.0 mmol, 5.0 equiv, 150 μL). The solution was degassed by sparging under argon for 5 minutes, sealed with parafilm, and irradiated with 1 X 410 nm LEDs at a distance of 1 mm for 24 hours. Upon completion, the solution was poured into a separatory funnel with 1 M NaOH and extracted with DCM. The combined organic extracts were dried over Na_2SO_4, filtered, and concentrated. The crude residue was further purified by flash column chromatography (0-100% EtOAc:Hex or 0-20% MeOH:DCM), where relevant fractions were combined, concentrated and characterized by proton and carbon NMR (400 and 101 MHz, respectively), HR-MS, and IR. 2,4-diphenylquinoline was synthesized according to literature procedure.[1]

3. Characterization of Materials and Products

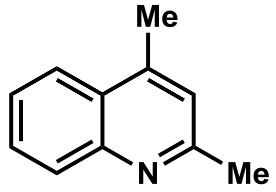

2,4-dimethylquinoline (2a)

Synthesized according to GP1 and characterized according to NMR comparison.[2]

^{1}H NMR (400 MHz, $CDCl_3$): δ = 8.02 (dd, *J* = 8.4, 0.6 Hz, 1H), 7.95 (dd, *J* = 8.4, 0.6 Hz, 1H), 7.68 (ddd, *J* = 8.4, 6.9, 1.4 Hz, 1H), 7.51 (ddd, *J* = 8.2, 6.9, 1.2 Hz, 1H), 7.14 (s, 1H), 2.70 (s, 3H), 2.67 (s, 3H) ppm; **^{13}C NMR** (101 MHz, $CDCl_3$): δ = 158.7 (C), 147.7 (C), 144.1 (C), 129.2 (CH), 129.1 (CH), 126.5 (C), 125.4 (CH), 123.6 (CH), 122.7 (CH), 25.2 (CH_3), 18.6 (CH_3) ppm.

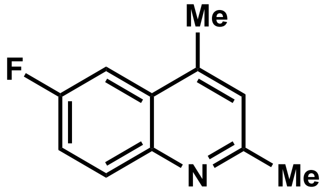

6-fluoro-2,4-dimethylquinoline (2b)

Synthesized according to GP1.

IR (neat, cm^{-1}): 3021(m), 2974(m), 2925(m), 1609(vs), 1509(vs), 1403(s), 1371(vs), 845(vs), 835(vs); **^{1}H NMR** (400 MHz, $CDCl_3$): δ = 8.00 (dd, *J* = 9.2, 5.5 Hz, 1H), 7.52 (dd, *J* = 9.9, 2.8 Hz, 1H), 7.43 (ddd, *J* = 9.1, 8.2, 2.8 Hz, 1H), 7.14 (s, 1H), 2.68 (s, 3H), 2.60 (s, 3H) ppm; **^{13}C NMR** (101 MHz, $CDCl_3$): δ = 159.9 (d, *J* = 246.1 Hz, CH), 157.9 (d, *J* = 2.6 Hz, CH), 144.8 (C), 143.5 (d, *J* = 5.5 Hz, C), 131.4 (d, *J* = 8.8 Hz, CH), 127.2 (d, *J* = 9.2 Hz, C), 123.2 (CH), 118.9 (d, *J* = 25.7 Hz, CH), 107.2 (d, *J* = 22.4 Hz, CH), 25.0 (CH_3), 18.5 (CH_3) ppm; **HRMS** (EI): m/z calc'd for $C_{11}H_{10}FN$ [M^+] 175.0797, found 175.0797.

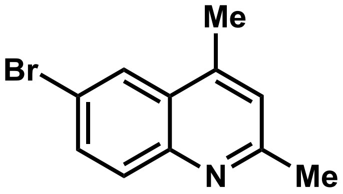

6-bromo-2,4-dimethylquinoline (2c)

Synthesized according to GP1 and characterized according to NMR comparison.[2]

^{1}H NMR (400 MHz, $CDCl_3$): δ = 8.09 (d, *J* = 2.3 Hz, 1H), 7.88 (d, *J* = 8.9 Hz, 1H), 7.73 (dd, *J* = 8.9, 2.2 Hz, 1H), 7.15 (s, 1H), 2.68 (s, 3H), 2.63 (d, *J* = 0.9 Hz, 3H) ppm; **^{13}C NMR** (101 MHz, $CDCl_3$): δ = 159.2 (C), 146.3 (C), 143.3 (C), 132.4 (CH), 130.9 (CH), 127.9 (C), 126.1 (CH), 123.4 (CH), 119.3 (C), 25.2 (CH_3), 18.5 (CH_3) ppm.

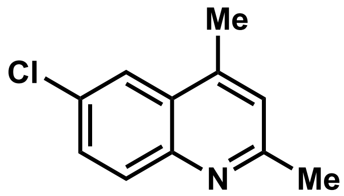

6-chloro-2,4-dimethylquinoline (2d)
Synthesized according to GP1.
IR (neat, cm^{-1}): 2921(m), 2853(m), 1606(vs), 1494(s), 1439(s), 1375(s), 1088(s), 872(s), 839(vs); **^{1}H NMR** (400 MHz, $CDCl_3$): δ = 7.93 (d, *J* = 8.9 Hz, 1H), 7.89 (d, *J* = 2.3 Hz, 1H), 7.59 (dd, *J* = 8.9, 2.4 Hz, 1H), 7.13 (s, 1H), 2.67 (s, 3H), 2.61 (d, *J* = 0.9 Hz, 3H) ppm; **^{13}C NMR** (101 MHz, $CDCl_3$): δ = 159.0 (C), 146.1 (C), 143.3 (C), 131.1 (C), 130.7 (CH), 129.8 (CH), 127.3 (C), 123.4 (CH), 122.7 (CH), 25.1 (CH_3), 18.5 (CH_3) ppm; **HRMS** (EI): m/z calc'd for $C_{11}H_{10}ClN$ [M^+] 191.0502, found 191.0495.

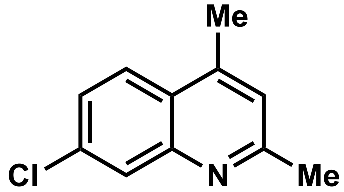

7-chloro-2,4-dimethylquinoline (2e)
Synthesized according to GP1.
IR (neat, cm^{-1}): 2920(m), 2855(m), 1603(vs), 1494(s), 1404(s), 898(s), 812(s); **^{1}H NMR** (400 MHz, $CDCl_3$): δ = 8.01 (d, *J* = 2.1 Hz, 1H), 7.86 (d, *J* = 8.8 Hz, 1H), 7.44 (dd, *J* = 8.9, 2.1 Hz, 1H), 7.12 (s, 1H), 2.68 (s, 3H), 2.64 (s, 3H) ppm; **^{13}C NMR** (101 MHz, $CDCl_3$): δ = 159.9 (C), 148.2 (C), 144.2 (C), 134.8 (C), 128.1 (CH), 126.3 (CH), 125.0 (C), 124.9 (CH), 122.9 (CH), 25.2 (CH_3), 18.5 (CH_3) ppm; **HRMS** (EI): m/z calc'd for $C_{11}H_{10}ClN$ [M^+] 191.0502, found 191.0513.

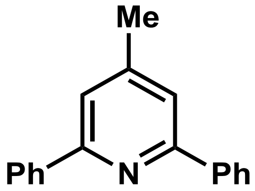

4-methyl-2,6-diphenylpyridine (2f)
Synthesized according to GP1 and characterized according to NMR comparison.[3]
^{1}H NMR (400 MHz, $CDCl_3$): δ = 8.18-8.14 (m, 4H), 7.54 (d, *J* = 0.8 Hz, 2H), 7.53-7.48 (m, 4H), 7.46-7.41 (m, 2H), 2.50 (s, 3H) ppm; **^{13}C NMR** (101 MHz, $CDCl_3$): δ = 156.8 (2 X C), 148.3 (C), 139.6 (2 X C), 128.8 (2 X CH), 128.6 (4 X CH), 127.0 (4 X CH), 119.7 (2 X CH), 21.4 (CH_3) ppm.

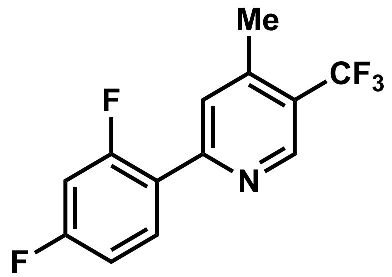

2-(2,4-difluorophenyl)-4-methyl-5-(trifluoromethyl)pyridine (2g)
Synthesized according to GP1.
IR (neat, cm^{-1}): 2930(m), 1600(s), 1490(m), 1323(vs), 1121(vs), 1037(s), 970(s), 849(s), 721(m); **^{1}H NMR** (400 MHz, $CDCl_3$): δ = 8.86 (s, 1H), 8.06 (td, *J* = 8.9, 6.6 Hz, 1H), 7.73-7.68 (m, 1H), 7.03 (dddd, *J* = 8.8, 7.8, 2.5, 1.0 Hz, 1H), 6.94 (ddd, *J* = 11.2, 8.6, 2.4 Hz, 1H), 2.56 (d, *J* = 1.0 Hz, 3H) ppm; **^{13}C NMR** (101 MHz, $CDCl_3$): δ = 163.8 (dd, *J* = 252.4, 12.5 Hz, CF), 160.8 (dd, *J* = 253.1, 11.7 Hz, CF), 155.5 (C), 146.7 (C), 146.6 (q, *J* = 5.9 Hz, CH), 132.4 (dd, *J* = 9.9, 4.0 Hz, CH), 126.5 (d, *J* = 10.3 Hz, CH), 124.0 (q, *J* = 273.6 Hz, CF_3), 123.9 (q, *J* = 30.1 Hz, C), 122.5 (dd, *J* = 11.7, 3.7 Hz, C), 112.1 (dd, *J* = 21.3, 3.7 Hz, CH), 104.5 (dd, *J* = 27.1, 25.7 Hz, CH), 19.2 (dd, *J* = 3.7, 1.5 Hz, CH_3) ppm; **HRMS** (EI): m/z calc'd for $C_{13}H_8F_5N$ [M^+] 273.0577, found 273.0578.

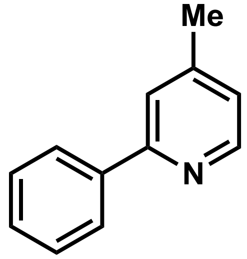

4-methyl-2-phenylquinoline (2h)
Synthesized according to GP1 and characterized according to NMR comparison (containing 20% SM not separable by column chromatography).[2]
^{1}H NMR (400 MHz, $CDCl_3$): δ = 8.53 (d, *J* = 5.0 Hz, 1H), 8.00-7.93 (m, 2H), 7.53 (dt, *J* = 1.5, 0.7 Hz, 1H), 7.48-7.41 (m, 2H), 7.41-7.35 (m, 1H), 7.04 (ddd, *J* = 5.0, 1.6, 0.7 Hz, 1H), 2.40 (s, 3H) ppm; **^{13}C NMR** (101 MHz, $CDCl_3$): δ = 157.4 (C), 149.4 (CH), 147.7 (C), 139.5 (C), 128.8 (CH), 128.6 (2 X CH), 126.9 (2 X CH), 123.1 (CH), 121.5 (CH), 21.2 (CH_3) ppm.

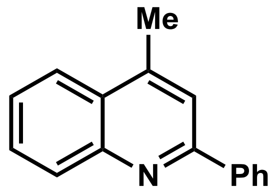

4-methyl-2-phenylquinoline (2i)

Synthesized according to GP1 and characterized according to NMR comparison.[2]

^{1}H NMR (400 MHz, $CDCl_3$): δ = 8.21-8.14 (m, 3H), 8.02 (dd, *J* = 8.4, 1.0 Hz, 1H), 7.76-7.71 (m, 2H), 7.59-7.51 (m, 3H), 7.50-7.44 (m, 1H), 2.79 (d, *J* = 0.8 Hz, 3H) ppm; **^{13}C NMR** (101 MHz, $CDCl_3$): δ = 157.1 (C), 148.2 (C), 144.8 (C), 139.9 (C), 130.3 (CH), 129.3 (CH), 129.2 (CH), 128.8 (2 X CH), 127.5 (2 X CH), 127.3 (C), 126.0 (CH), 123.6 (CH), 119.8 (CH), 19.0 (CH_3) ppm.

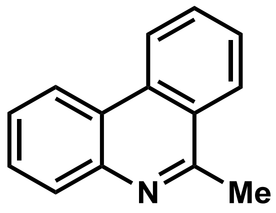

6-methylphenanthridine (2j)

Synthesized according to GP1 and characterized according to NMR comparison.[2]

^{1}H NMR (400 MHz, $CDCl_3$): δ = 8.62-8.56 (m, 1H), 8.51 (dd, *J* = 8.2, 1.4 Hz, 1H), 8.18 (dd, *J* = 8.2, 1.4 Hz, 1H), 8.11 (dd, *J* = 8.0, 1.0 Hz, 1H), 7.81 (ddd, *J* = 8.3, 7.1, 1.3 Hz, 1H), 7.71 (ddd, *J* = 8.2, 7.0, 1.5 Hz, 1H), 7.67 (ddd, *J* = 8.1, 7.0, 1.2 Hz, 1H), 7.61 (ddd, *J* = 8.2, 7.1, 1.4 Hz, 1H), 3.03 (s, 3H) ppm; **^{13}C NMR** (101 MHz, $CDCl_3$): δ = 158.8 (C), 143.7 (C), 132.5 (C), 130.4 (CH), 129.3 (CH), 128.5 (CH), 127.2 (CH), 126.4 (CH), 126.2 (CH), 125.8 (C), 123.7 (C), 122.2 (CH), 121.9 (CH), 23.3 (CH_3) ppm.

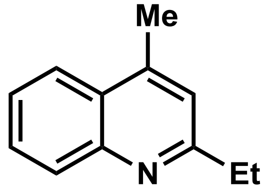

2-ethyl-4-methylquinoline (2k)

Synthesized according to GP1 and characterized according to NMR comparison.[4]

^{1}H NMR (400 MHz, $CDCl_3$): δ = 8.09-8.02 (m, 1H), 7.96 (dd, *J* = 8.3, 1.1 Hz, 1H), 7.68 (ddd, *J* = 8.4, 6.9, 1.4 Hz, 1H), 7.51 (ddd, *J* = 8.3, 6.9, 1.3 Hz, 1H), 7.19-7.15 (m, 1H), 2.97 (q, *J* = 7.6 Hz, 2H), 2.69 (d, *J* = 1.0 Hz, 3H), 1.40 (t, *J* = 7.7 Hz, 3H) ppm; **^{13}C NMR** (101 MHz, $CDCl_3$): δ = 163.7 (C), 147.7 (C), 144.3 (C), 129.4 (CH), 129.0 (CH), 126.8 (C), 125.4 (CH), 123.6 (CH), 121.5 (CH), 32.2 (CH_2), 18.7 (CH_3), 14.0 (CH_3) ppm.

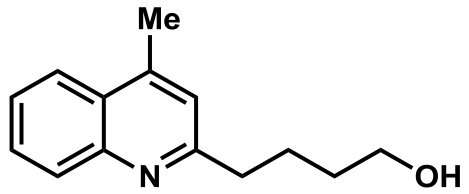

4-(4-methylquinolin-2-yl)butan-1-ol (2l)
Synthesized according to GP1.
IR (neat, cm^{-1}): 3286(br), 2932(m), 2862(m), 1604(s), 1062(m), 780(s); **^{1}H NMR** (400 MHz, $CDCl_3$): δ = 8.03 (d, *J* = 8.4 Hz, 1H), 7.95 (d, *J* = 8.2 Hz, 1H), 7.67 (td, *J* = 7.1, 1.2 Hz, 1H), 7.51 (td, *J* = 7.3, 1.2 Hz, 1H), 7.14 (s, 1H), 3.71 (t, *J* = 6.3 Hz, 2H), 3.14 (br. s., 1H), 2.98 (t, *J* = 7.4 Hz, 2H), 2.67 (s, 3H), 1.94 (quin, *J* = 7.1 Hz, 2H), 1.70 (quin, *J* = 7.1 Hz, 2H) ppm; **^{13}C NMR** (101 MHz, $CDCl_3$): δ = 162.2 (C), 147.4 (C), 144.5 (C), 129.2 (CH), 129.1 (CH), 126.8 (C), 125.5 (CH), 123.6 (CH), 122.2 (CH), 62.1 (CH_2), 38.1 (CH_2), 32.2 (CH_2), 25.4 (CH_2), 18.7 (CH_3) ppm; **HRMS** (EI): m/z calc'd for $C_{14}H_{17}NO$ [M^+] 215.1310, found 215.1301.

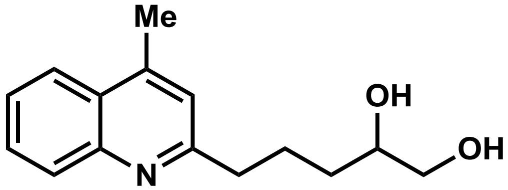

5-(4-methylquinolin-2-yl)pentane-1,2-diol (2m)
Synthesized according to GP1.
IR (neat, cm^{-1}): 3390(br), 2926(m), 2863(m), 1604(s), 1449(m), 1056(s), 760(vs); **^{1}H NMR** (400 MHz, $CDCl_3$): δ = 8.03 (dd, *J* = 8.4, 0.6 Hz, 1H), 7.96 (dd, *J* = 8.3, 0.9 Hz, 1H), 7.68 (ddd, *J* = 8.4, 6.9, 1.4 Hz, 1H), 7.52 (ddd, *J* = 8.3, 7.0, 1.2 Hz, 1H), 7.14 (d, *J* = 0.6 Hz, 1H), 3.78 (m, 1H), 3.69-3.61 (m, 1H), 3.53-3.47 (m, 1H), 3.01 (t, *J* = 7.1 Hz, 2H), 2.68 (d, *J* = 0.9 Hz, 3H), 1.99 (quin, *J* = 7.2 Hz, 2H), 1.60-1.53 (m, 2H) ppm; **^{13}C NMR** (101 MHz, $CDCl_3$): δ = 161.8 (C), 147.1 (C), 144.9 (C), 129.3 (CH), 128.8 (CH), 126.8 (C), 125.7 (CH), 123.6 (CH), 122.3 (CH), 71.4 (CH), 66.9 (CH_2), 37.7 (CH_2), 32.4 (CH_2), 24.6 (CH_2), 18.7 (CH_3) ppm; **HRMS** (EI): m/z calc'd for $C_{15}H_{19}NO_2$ [M^+] 245.1416, found 245.1408.

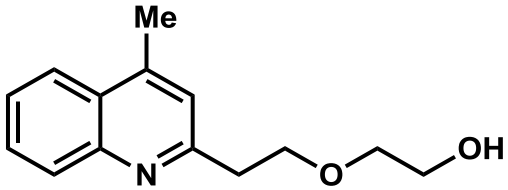

2-(2-(4-methylquinolin-2-yl)ethoxy)ethan-1-ol (2n)
Synthesized according to GP1.
IR (neat, cm^{-1}): 3352(br), 2918(m), 2861(m), 1604(m), 1120(vs), 1060(m), 761(s); **^{1}H NMR** (400 MHz, $CDCl_3$): δ = 8.09 (d, *J* = 8.4 Hz, 1H), 7.96 (d, *J* = 8.2 Hz, 1H), 7.68 (ddd, *J* = 8.1, 7.0, 1.2 Hz, 1H), 7.52 (ddd, *J* = 8.2, 7.0, 1.2 Hz, 1H), 7.17 (s, 1H), 4.28 (br. s., 1H), 4.03 (t, *J* = 6.1 Hz, 2H), 3.77-3.73 (m, 2H), 3.69-3.65 (m, 2H), 3.22 (t, *J* = 6.1 Hz, 2H), 2.68 (s, 3H) ppm; **^{13}C NMR** (101 MHz, $CDCl_3$): δ = 159.3 (C), 147.5 (C), 144.7 (C), 129.3 (CH), 129.1 (CH), 126.9 (C), 125.7 (CH), 123.6 (CH), 122.6 (CH), 72.0 (CH_2), 69.3 (CH_2), 61.8 (CH_2), 38.5 (CH_2), 18.6 (CH_3) ppm; **HRMS** (EI): m/z calc'd for $C_{14}H_{17}NO_2$ [M^+-C_2H_5O] 186.0919, found 186.0923.

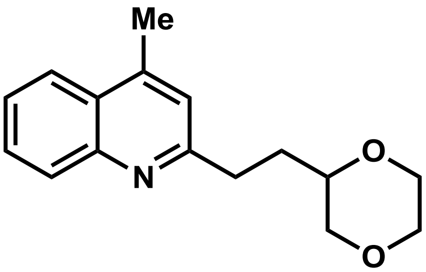

2-(2-(1,4-dioxan-2-yl)ethyl)-4-methylquinoline (2n')

Synthesized according to GP1.

IR (neat, cm^{-1}): 2957(m), 2916(m), 2850(m), 1603(s), 1122(vs), 760(m); **^{1}H NMR** (400 MHz, $CDCl_3$): δ = 8.00 (d, *J* = 7.8 Hz, 1H), 7.93 (dd, *J* = 8.4, 0.8 Hz, 1H), 7.65 (ddd, *J* = 8.4, 6.9, 1.4 Hz, 1H), 7.49 (ddd, *J* = 8.2, 6.9, 1.2 Hz, 1H), 7.13 (s, 1H), 3.81-3.66 (m, 4H), 3.64-3.55 (m, 2H), 3.32 (dd, *J* = 11.3, 10.0 Hz, 1H), 3.12-3.01 (m, 1H), 2.99-2.89 (m, 1H), 2.65 (d, *J* = 0.8 Hz, 3H), 1.93-1.84 (m, 2H) ppm; **^{13}C NMR** (101 MHz, $CDCl_3$): δ = 161.5 (C), 147.8 (C), 144.4 (C), 129.4 (CH), 129.1 (CH), 126.8 (C), 125.5 (CH), 123.6 (CH), 122.1 (CH), 74.8 (CH), 71.3 (CH_2), 66.8 (CH_2), 66.5 (CH_2), 34.3 (CH_2), 31.4 (CH_2), 18.7 (CH_3) ppm; **HRMS** (EI): m/z calc'd for $C_{16}H_{19}NO_2$ [M^+] 257.1416, found 257.1419.

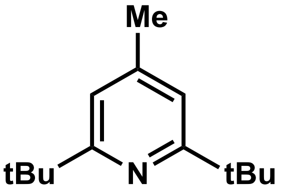

2,6-di-tert-butyl-4-methylpyridine (2o)

Synthesized according to GP1 and characterized according to NMR comparison.[5]

^{1}H NMR (400 MHz, $CDCl_3$): δ = 6.92 (d, *J* = 0.6 Hz, 2H), 2.33-2.30 (m, 3H), 1.34 (s, 18H) ppm; **^{13}C NMR** (101 MHz, $CDCl_3$): δ = 167.4 (2 X C), 146.4 (C), 116.2 (2 X CH), 37.4 (2 X C), 30.1 (6 X CH_3), 21.5 (CH_3) ppm.

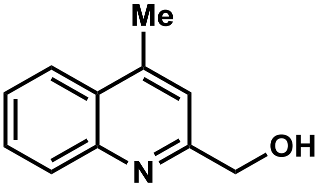

(4-methylquinolin-2-yl)methanol (3a)

Synthesized according to GP1 and characterized according to NMR comparison.[6]

^{1}H NMR (400 MHz, $CDCl_3$): δ = 8.08 (d, *J* = 8.4 Hz, 1H), 7.99 (dd, *J* = 8.3, 1.0 Hz, 1H), 7.72 (ddd, *J* = 8.4, 6.9, 1.4 Hz, 1H), 7.61-7.54 (m, 1H), 7.13 (s, 1H), 4.88 (s, 2H), 4.50 (br. s., 1H), 2.71 (d, *J* = 0.8 Hz, 3H) ppm; **^{13}C NMR** (101 MHz, $CDCl_3$): δ = 158.5 (C), 146.5 (C), 145.0 (C), 129.4 (CH), 129.2 (CH), 127.6 (C), 126.1 (CH), 123.8 (CH), 118.9 (CH), 64.0 (CH_2), 18.8 (CH_3) ppm.

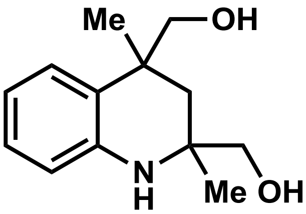

(2,4-dimethyl-1,2,3,4-tetrahydroquinoline-2,4-diyl)dimethanol (4a, *d.r.* 2:1)
Synthesized according to GP1.
IR (neat, cm^{-1}): 3355(br), 2960(m), 2929(m), 2860(m), 1604(m), 1482(s), 1312(m), 1030(vs), 752(vs); **Major: ^{1}H NMR** (400 MHz, $CDCl_3$): δ = 7.19-7.13 (m, 1H), 7.06-6.99 (m, 1H), 6.77-6.72 (m, 1H), 6.58-6.54 (m, 1H), 3.65-3.36 (m, 4H), 2.02 (d, *J* = 14.1 Hz, 1H), 1.60 (d, *J* = 14.1 Hz, 1H), 1.31 (s, 3H), 1.27 (s, 3H) ppm; **Major: ^{13}C NMR** (101 MHz, $CDCl_3$): δ = 144.1 (C), 127.4 (CH), 126.5 (C), 126.4 (CH), 118.4 (CH), 115.6 (CH), 71.4 (CH_2), 68.9 (CH_2), 53.0 (C), 40.6 (CH_2), 37.5 (C), 26.5 (CH_3), 26.1 (CH_3) ppm; **Minor: ^{1}H NMR** (400 MHz, $CDCl_3$): δ = 7.19-7.13 (m, 1H), 7.06-6.99 (m, 1H), 6.77-6.72 (m, 1H), 6.58-6.54 (m, 1H), 3.65-3.36 (m, 4H), 2.30 (d, *J* = 14.1 Hz, 1H), 1.41 (d, *J* = 14.1 Hz, 1H), 1.35 (s, 3H), 1.23 (s, 3H) ppm; **Minor: ^{13}C NMR** (101 MHz, $CDCl_3$): δ = 143.9 (C), 127.3 (CH), 126.4 (C), 126.3 (CH), 118.4 (CH), 115.7 (CH), 71.6 (CH_2), 70.9 (CH_2), 53.2 (C), 39.1 (CH_2), 37.4 (C), 27.0 (CH_3), 26.1 (CH_3) ppm; **HRMS** (EI): m/z calc'd for $C_{13}H_{19}NO_2$ [M^+] 221.1416, found 221.1432.

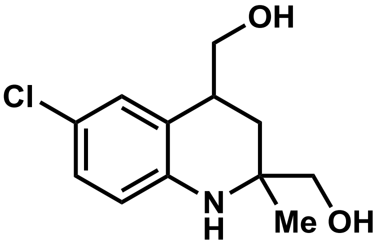

(6-chloro-2-methyl-1,2,3,4-tetrahydroquinoline-2,4-diyl)dimethanol (*d.r.* 85:15)
Synthesized according to GP1.
IR (neat, cm^{-1}): 3339(br), 2967(m), 2927(m), 2874(m), 1602(m), 1487v(s), 1301(m), 1034(s), 812(s); **^{1}H NMR** (400 MHz, $CDCl_3$): δ = 7.21 (dd, *J* = 2.4, 1.0 Hz, 1H, minor), 7.18 (dd, *J* = 2.4, 1.0 Hz, 1H, major), 7.03-6.93 (m, 1H), 6.51-6.45 (m, 1H), 4.03-3.89 (m, 2H), 3.87 (br. s., 1H), 3.52-3.42 (m, 2H), 3.06 (sxt, *J* = 5.6 Hz, 1H, minor), 2.95 (sxt, *J* = 5.7 Hz, 1H, major), 2.02 (dd, *J* = 13.5, 6.1 Hz, 1H), 1.77 (br. s., 1H), 1.68 (dd, *J* = 13.5, 11.5 Hz, 1H), 1.58 (br. s., 1H), 1.28 (s, 3H) ppm; **^{13}C NMR** (101 MHz, $CDCl_3$): δ = 142.9 (C), 127.3 (CH), 126.6 (CH), 122.5 (C), 122.1 (C), 116.1 (CH), 68.0 (CH_2), 65.5 (CH_2), 52.5 (C), 35.3 (CH), 33.9 (CH_2), 26.0 (CH_3) ppm; **HRMS** (EI): m/z calc'd for $C_{12}H_{16}ClNO_2$ [M^+] 241.0870, found 241.0853.

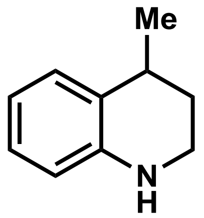

4-methyl-1,2,3,4-tetrahydroquinoline (5a)
Synthesized according to GP1 and characterized according to NMR comparison (containing ~10% of impurities not separable by column chromatography).[7]
^{1}H NMR (400 MHz, $CDCl_3$): δ = 7.07 (d, *J* = 7.6 Hz, 1H), 7.00-6.94 (m, 1H), 6.64 (td, *J* = 7.4, 1.2 Hz, 1H), 6.49 (dd, *J* = 8.0, 1.0 Hz, 1H), 3.87 (br. s., 1H), 3.39-3.20 (m, 2H), 2.93 (sxt, *J* = 6.4 Hz, 1H), 2.05-1.95 (m, 1H), 1.75-1.65 (m, 1H), 1.30 (d, *J* = 7.1 Hz, 3H) ppm; **^{13}C NMR** (101 MHz, $CDCl_3$): δ = 144.2 (C), 128.4 (CH), 126.7 (CH), 126.6 (C), 116.9 (CH), 114.1 (CH), 39.0 (CH_2), 30.2 (CH), 29.9 (CH_2), 22.6 (CH_3) ppm.

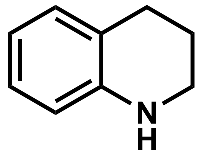

1,2,3,4-tetrahydroquinoline (2b)
Synthesized according to GP1 and characterized according to NMR comparison.[7]
^{1}H NMR (400 MHz, $CDCl_3$): δ = 7.03-6.92 (m, 2H), 6.61 (td, *J* = 7.4, 1.1 Hz, 1H), 6.53-6.44 (m, 1H), 3.83 (br. s., 1H), 3.38-3.25 (m, 2H), 2.78 (t, *J* = 6.5 Hz, 2H), 2.01-1.91 (m, 2H) ppm; **^{13}C NMR** (101 MHz, $CDCl_3$): δ = 144.7 (C), 129.5 (CH), 126.7 (CH), 121.4 (C), 116.9 (CH), 114.2 (CH), 42.0 (CH_2), 26.9 (CH_2), 22.2 (CH_2) ppm.

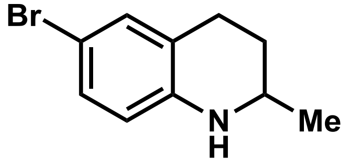

6-bromo-2-methyl-1,2,3,4-tetrahydroquinoline (5c)
Synthesized according to GP1 and characterized according to NMR comparison.[8]
^{1}H NMR (400 MHz, $CDCl_3$): δ = 7.10-7.00 (m, 2H), 6.35 (d, *J* = 8.4 Hz, 1H), 3.72 (br. s., 1H), 3.39 (dtq, *J* = 9.7, 6.4, 3.3 Hz, 1H), 2.81 (ddd, *J* = 16.7, 11.3, 5.6 Hz, 1H), 2.75-2.65 (m, 1H), 1.98-1.85 (m, 1H), 1.55 (dddd, *J* = 12.9, 11.4, 9.9, 5.3 Hz, 1H), 1.21 (d, *J* = 6.3 Hz, 3H) ppm; **^{13}C NMR** (101 MHz, $CDCl_3$): δ = 143.7 (C), 131.6 (CH), 129.3 (CH), 123.1 (C), 115.3 (CH), 108.3 (C), 47.1 (CH), 29.6 (CH_2), 26.4 (CH_2), 22.4 (CH_3) ppm.

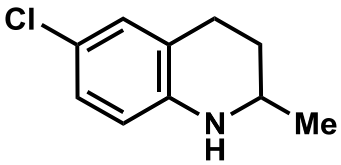

6-chloro-2-methyl-1,2,3,4-tetrahydroquinoline (5d)
Synthesized according to GP1 and characterized according to NMR comparison.[9]
^{1}H NMR (400 MHz, $CDCl_3$): δ = 6.98-6.86 (m, 2H), 6.39 (d, *J* = 8.4 Hz, 1H), 3.71 (br. s., 1H), 3.39 (dqd, *J* = 9.6, 6.4, 2.8 Hz, 1H), 2.88-2.64 (m, 2H), 1.98-1.88 (m, 1H), 1.56 (dddd, *J* = 12.9, 11.4, 10.0, 5.3 Hz, 1H), 1.22 (d, *J* = 6.4 Hz, 3H) ppm; **^{13}C NMR** (101 MHz, $CDCl_3$): δ = 143.3 (C), 128.8 (CH), 126.4 (CH), 122.6 (C), 121.2 (C), 114.9 (CH), 47.1 (CH), 29.7 (CH_2), 26.4 (CH_2), 22.4 (CH_3) ppm.

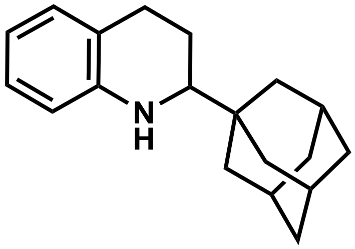

2-((1s,3s)-adamantan-1-yl)-1,2,3,4-tetrahydroquinoline (5e)
Synthesized according to GP1.
IR (neat, cm^{-1}): 3426(br), 2903(vs), 2847(m), 1482(m), 744(m);**^{1}H NMR** (400 MHz, $CDCl_3$): δ = 6.97-6.87 (m, 2H), 6.55 (td, *J* = 7.3, 1.0 Hz, 1H), 6.47 (d, *J* = 7.9 Hz, 1H), 3.83 (br. s., 1H), 2.83-2.66 (m, 3H), 2.01 (m, 3H), 1.97-1.92 (m, 1H), 1.76-1.54 (m, 13H) ppm; **^{13}C NMR** (101 MHz, $CDCl_3$): δ = 145.5 (C), 128.9 (CH), 126.7 (CH), 121.6 (C), 116.6 (CH), 114.0 (CH), 61.1 (CH), 38.3 (3 X CH_2), 37.3 (3 X CH_2), 35.2 (C), 28.5 (3 X CH), 27.4 (CH_2), 21.7 (CH_2) ppm; **HRMS** (EI): m/z calc'd for $C_{19}H_{25}N$ [M^+] 267.1987, found 267.2019.

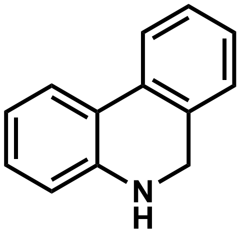

5,6-dihydrophenanthridine (5f)
Synthesized according to GP1 and characterized according to NMR comparison.[7]
^{1}H NMR (400 MHz, $CDCl_3$): δ = 7.75-7.64 (m, 2H), 7.32 (td, *J* = 7.6, 1.4 Hz, 1H), 7.22 (td, *J* = 7.4, 1.3 Hz, 1H), 7.16-7.06 (m, 2H), 6.85 (td, *J* = 7.5, 1.2 Hz, 1H), 6.68 (dd, *J* = 7.9, 1.0 Hz, 1H), 4.41 (s, 2H), 3.99 (br. s., 1H) ppm; **^{13}C NMR** (101 MHz, $CDCl_3$): δ = 145.7 (C), 132.7 (C), 132.1 (C), 128.8 (CH), 127.6 (CH), 127.1 (CH), 126.0 (CH), 123.6 (CH), 122.4 (CH), 122.1 (C), 119.3 (CH), 115.1 (CH), 46.4 (CH_2) ppm.

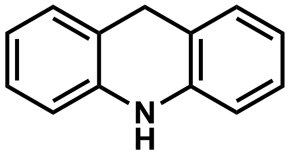

9,10-dihydroacridine (5g)
Synthesized according to GP1 and characterized according to NMR comparison.[7]
^{1}H NMR (400 MHz, $CDCl_3$): δ = 7.15-7.06 (m, 4H), 6.88 (td, *J* = 7.4, 1.1 Hz, 2H), 6.68 (dd, *J* = 7.9, 0.8 Hz, 2H), 5.96 (br. s., 1H), 4.08 (s, 2H) ppm; **^{13}C NMR** (101 MHz, $CDCl_3$): δ = 140.1 (2 X C), 128.6 (2 X CH), 127.0 (2 X CH), 120.6 (2 X CH), 120.0 (2 X C), 113.4 (2 X CH), 31.4 (CH_2) ppm.

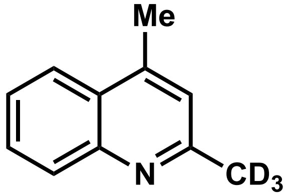

2-[$^2H_3$]-methyl-4-methylquinoline (d_3-2a)
Synthesized according to GP1.
IR (neat, cm^{-1}): 3061(m), 2923(m), 1603(s), 1561(m), 756(vs); **^{1}H NMR** (600 MHz, $CDCl_3$): δ = 8.02 (dd, *J* = 8.4, 0.6 Hz, 1H), 7.94 (dd, *J* = 8.3, 1.1 Hz, 1H), 7.67 (ddd, *J* = 8.3, 6.9, 1.5 Hz, 1H), 7.50 (ddd, *J* = 8.3, 6.9, 1.2 Hz, 1H), 7.13 (d, *J* = 0.9 Hz, 1H), 2.66 (d, *J* = 0.9 Hz, 3H) ppm; **^{13}C NMR** (151 MHz, $CDCl_3$): δ = 158.6 (C), 147.7 (C), 144.1 (C), 129.1 (CH), 129.1 (CH), 126.5 (C), 125.4 (CH), 123.5 (CH), 122.7 (CH), 24.4 (spt, *J* = 19.6 Hz, CD_3), 18.5 (CH_3) ppm (CD_3 observed in spectrum when using 30° pulse and 30 second interpulse delay); **HRMS** (EI): m/z calc'd for $C_{11}H_9D_2N$ [M^+] 160.1080, found 160.1087.

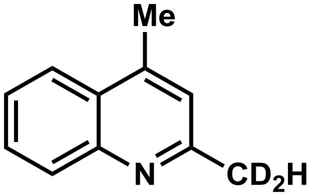

2-[$^2H_2$]-methyl-4-methylquinoline (d_2-2a)
Synthesized according to GP1.
IR (neat, cm^{-1}): 3062(m), 2924(m), 1603(s), 1561(m), 756(vs); **^{1}H NMR** (400 MHz, $CDCl_3$): δ = 8.02 (dd, *J* = 8.4, 0.6 Hz, 1H), 7.94 (d, *J* = 8.4 Hz, 1H), 7.67 (ddd, *J* = 8.2, 7.1, 1.2 Hz, 1H), 7.50 (ddd, *J* = 8.2, 7.1, 1.2 Hz, 1H), 7.13 (s, 1H), 2.66 (s, 4H) ppm; **^{13}C NMR** (101 MHz, $CDCl_3$): δ = 158.6 (C), 147.7 (C), 144.1 (C), 129.1 (CH), 129.0 (CH), 126.5 (C), 125.4 (CH), 123.5 (CH), 122.7 (CH), 24.7 (quin, *J* = 19.5 Hz, CD_2H), 18.5 (CH_3) ppm; **HRMS** (EI): m/z calc'd for $C_{11}H_9D_2N$ [M^+] 159.1017, found 159.1024.

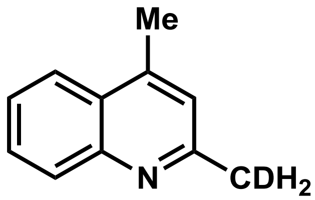

2-[^{2}H]-methyl-4-methylquinoline (d-2a)

Synthesized according to GP1.

IR (neat, cm^{-1}): 3060(m), 2923(m), 1602(s), 1561(m), 756(vs); **^{1}H NMR** (400 MHz, $CDCl_3$): δ = 8.03 (dd, *J* = 8.4, 0.6 Hz, 1H), 7.94 (d, *J* = 8.4 Hz, 1H), 7.67 (ddd, *J* = 8.3, 7.0, 1.2 Hz, 1H), 7.50 (ddd, *J* = 8.2, 7.1, 1.0 Hz, 1H), 7.13 (s, 1H), 2.69-2.67 (m, 2H), 2.66 (s, 3H) ppm; **^{13}C NMR** (101 MHz, $CDCl_3$): δ = 158.6 (C), 147.7 (C), 144.1 (C), 129.1 (CH), 129.0 (CH), 126.5 (C), 125.4 (CH), 123.5 (CH), 122.7 (CH), 24.9 (t, *J* = 19.5 Hz, CDH_2), 18.5 (CH_3) ppm; **HRMS** (EI): m/z calc'd for $C_{11}H_{10}DN$ [M^+] 158.0954, found 158.0984.

4. Kinetic Isotope Effect Experiments

For kinetic isotope effect experiments (KIE) using MeOH, THF, and iPrOH, the reactions were prepared in the usual way using a 1:1 mixture of protonated and deuterated solvent, where KIE data was obtained by analyzing the ratio of protonated and deuterated products observed.

For the KIE study conducted using **1a** and **d-1a**, the synthesis of **d-1a** was achieved as follows:

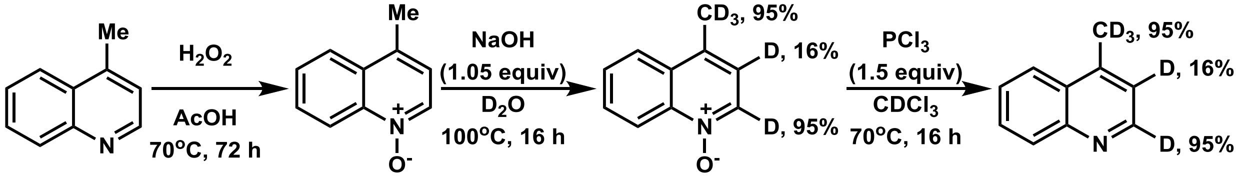

The corresponding lepidine N-oxide was formed using literature procedure.[10] The resulting compound was treated under basic conditions (NaOH, 1.05 equiv) in D_2O following literature procedure.[11] The resulting product was then reduced using PCl_3 following literature procedures,[12] affording the corresponding deuterated lepidine **d-1a** (95% D at C2, 16% D at C3, 95% CD_3 at Me, shown in top spectrum of Figure S1). This compound was partitioned (51:49, **1a**:**d-1a**, middle spectrum of Figure S1) and the optimized reaction conditions were applied. When the reaction was not complete (~70% conversion) the reaction was stopped and the ratio of starting materials remaining were analyzed. It was found that the starting material remaining was a 37:63 mixture of **1a**:**d-1a**, indicating that **1a** reacted faster than its deuterated counterpart, and can be calculated by comparison of ratio of starting materials converted to product followed by accounting for initial ratio, giving a KIE of 1.77, bottom spectrum of Figure S1, (63/37)x(51/49).

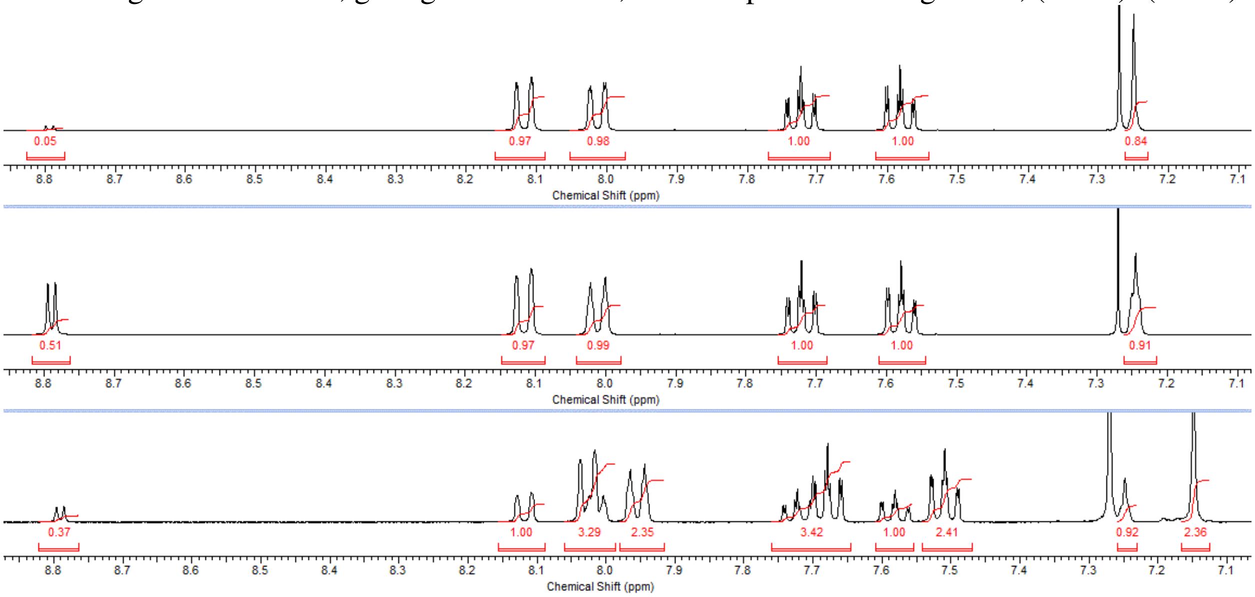

Figure S1. KIE study between **1a**:**d-1a** under optimized conditions.

5. Steady-State Fluorescence Quenching of Lepidine

The fluorescence emission measurements required for the lepidine singlet quenching experiments were carried out in a Photon Technology International (PTI) spectrofluorimeter at room temperature using 1 × 1 cm^2 quartz cuvettes. Samples of lepidine were prepared with a final absorbance of 0.3 at 340 nm, the wavelength employed for excitation, in 2 M HCl in MeCN or H_2O. The MeOH and THF quencher solutions used in the quenching studies were also prepared with HCl (2 M) and with lepidine with an absorbance of 0.3 at 340 nm to ensure that any observed quenching was not due to the dilution of lepidine.

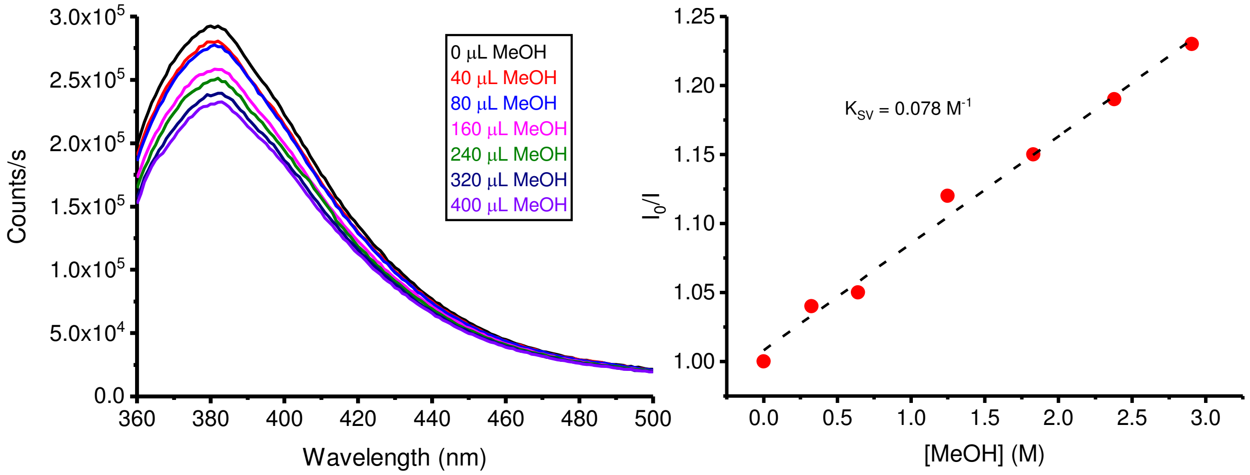

Figure S2. Data for the steady-state quenching experiments of lepidine by MeOH in 2 M HCl in H_2O. **(Left)** Quenching of the emission of lepidine upon increasing concentrations of MeOH. **(Right)** Corresponding Stern-Volmer plot.

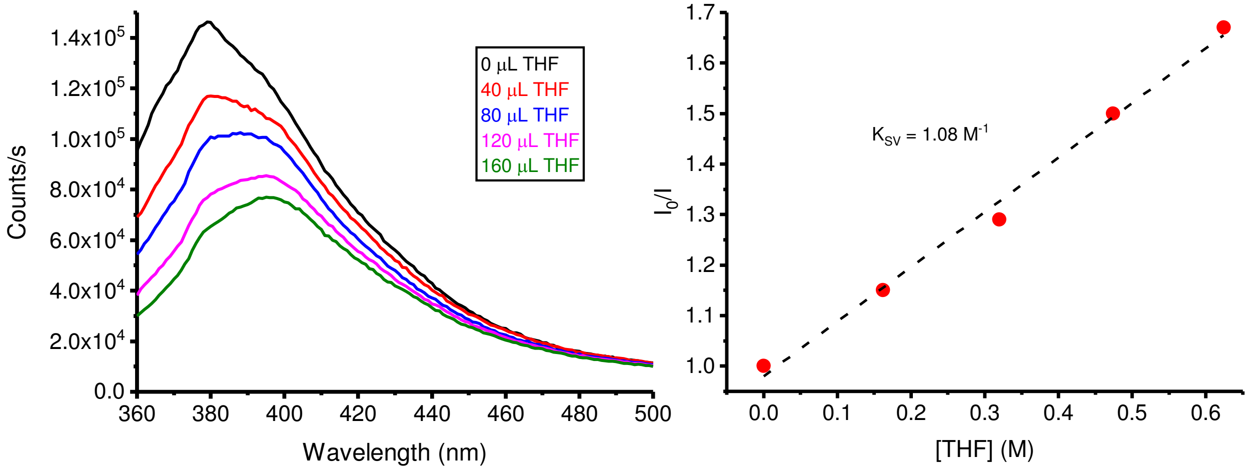

Figure S3. Data for the steady-state quenching experiments of lepidine by THF in 2 M HCl in MeCN. **(Left)** Quenching of the emission of lepidine upon increasing concentrations of THF. **(Right)** Corresponding Stern-Volmer plot.

6. Intermittent Illumination Experimental

Typically, lepidine (0.4 mmol, 1.0 equiv, 57 mg), MeOH (0.8 mL) and then concentrated HCl (2.0 mmol, 5.0 equiv, 150 μL) was added to a 3 mL quartz cuvette fitted with a septum. The reaction mixture was then degassed with argon for 15 minutes before it was intermittently irradiated at 70 °C for 30 minutes (total light on time) using a pulsed 365 nm LED, which was powered by a constant current driver (designed and built in house) and controlled by a digital delay/pulse generator (Stanford Research System Inc.- MODEL DG535). In all cases the system was interfaced with an oscilloscope (Tektronix–MODEL TDS3052), which monitored the delivered voltage and resulting current of the system. The system was also interfaced with a photodiode, which allowed the shape and duration of the light pulse emitted from the LED to be monitored. This also allowed for real time monitoring of the light pulse to ensure that the appropriate light on:light off ratio was being employed. A light on:light off ratio of 1:2 was used in all trials, and the length of the on and off times were increased proportionally with each successive trial. After irradiation, the solution was poured into a separatory funnel with 1 M NaOH and extracted with DCM. The combined organic extracts were dried over Na_2SO_4, filtered, and concentrated. Yield were determined by ^{1}H NMR using mesitylene as an external standard.

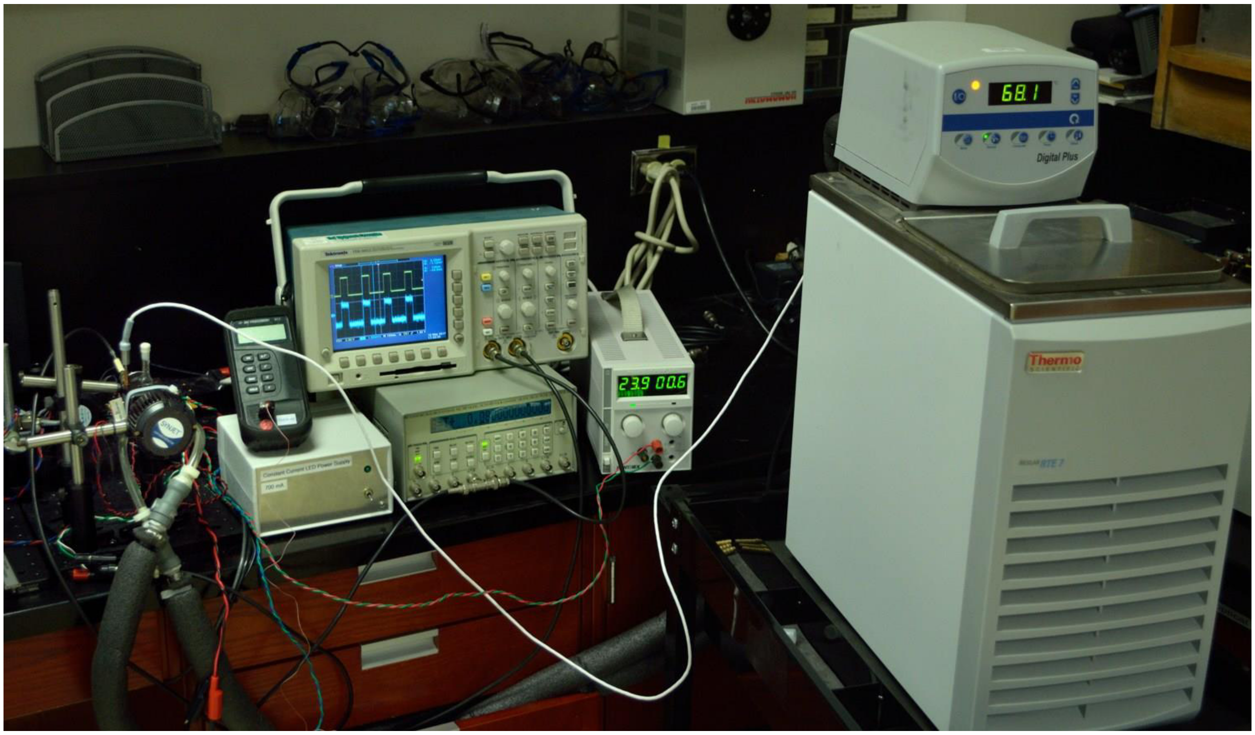

Figure S4. Photograph of the experimental set-up employed for the intermittent illumination experiments.

7. References

[1] S. Naidoo, V. Jeena, *Heterocycles,* 2016, **92**, 1664.
[2] J. Jin, D. W. C. MacMillan, *Nature,* 2015, **525**, 87.
[3] Y.-F. Wang, K. K. Toh, E. P. J. Ng, S. Chiba, *J. Am. Chem. Soc.,* 2011, **133**, 6411.
[4] A. B. Bellan, O. M. Kuzmina, V. A. Vetsova, P. Knochel, *Synthesis,* 2017, **49**, 188.
[5] T.-S. Balaban, C. Uncuta, M. D. Gheorghiu, A. T. Balaban, *Tetrahedron Lett.,* 1985, **26**, 4669.
[6] J. Zhang, J. W. Canary, *Org. Lett.*, 2006, **8**, 3907.
[7] F. Chen, A.-E. Surkus, L. He, M.-M. Pohl, J. Radnik, C. Topf, K. Junge, M. Beller, *J. Am. Chem. Soc.*, 2015, **137**, 11718.
[8] Y.-T. Xia, X.-T. Sun, L. Zhang, K. Luo, L. Wu, *Chem. Eur. J.,* 2016, **22**, 17151.
[9] M. Kojima, M. Kanai, *Angew. Chem. Int. Ed.*, 2016, **55**, 12224.
[10] C. Zhu, M. Yi, D. Wei, X. Chen, Y. Wu, X. Cui, *Org. Lett.*, 2014, **16**, 1840.
[11] W. Jo, J. Kim, S. Choi, S. H. Cho, *Angew. Chem. Int. Ed.*, 2016, **55**, 9690.
[12] K. T. Sylvester, K. Wu, A. G. Doyle, *J. Am. Chem. Soc.*, 2012, **134**, 16967.

8. NMR Spectra

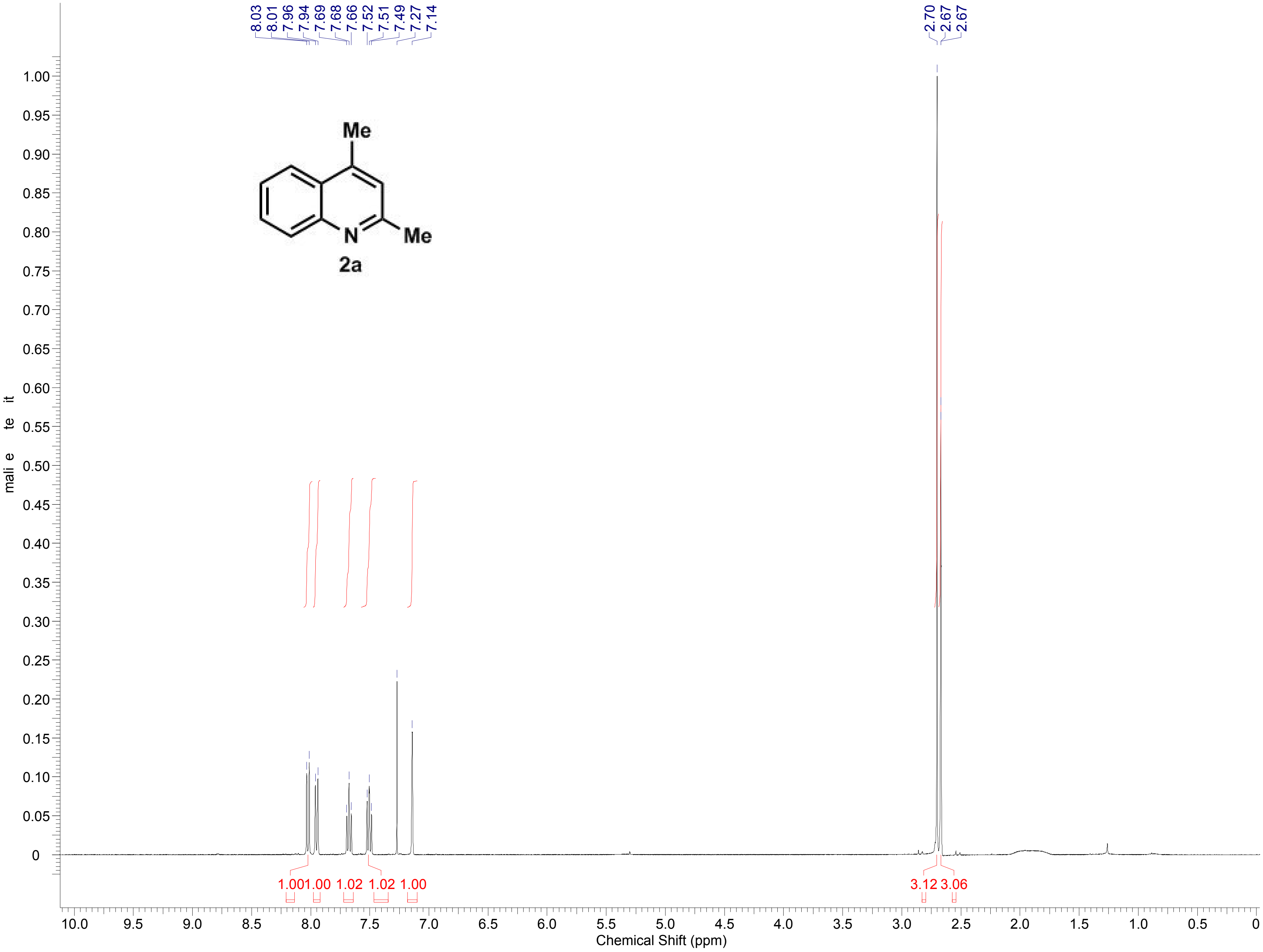

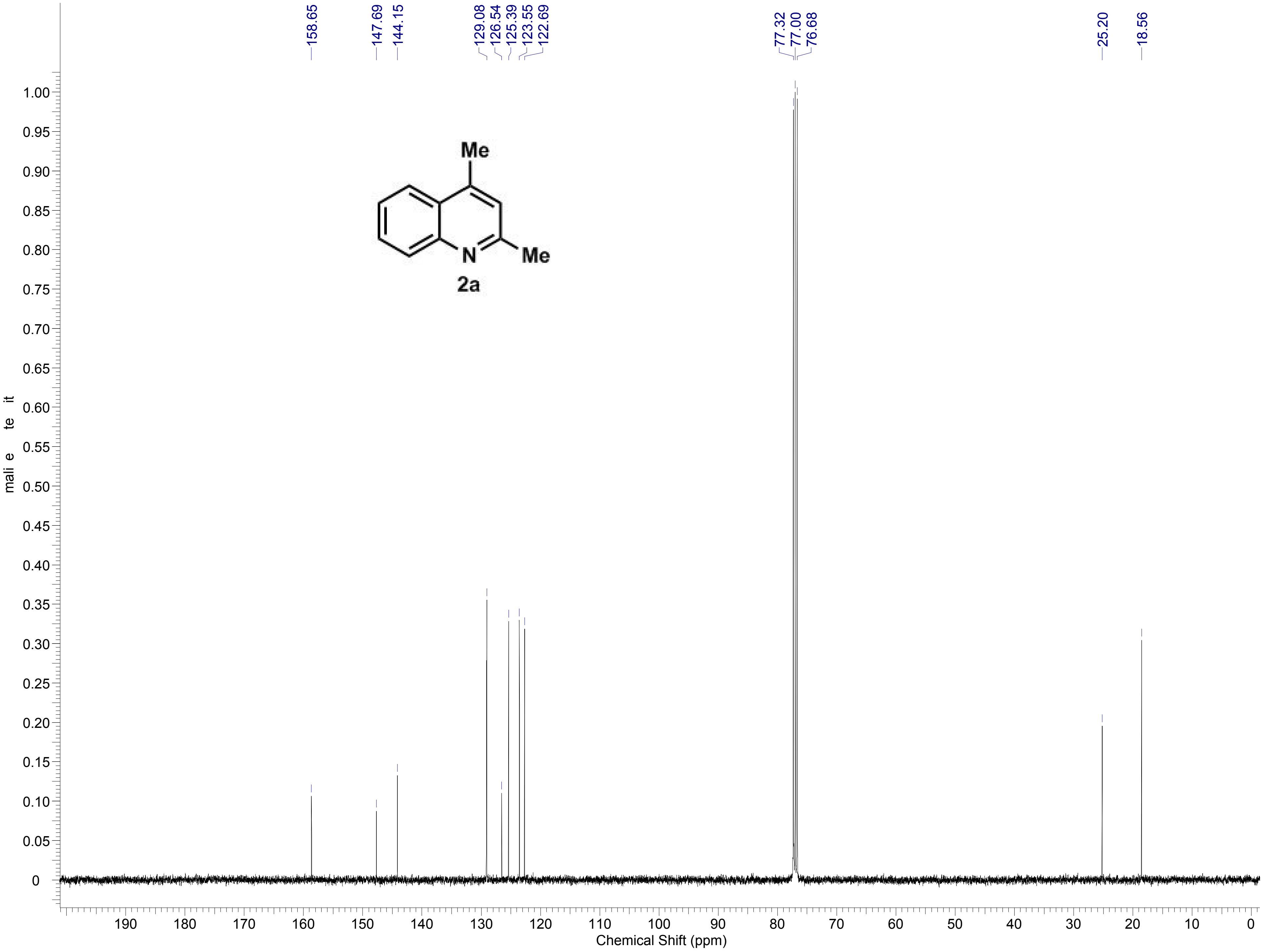
158.65
147.69
144.15
129.08
126.54
125.39
123.55
122.69
77.32
77.00
76.68
25.20
18.56
Me
N
Me
2a
Chemical Shift (ppm)

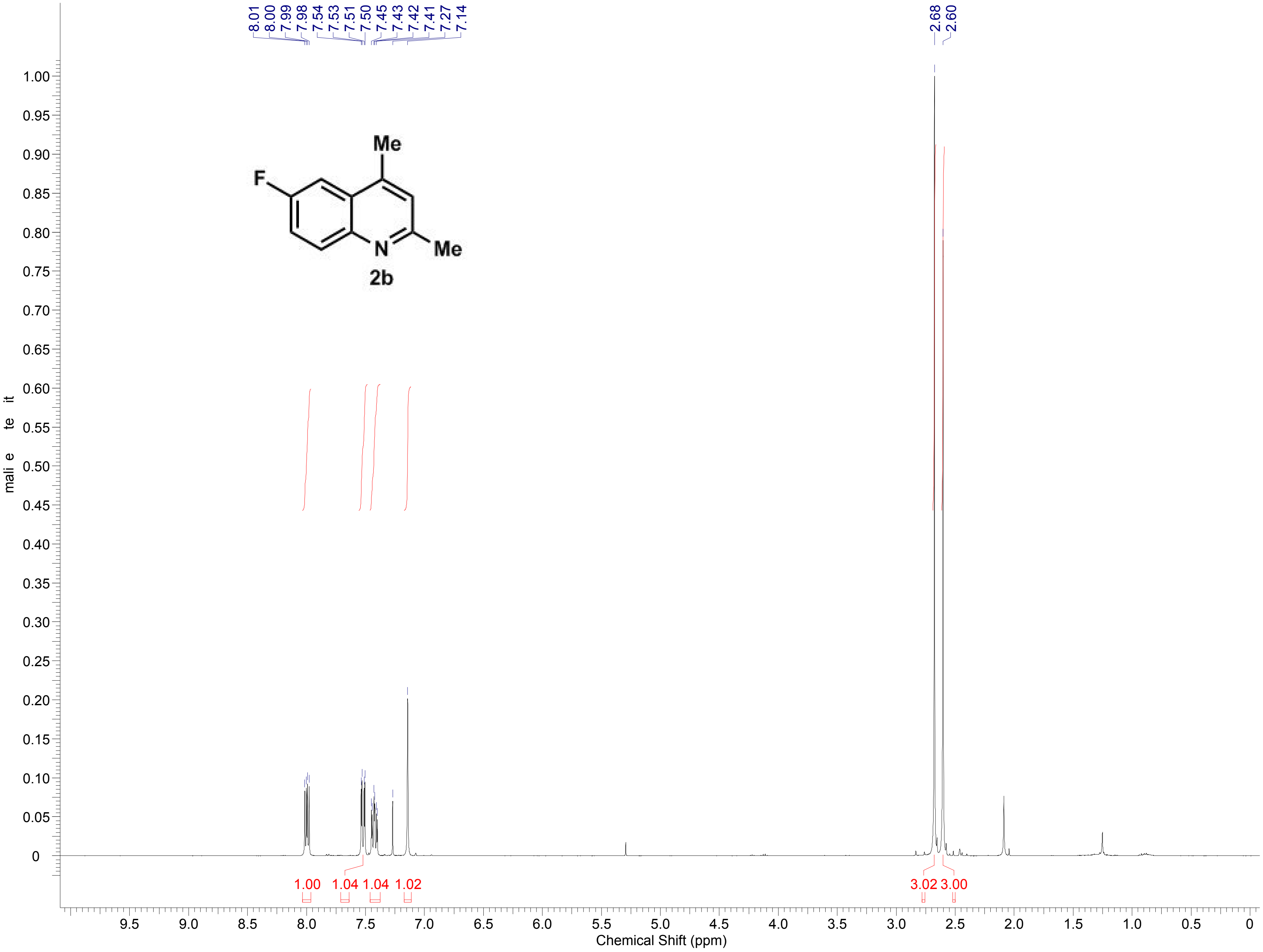
8.01
8.00
7.99
7.98
7.54
7.53
7.51
7.50
7.45
7.43
7.42
7.41
7.27
7.14
2.68
2.60
F
Me
N
Me
2b
1.00
1.04
1.04
1.02
3.02
3.00
Chemical Shift (ppm)

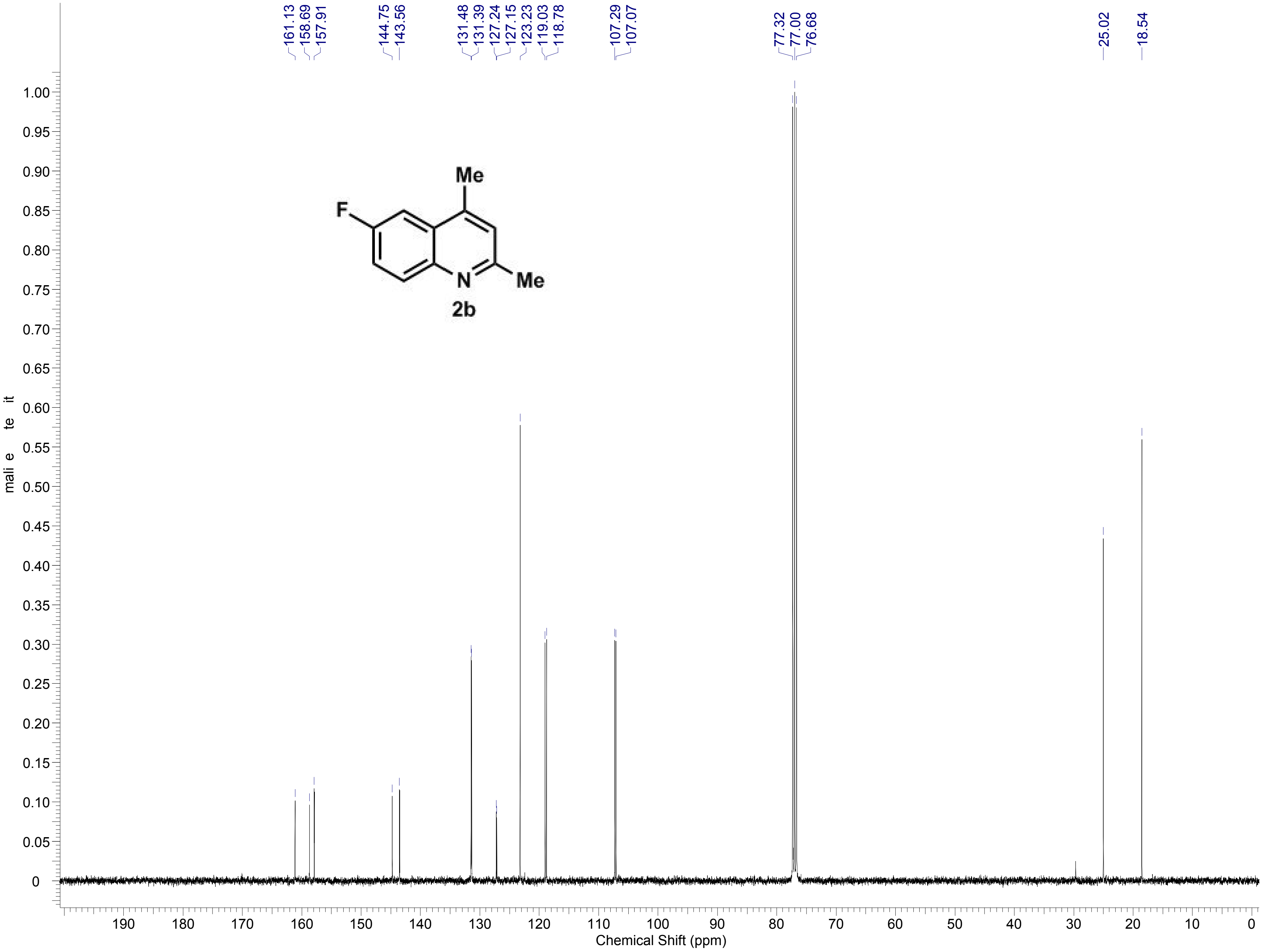
161.13
158.69
157.91
144.75
143.56
131.48
131.39
127.24
127.15
123.23
119.03
118.78
107.29
107.07
77.32
77.00
76.68
25.02
18.54
F
Me
N
Me
2b
Chemical Shift (ppm)
mali e te it

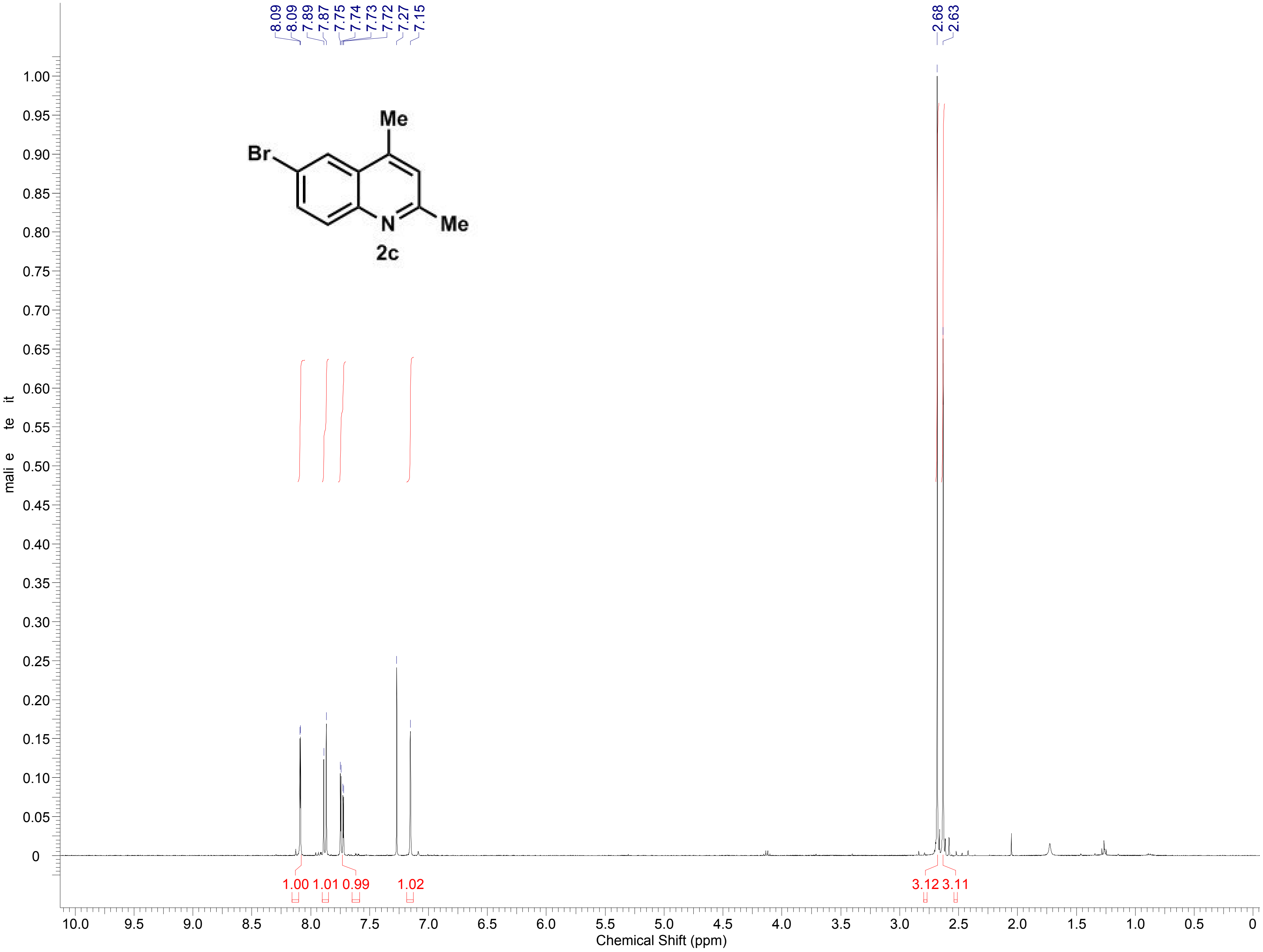
8.09
8.09
7.89
7.87
7.75
7.74
7.73
7.72
7.27
7.15
2.68
2.63
Br
Me
N
Me
2c
1.00
1.01
0.99
1.02
3.12
3.11
Chemical Shift (ppm)

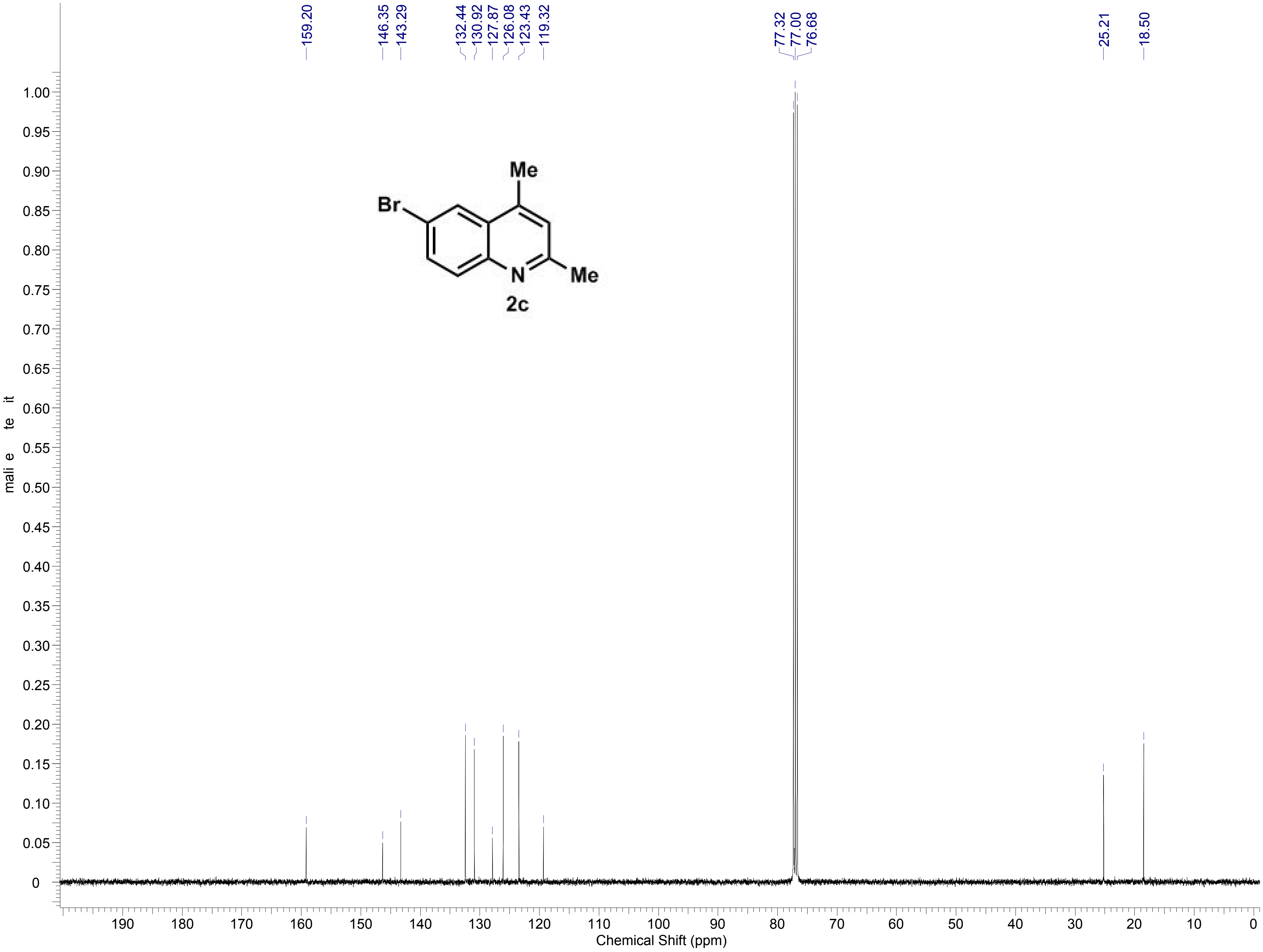
159.20
146.35
143.29
132.44
130.92
127.87
126.08
123.43
119.32
77.32
77.00
76.68
25.21
18.50
Me
Br
N
Me
2c
Chemical Shift (ppm)

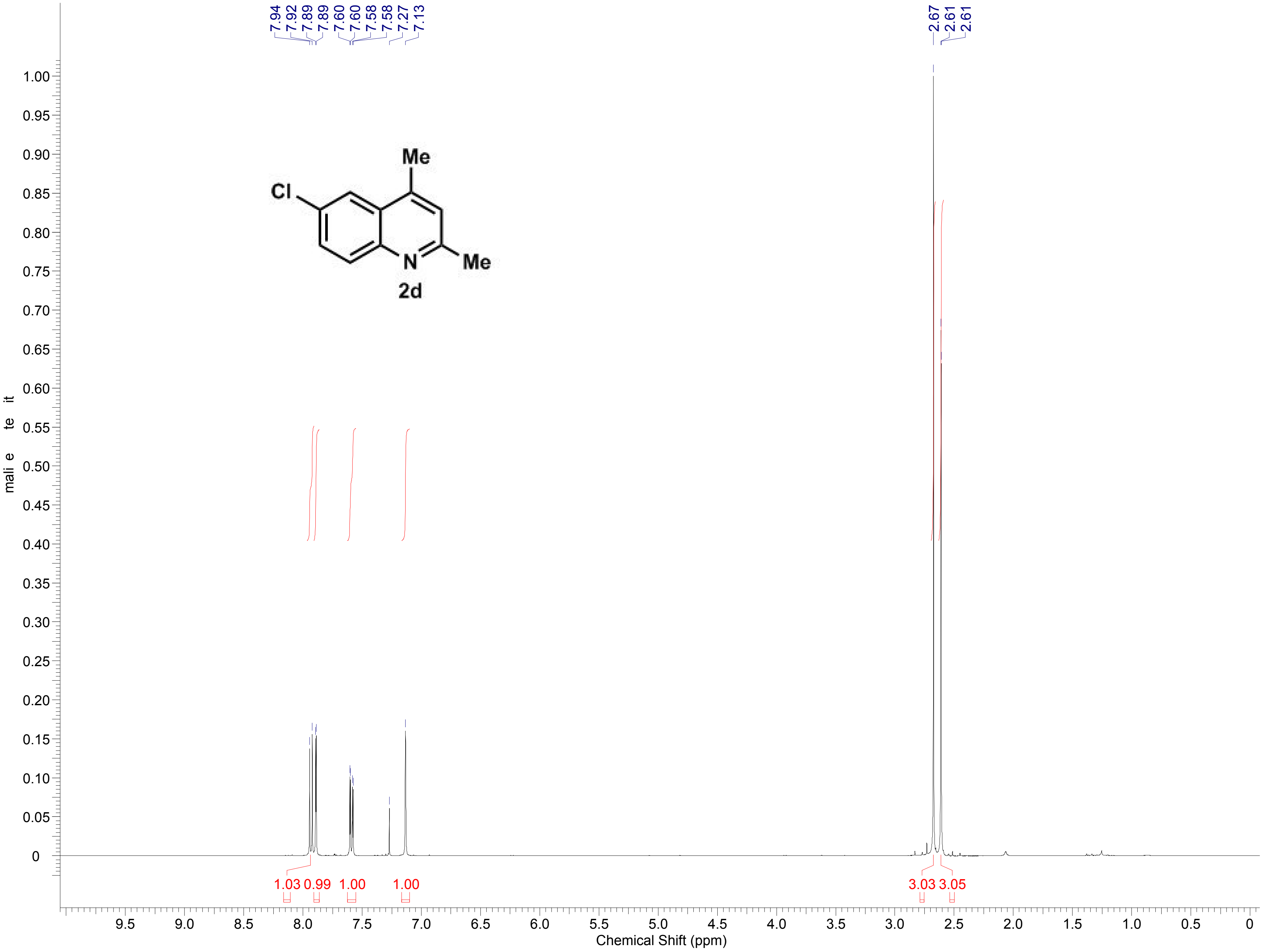
7.94
7.92
7.89
7.89
7.60
7.60
7.58
7.58
7.27
7.13
2.67
2.61
2.61
Cl
Me
N
Me
2d
1.03
0.99
1.00
1.00
3.03
3.05
Chemical Shift (ppm)

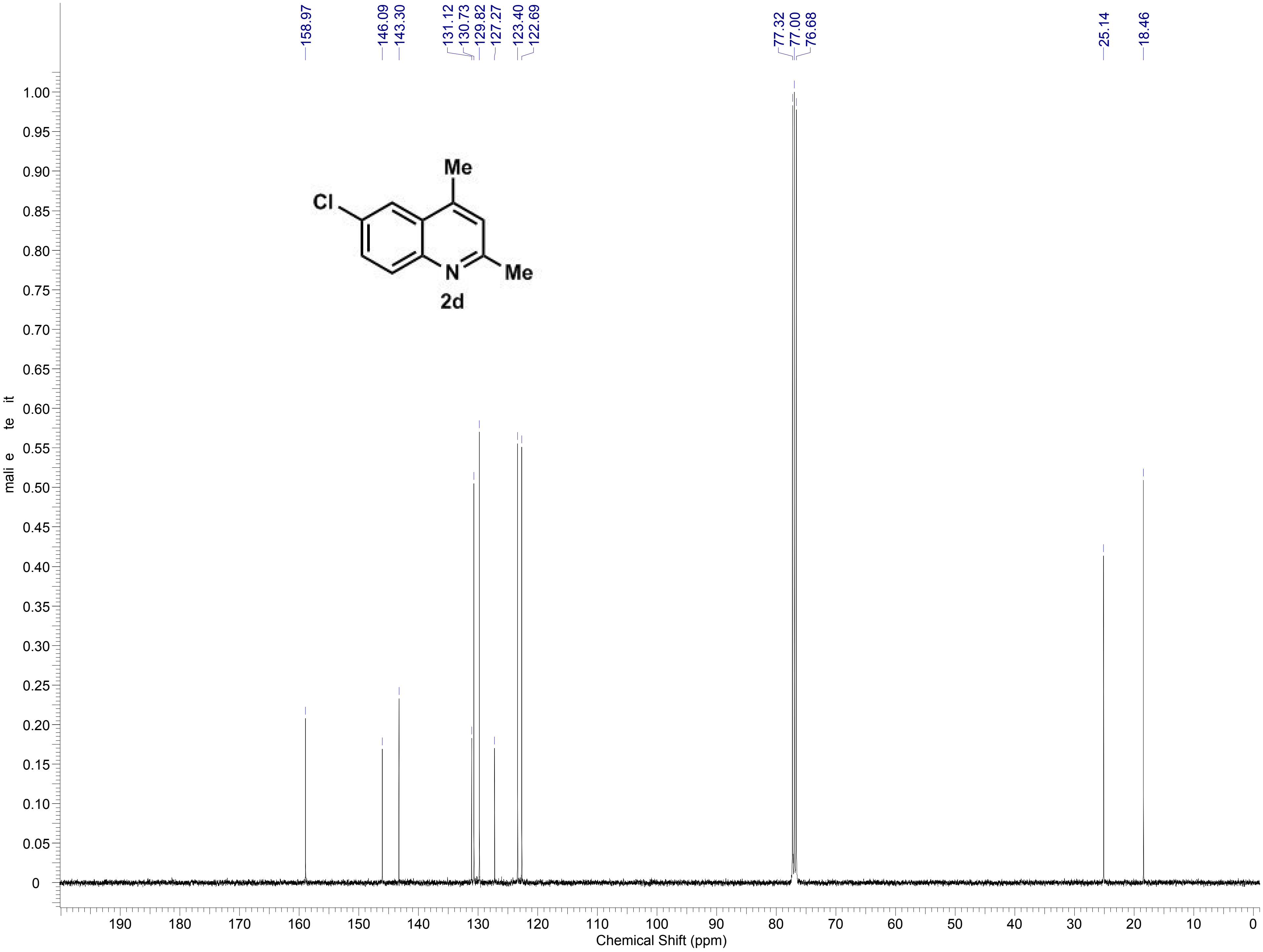
158.97
146.09
143.30
131.12
130.73
129.82
127.27
123.40
122.69
77.32
77.00
76.68
25.14
18.46
Me
Cl
N
Me
2d
Chemical Shift (ppm)

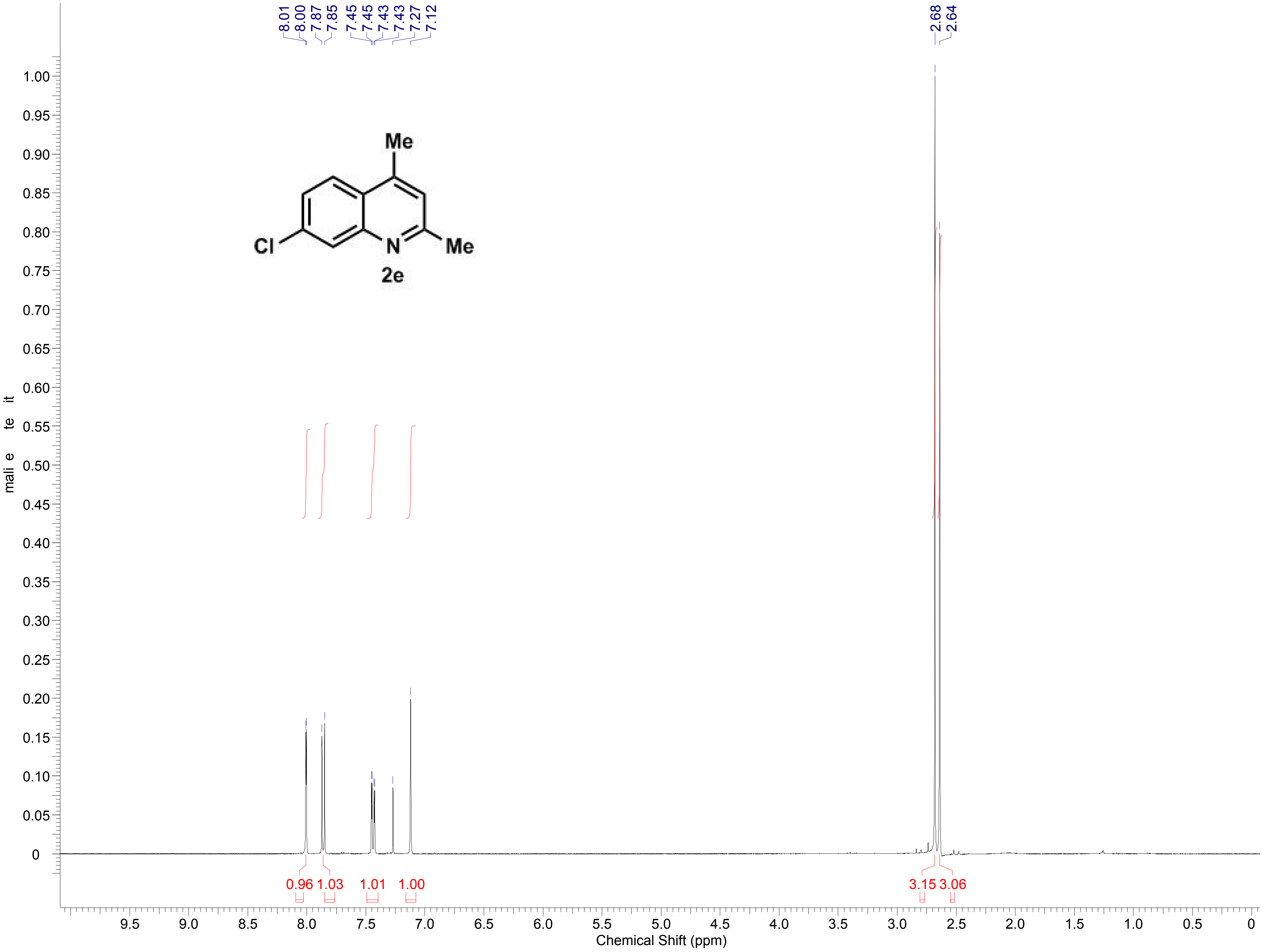
8.01
8.00
7.87
7.85
7.45
7.45
7.43
7.43
7.27
7.12
2.68
2.64
Me
Cl
N
Me
2e
0.96
1.03
1.01
1.00
3.15
3.06
Chemical Shift (ppm)

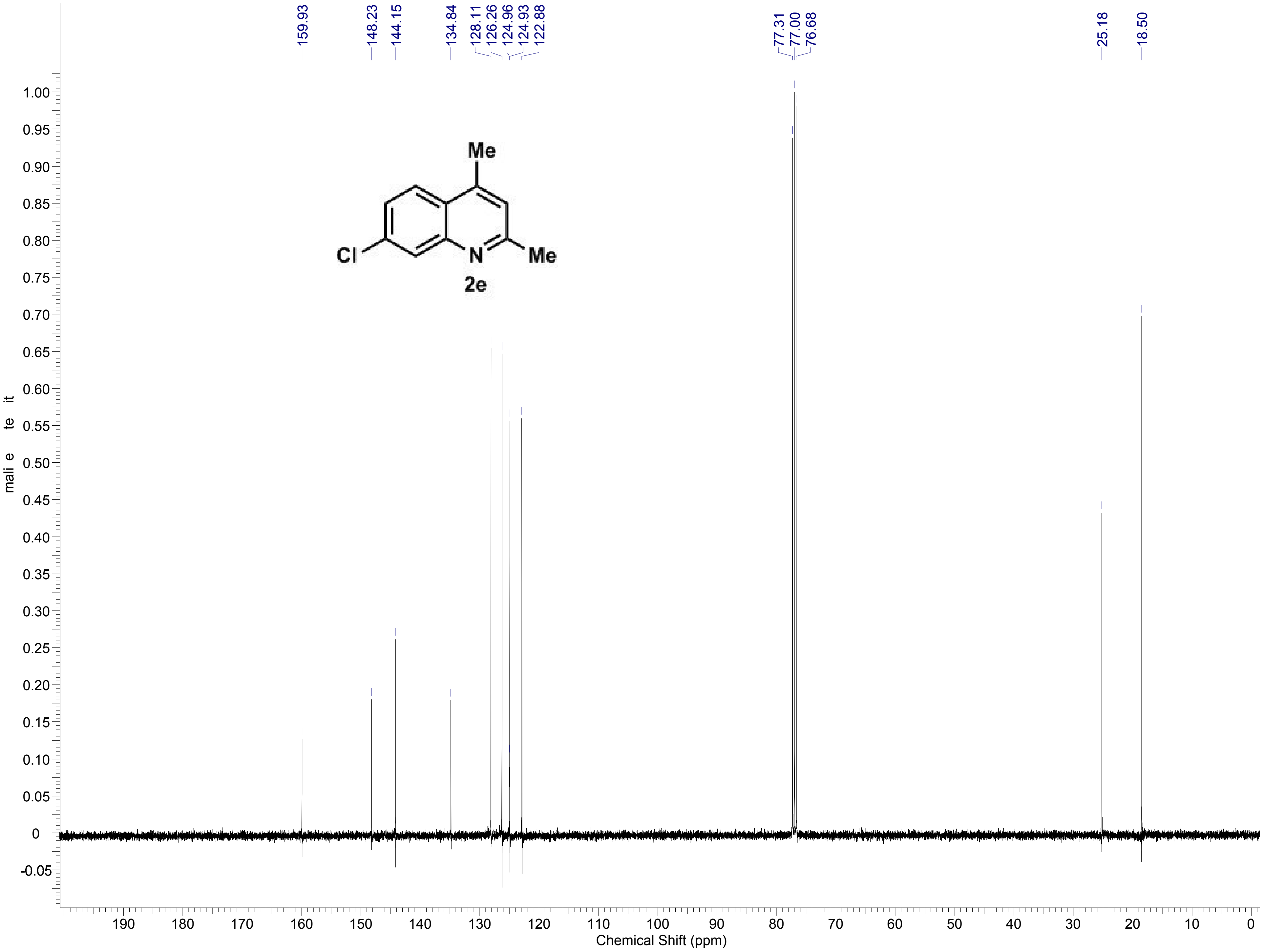

159.93
148.23
144.15
134.84
128.11
126.26
124.96
124.93
122.88
77.31
77.00
76.68
25.18
18.50
Me
Cl
N
Me
2e
Chemical Shift (ppm)

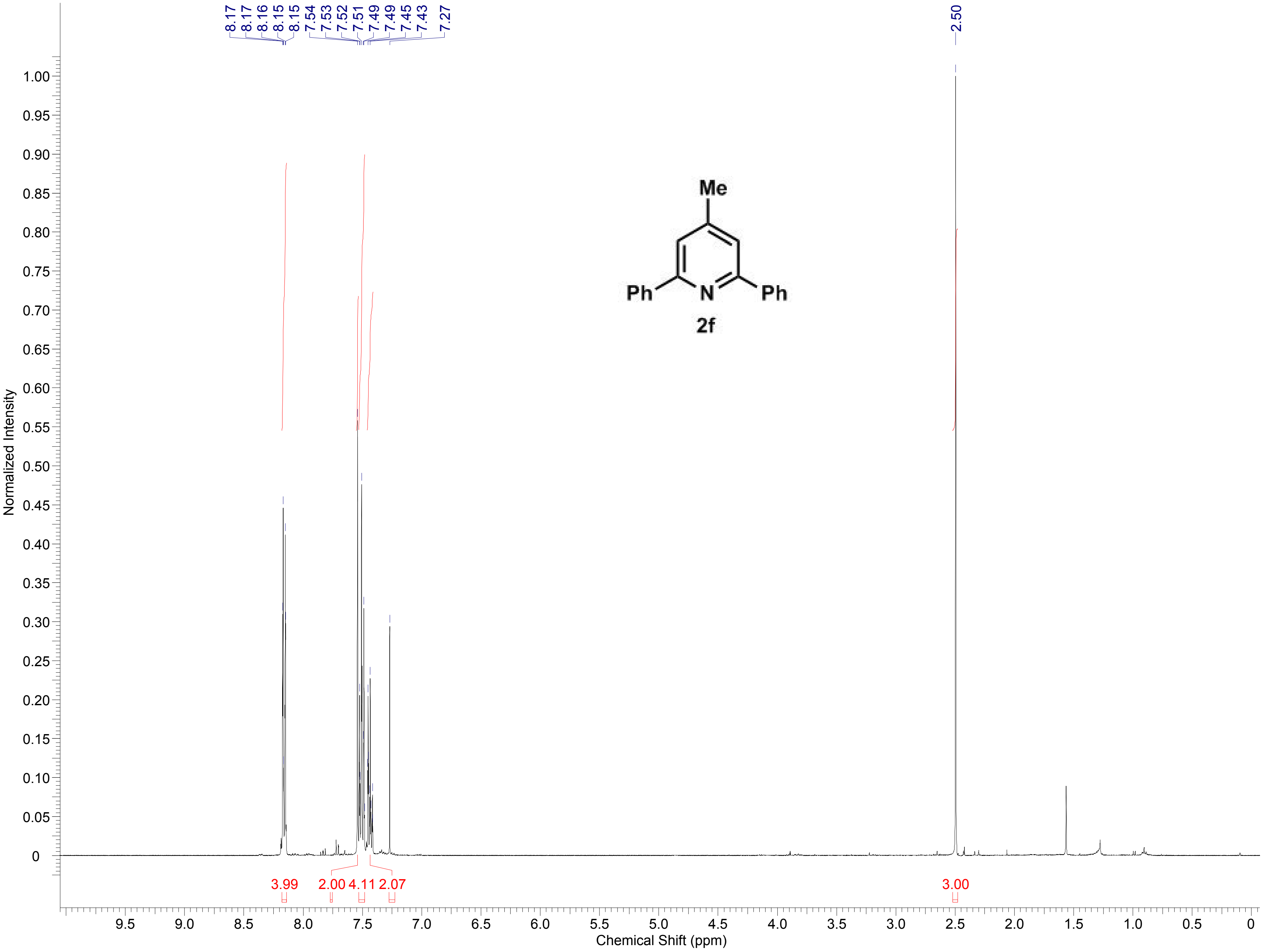
8.17
8.17
8.16
8.15
8.15
7.54
7.53
7.52
7.51
7.49
7.49
7.45
7.43
7.27
2.50
Me
Ph
N
Ph
2f
3.99
2.00
4.11
2.07
3.00
Normalized Intensity
Chemical Shift (ppm)

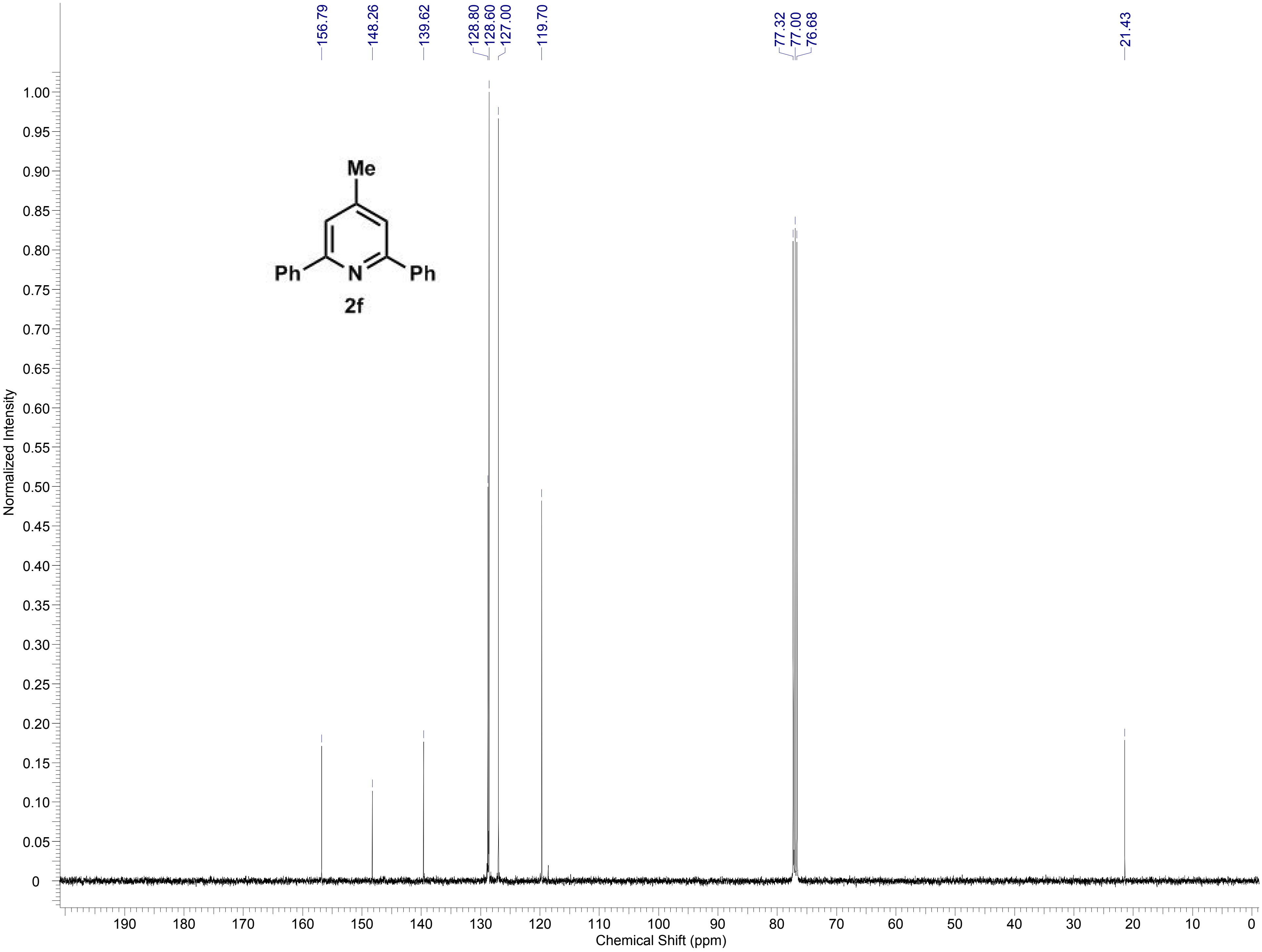

156.79
148.26
139.62
128.80
128.60
127.00
119.70
77.32
77.00
76.68
21.43
Me
Ph
N
Ph
2f
Normalized Intensity
Chemical Shift (ppm)

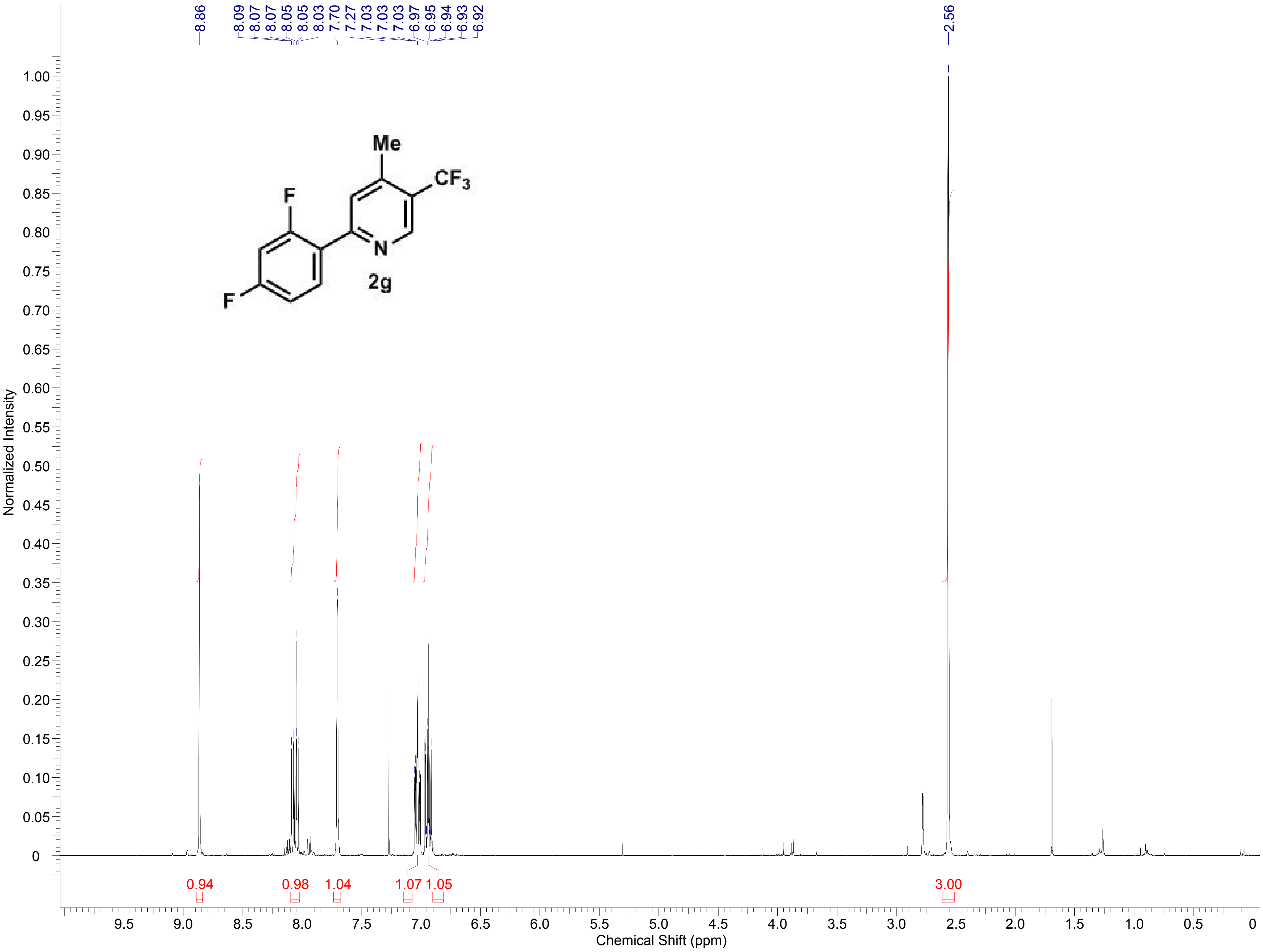
8.86
8.09
8.07
8.07
8.05
8.05
8.03
7.70
7.27
7.03
7.03
7.03
6.97
6.95
6.94
6.93
6.92
2.56
Me
CF3
F
F
N
2g
Normalized Intensity
0.94
0.98
1.04
1.07
1.05
3.00
Chemical Shift (ppm)

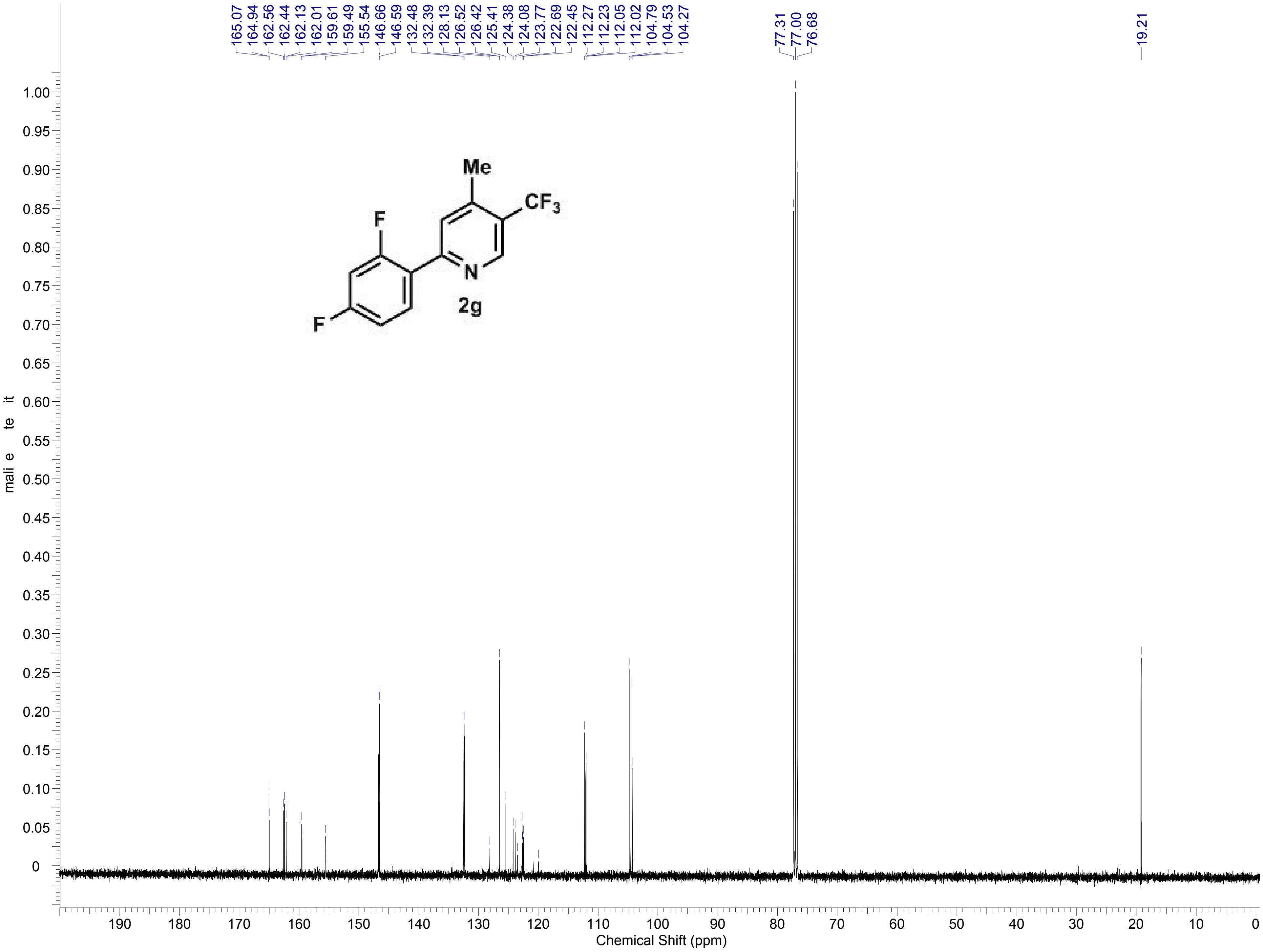
165.07
164.94
162.56
162.44
162.13
162.01
159.61
159.49
155.54
146.66
146.59
132.48
132.39
128.13
126.52
126.42
125.41
124.38
124.08
123.77
122.69
122.45
112.27
112.23
112.05
112.02
104.79
104.53
104.27
77.31
77.00
76.68
19.21
Me
CF3
F
F
N
2g
Chemical Shift (ppm)

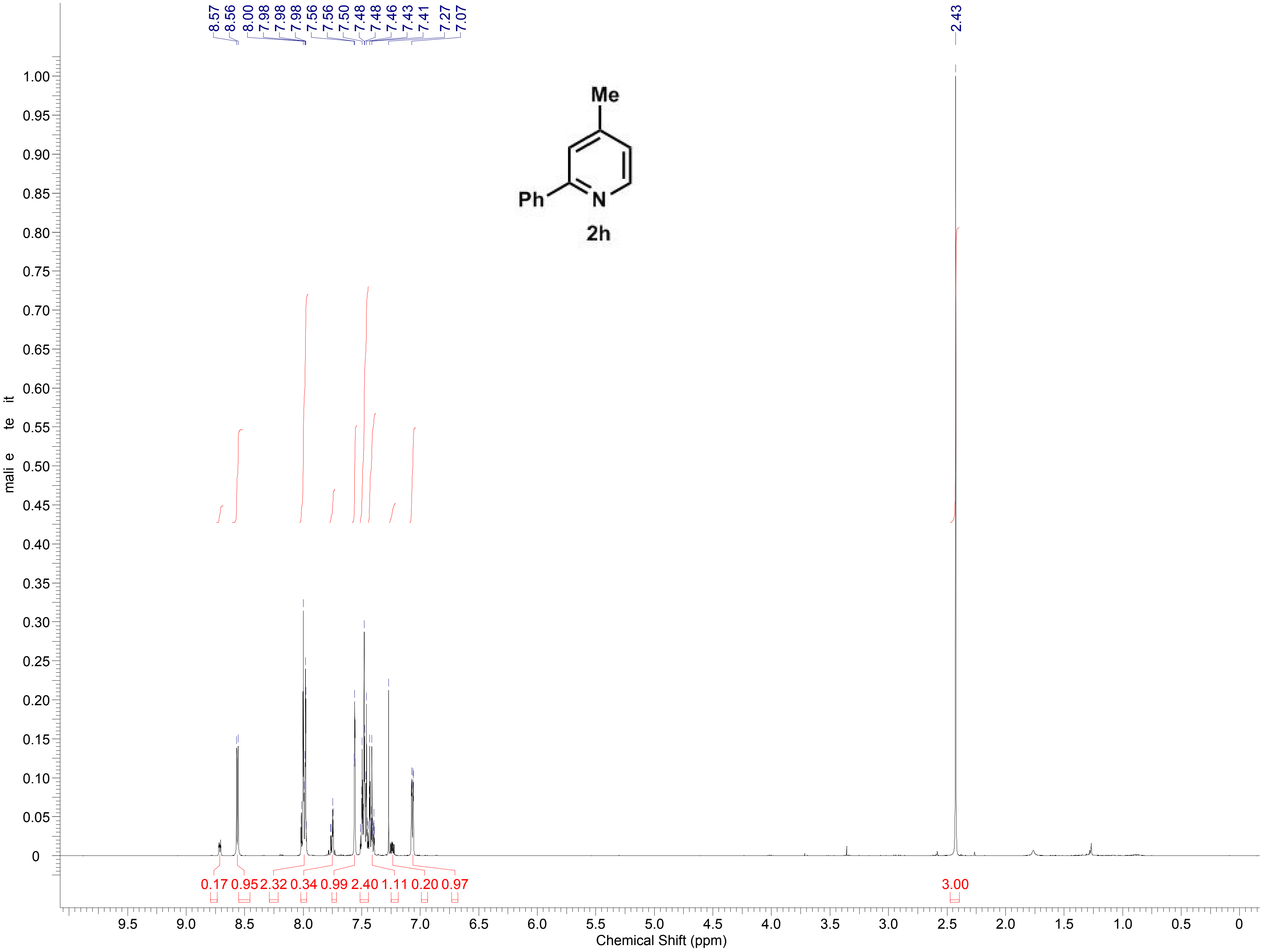
8.57 8.56 8.00 7.98 7.98 7.98 7.56 7.56 7.50 7.48 7.48 7.46 7.43 7.41 7.27 7.07
2.43
Me
Ph
N
2h
0.17 0.95 2.32 0.34 0.99 2.40 1.11 0.20 0.97
3.00
Chemical Shift (ppm)

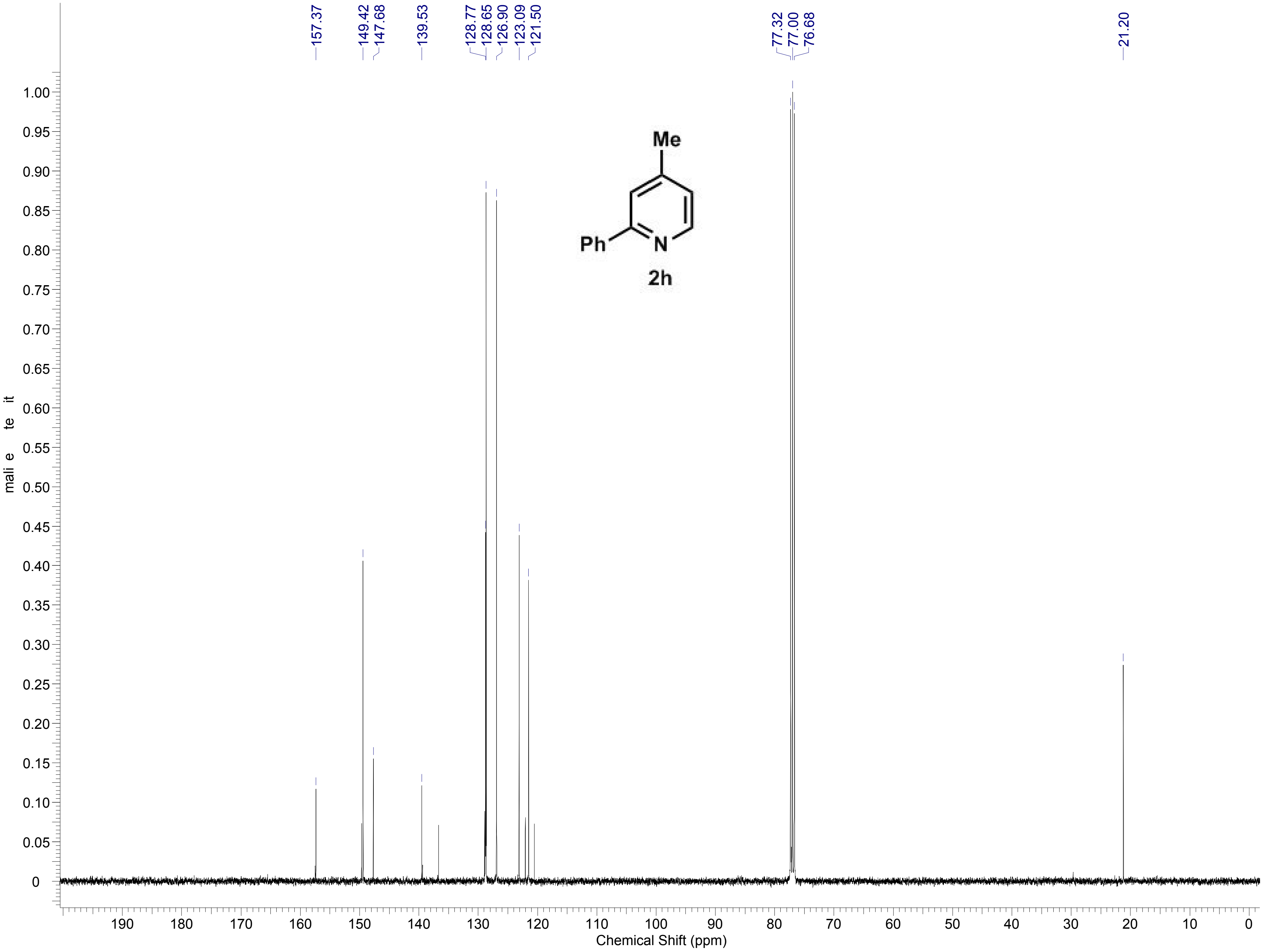
157.37
149.42
147.68
139.53
128.77
128.65
126.90
123.09
121.50
77.32
77.00
76.68
21.20
Me
Ph
N
2h
Chemical Shift (ppm)

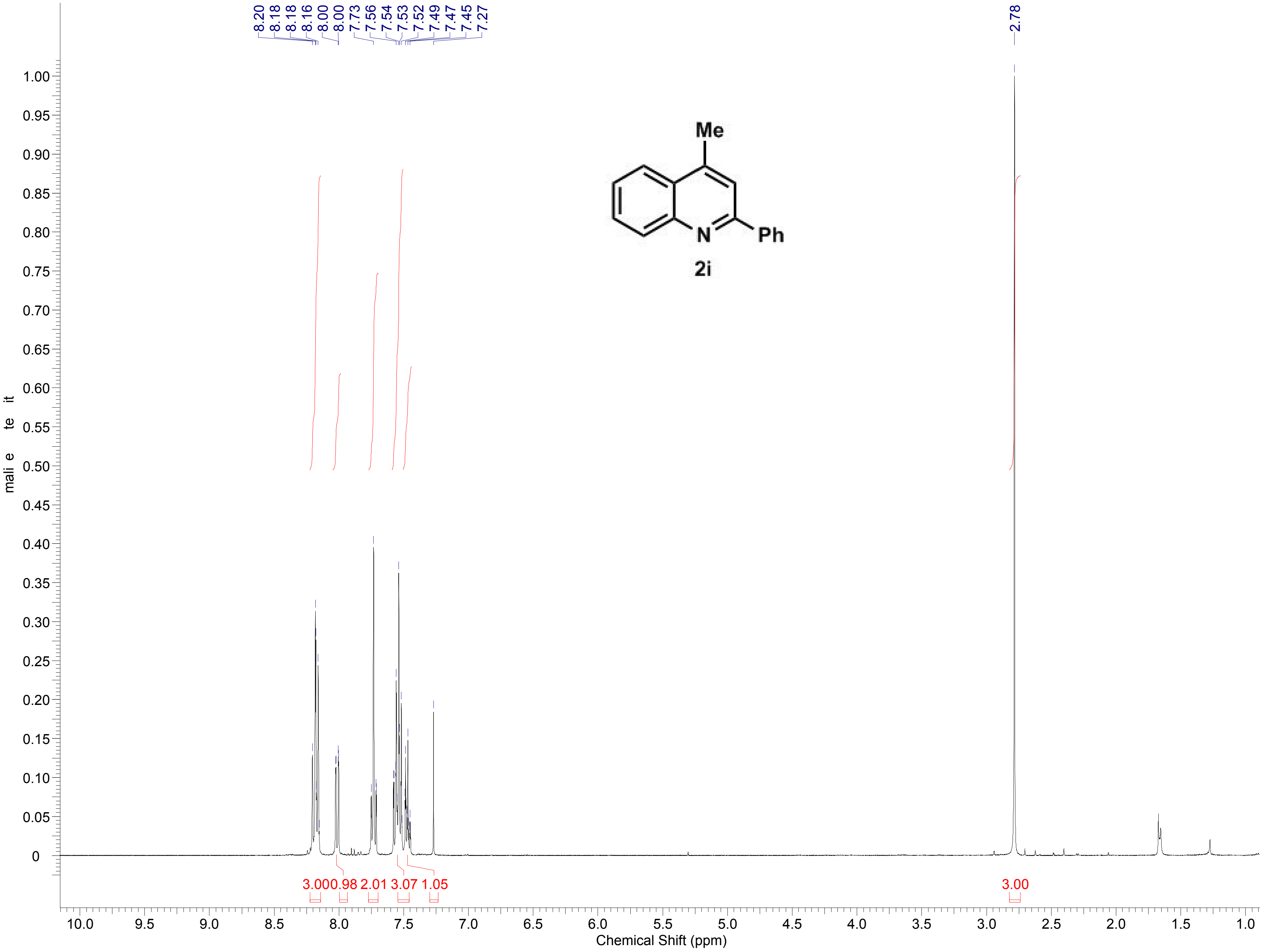
8.20
8.18
8.18
8.16
8.00
8.00
7.73
7.56
7.54
7.53
7.52
7.49
7.47
7.45
7.27
2.78
Me
N
Ph
2i
3.00
0.98
2.01
3.07
1.05
3.00
Chemical Shift (ppm)

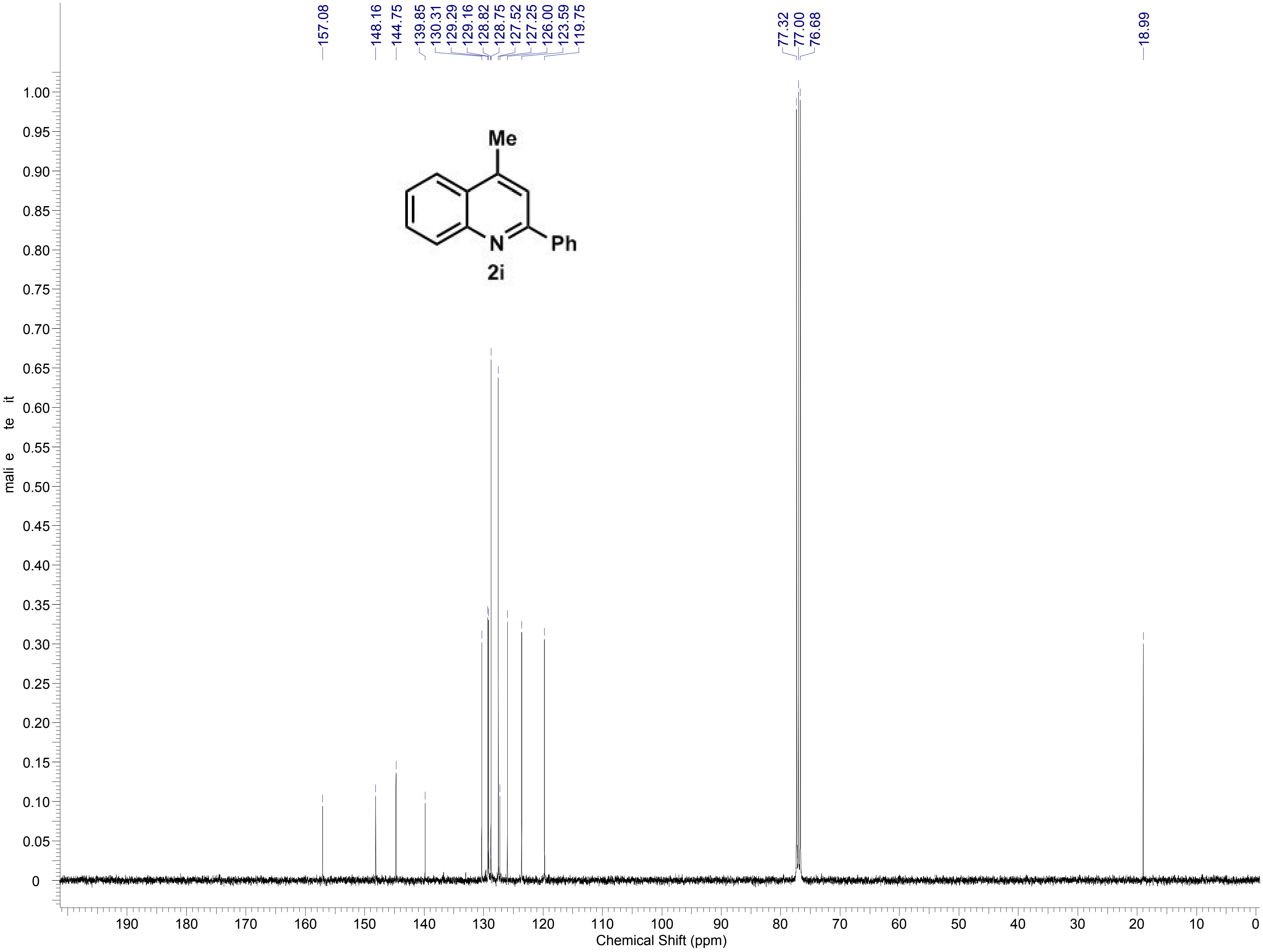

157.08
148.16
144.75
139.85
130.31
129.29
129.16
128.82
128.75
127.52
127.25
126.00
123.59
119.75
77.32
77.00
76.68
18.99
Me
N
Ph
2i
Chemical Shift (ppm)

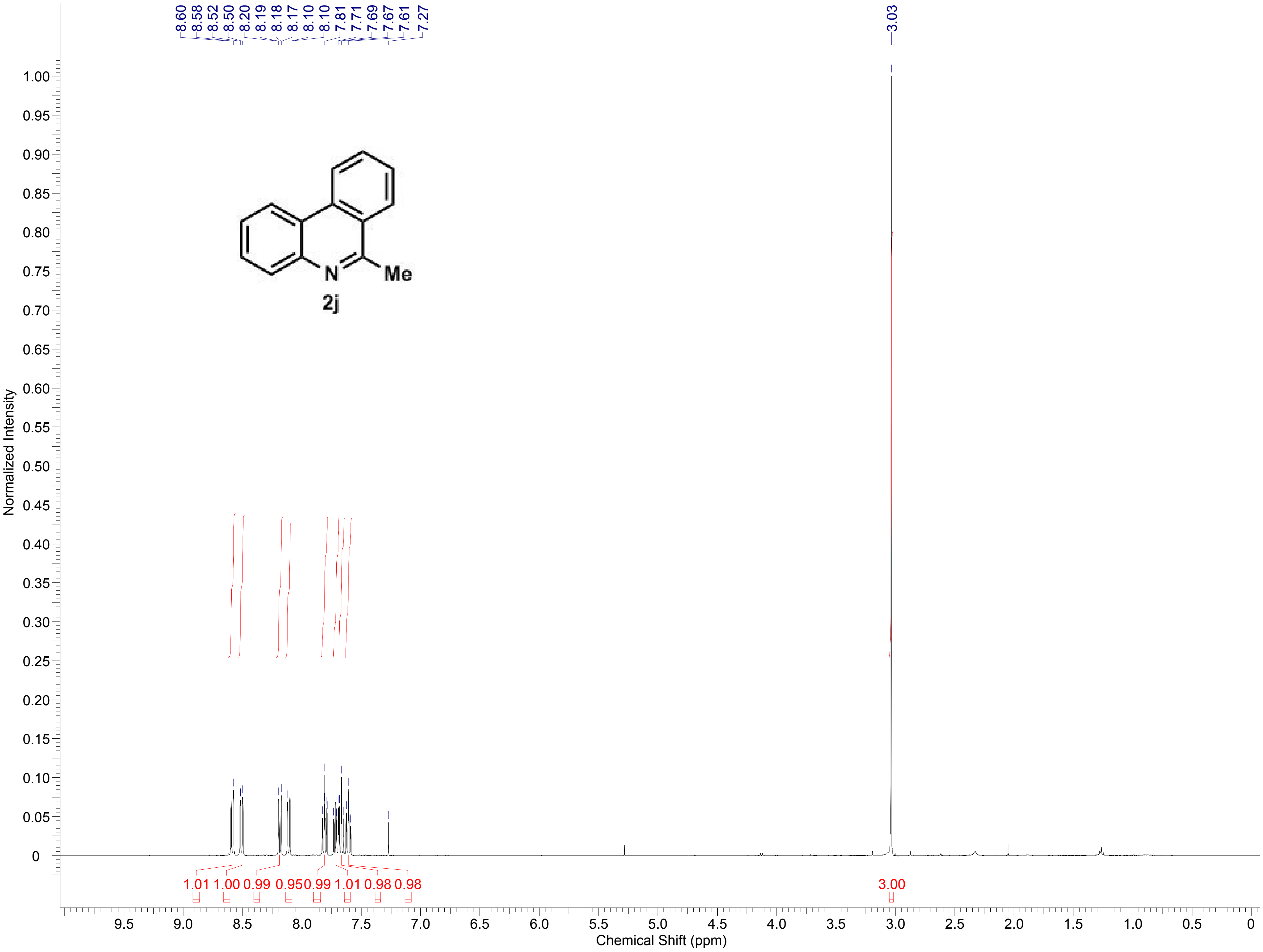
8.60 8.58 8.52 8.50 8.20 8.19 8.18 8.17 8.10 8.10 7.81 7.71 7.69 7.67 7.61 7.27
3.03
N
Me
2j
1.01 1.00 0.99 0.95 0.99 1.01 0.98 0.98
3.00
Normalized Intensity
Chemical Shift (ppm)

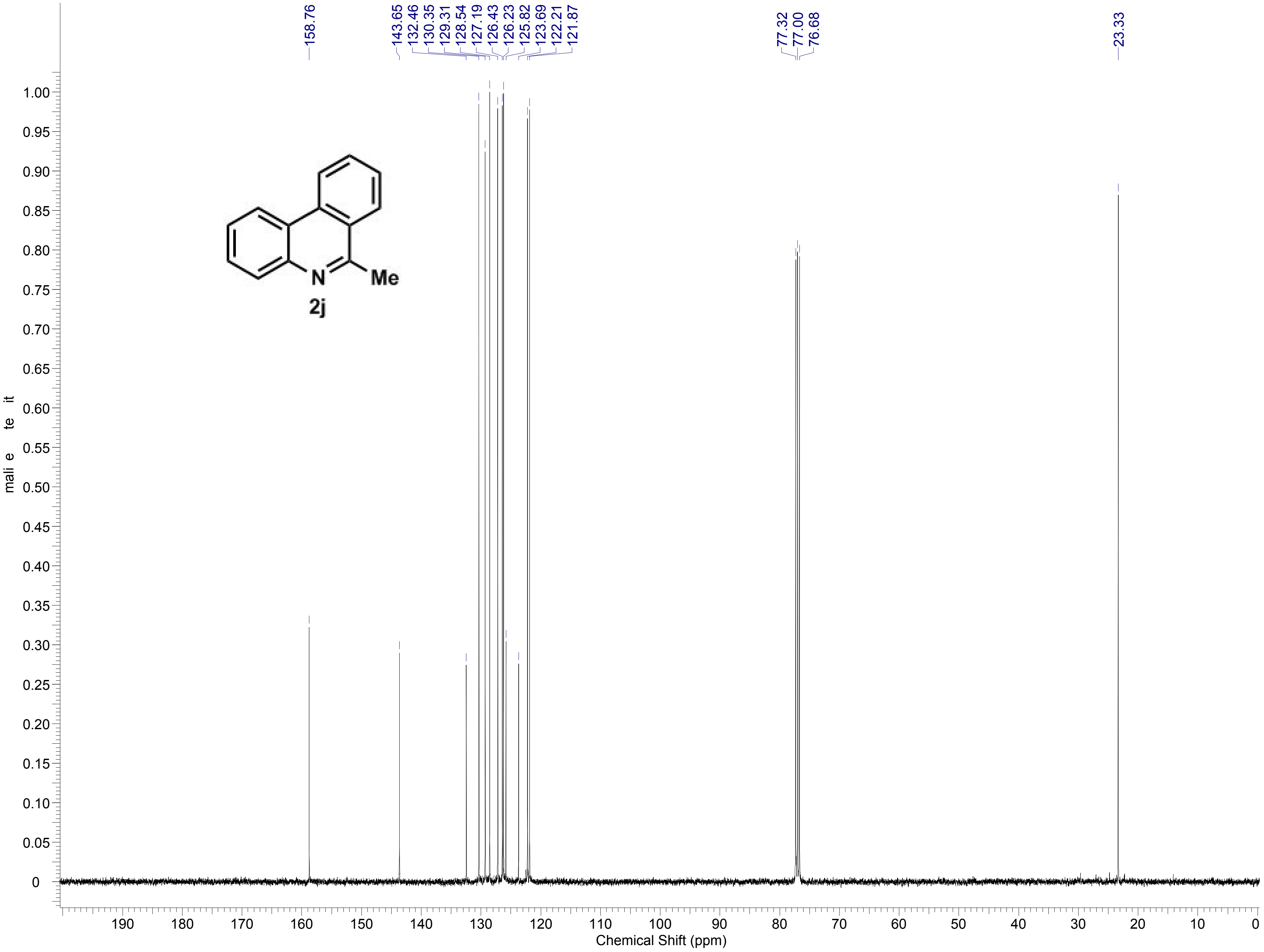
158.76
143.65
132.46
130.35
129.31
128.54
127.19
126.43
126.23
125.82
123.69
122.21
121.87
77.32
77.00
76.68
23.33
N
Me
2j
Chemical Shift (ppm)

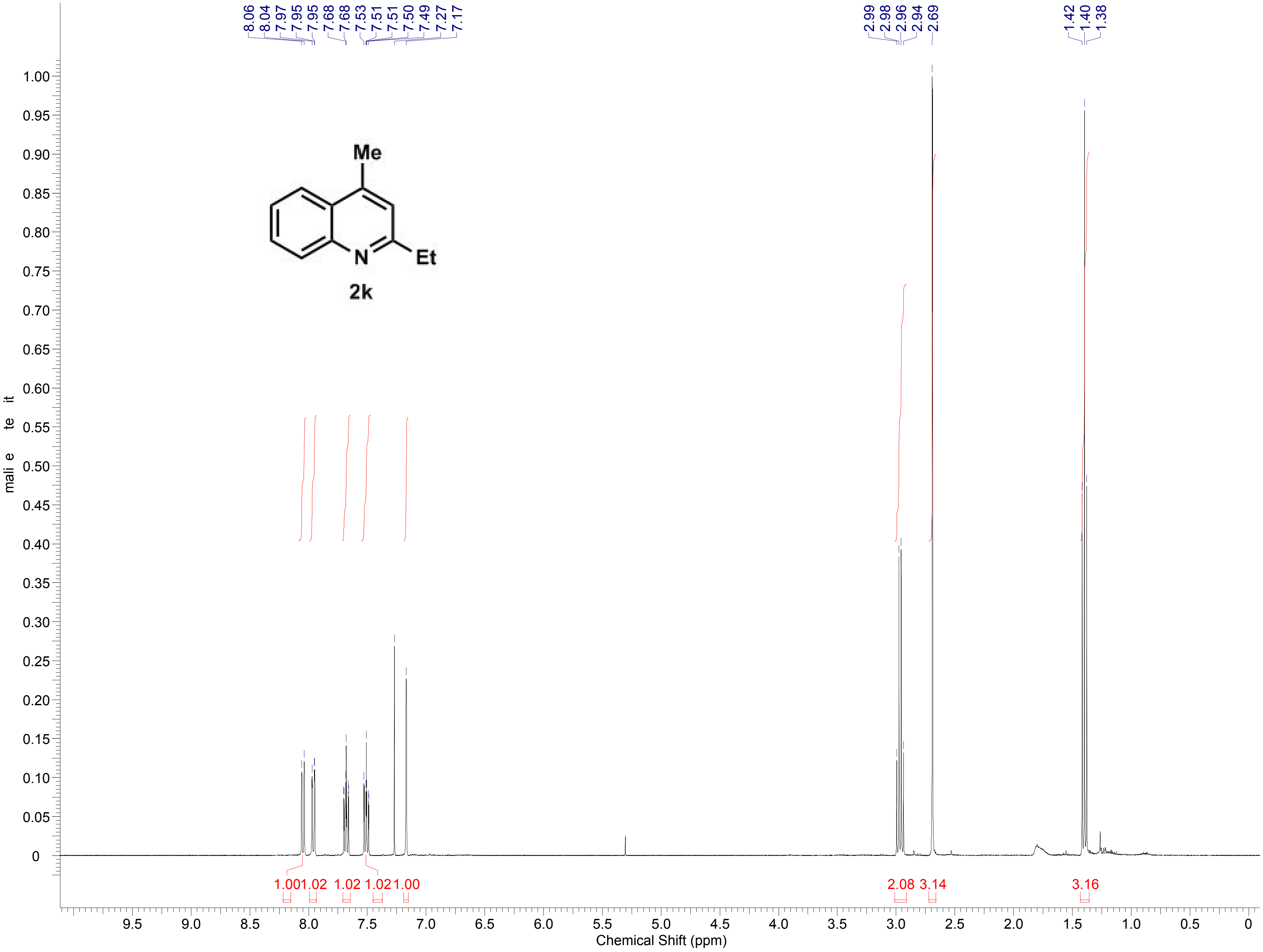

8.06 8.04 7.97 7.95 7.95 7.68 7.68 7.53 7.51 7.51 7.50 7.49 7.27 7.17
2.99 2.98 2.96 2.94 2.69
1.42 1.40 1.38
Me
N
Et
2k
1.00 1.02 1.02 1.02 1.00
2.08 3.14
3.16
1.00 0.95 0.90 0.85 0.80 0.75 0.70 0.65 0.60 0.55 0.50 0.45 0.40 0.35 0.30 0.25 0.20 0.15 0.10 0.05 0
9.5 9.0 8.5 8.0 7.5 7.0 6.5 6.0 5.5 5.0 4.5 4.0 3.5 3.0 2.5 2.0 1.5 1.0 0.5 0
Chemical Shift (ppm)

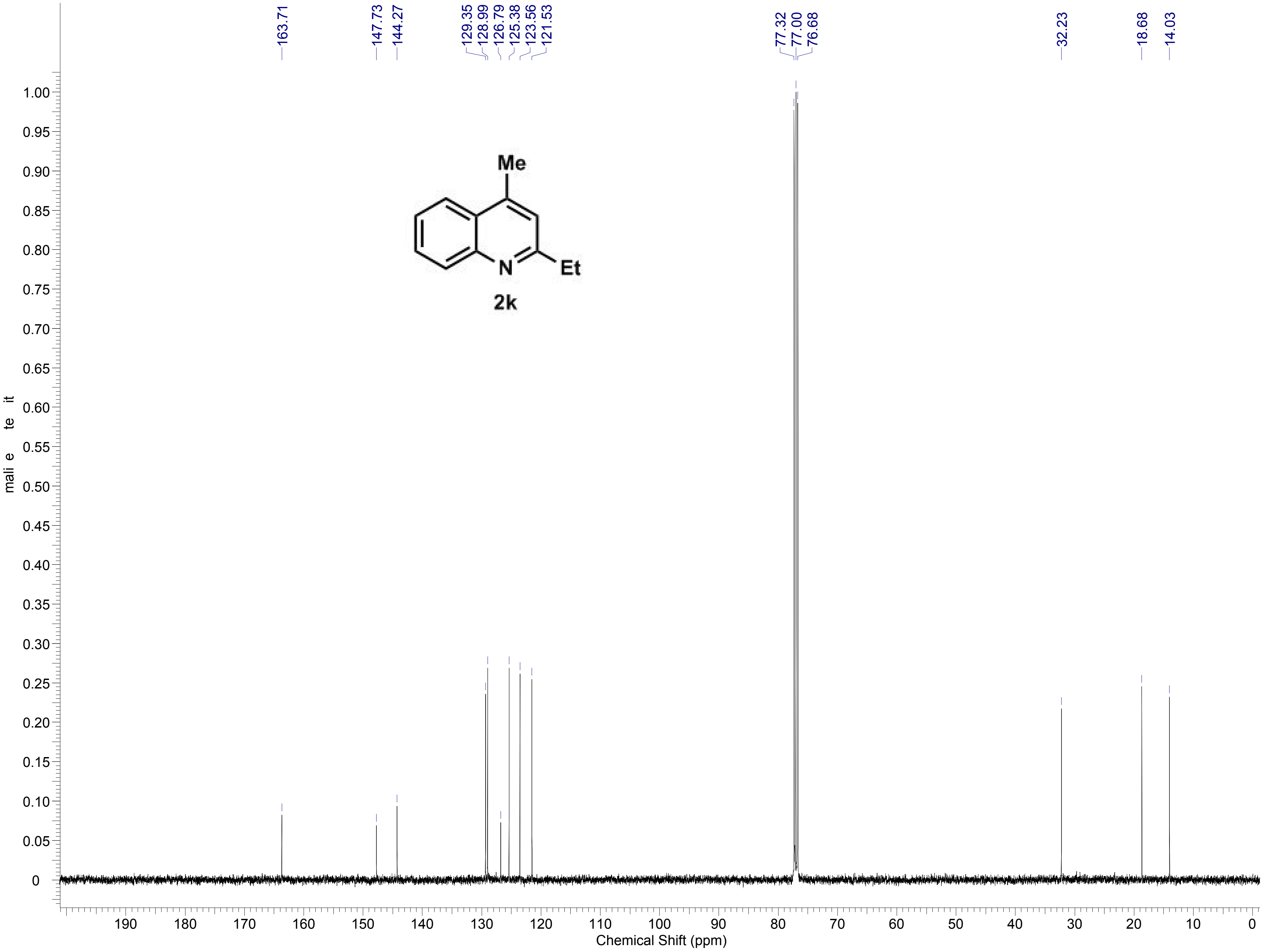
163.71
147.73
144.27
129.35
128.99
126.79
125.38
123.56
121.53
77.32
77.00
76.68
32.23
18.68
14.03
Me
N
Et
2k
Chemical Shift (ppm)

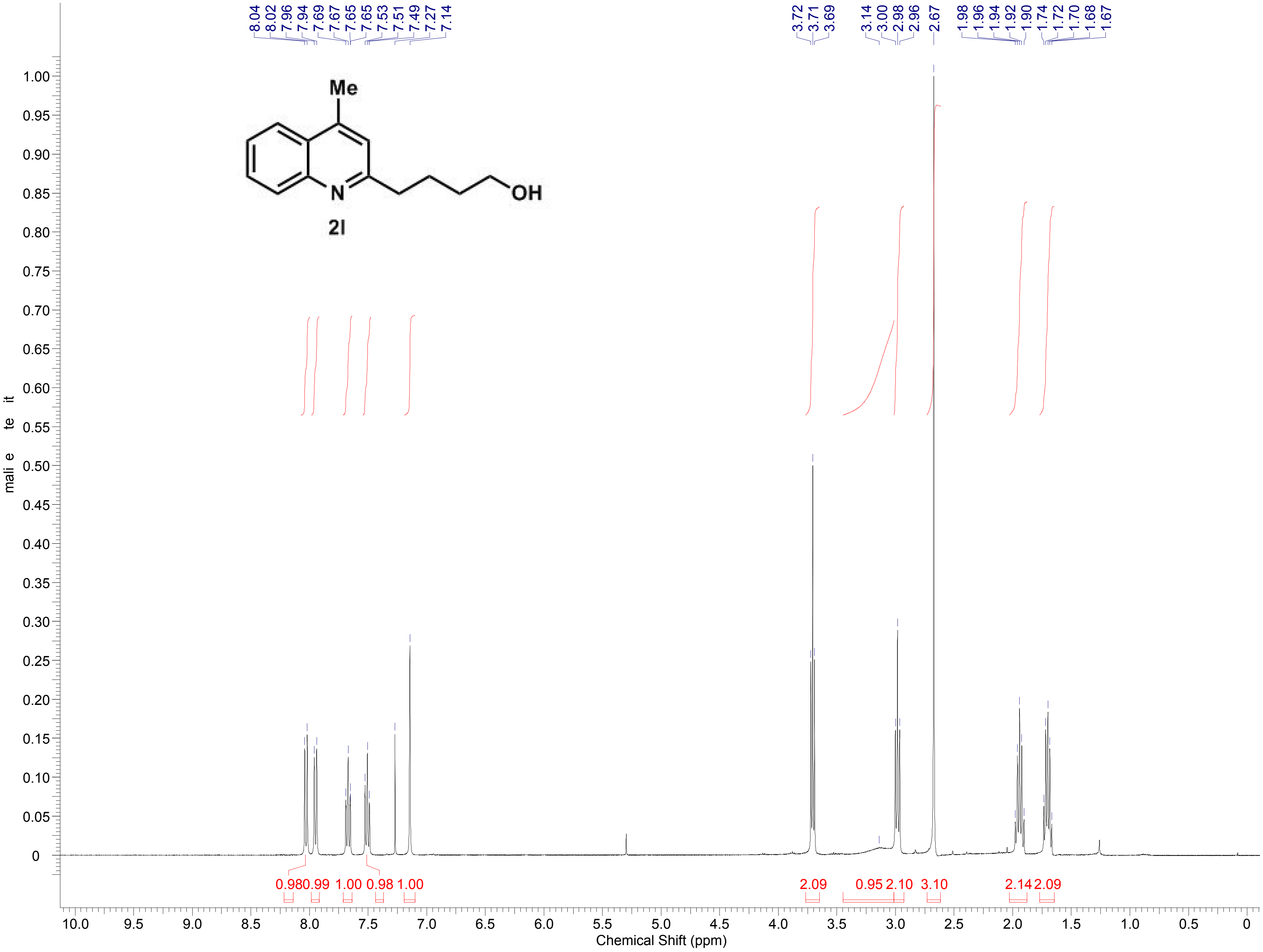

8.04
8.02
7.96
7.94
7.69
7.67
7.65
7.65
7.53
7.51
7.49
7.27
7.14
3.72
3.71
3.69
3.14
3.00
2.98
2.96
2.67
1.98
1.96
1.94
1.92
1.90
1.74
1.72
1.70
1.68
1.67
Me
N
OH
2l
0.98
0.99
1.00
0.98
1.00
2.09
0.95
2.10
3.10
2.14
2.09
Chemical Shift (ppm)

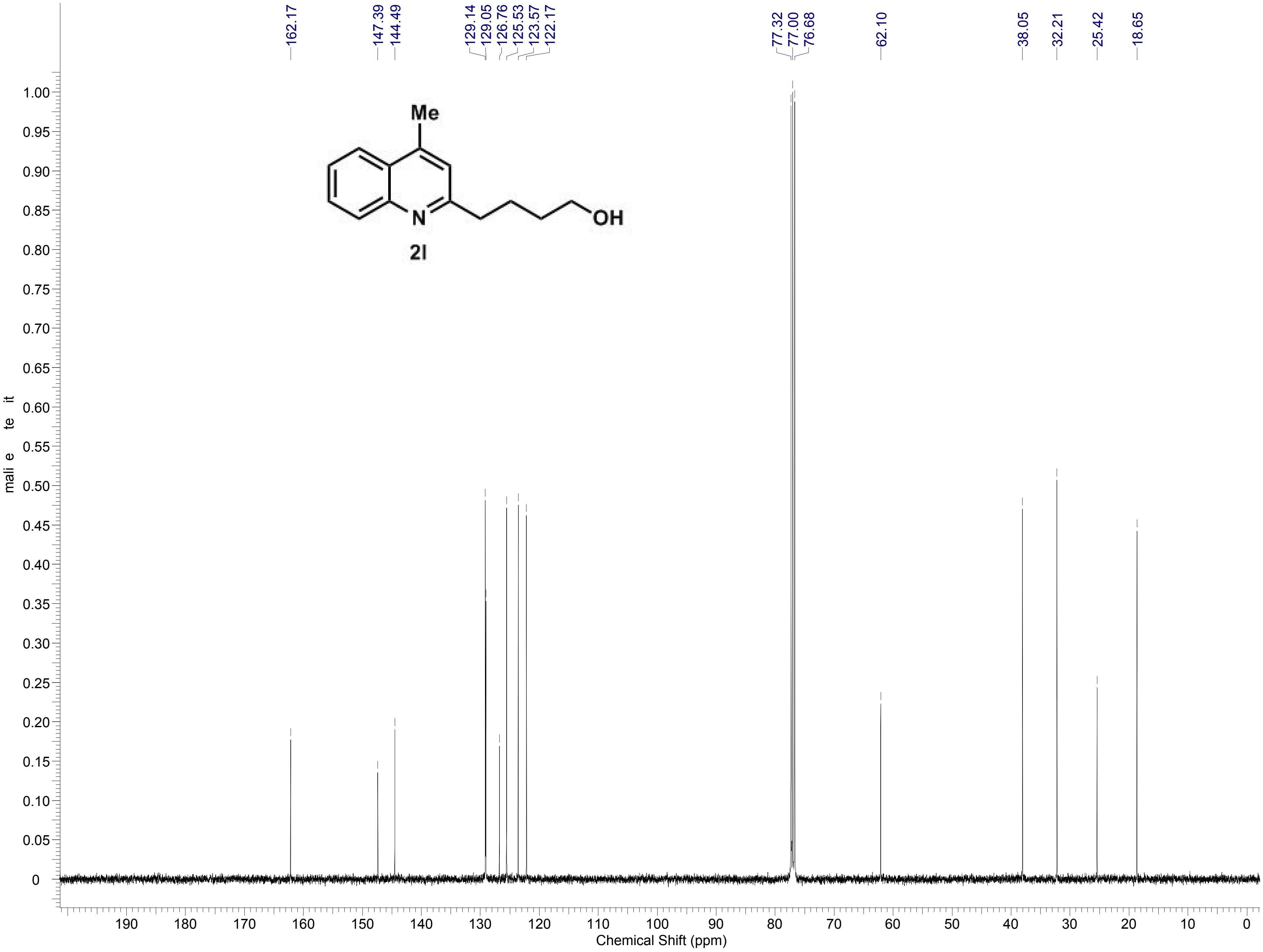
162.17
147.39
144.49
129.14
129.05
126.76
125.53
123.57
122.17
77.32
77.00
76.68
62.10
38.05
32.21
25.42
18.65
Me
N
OH
2l
Chemical Shift (ppm)

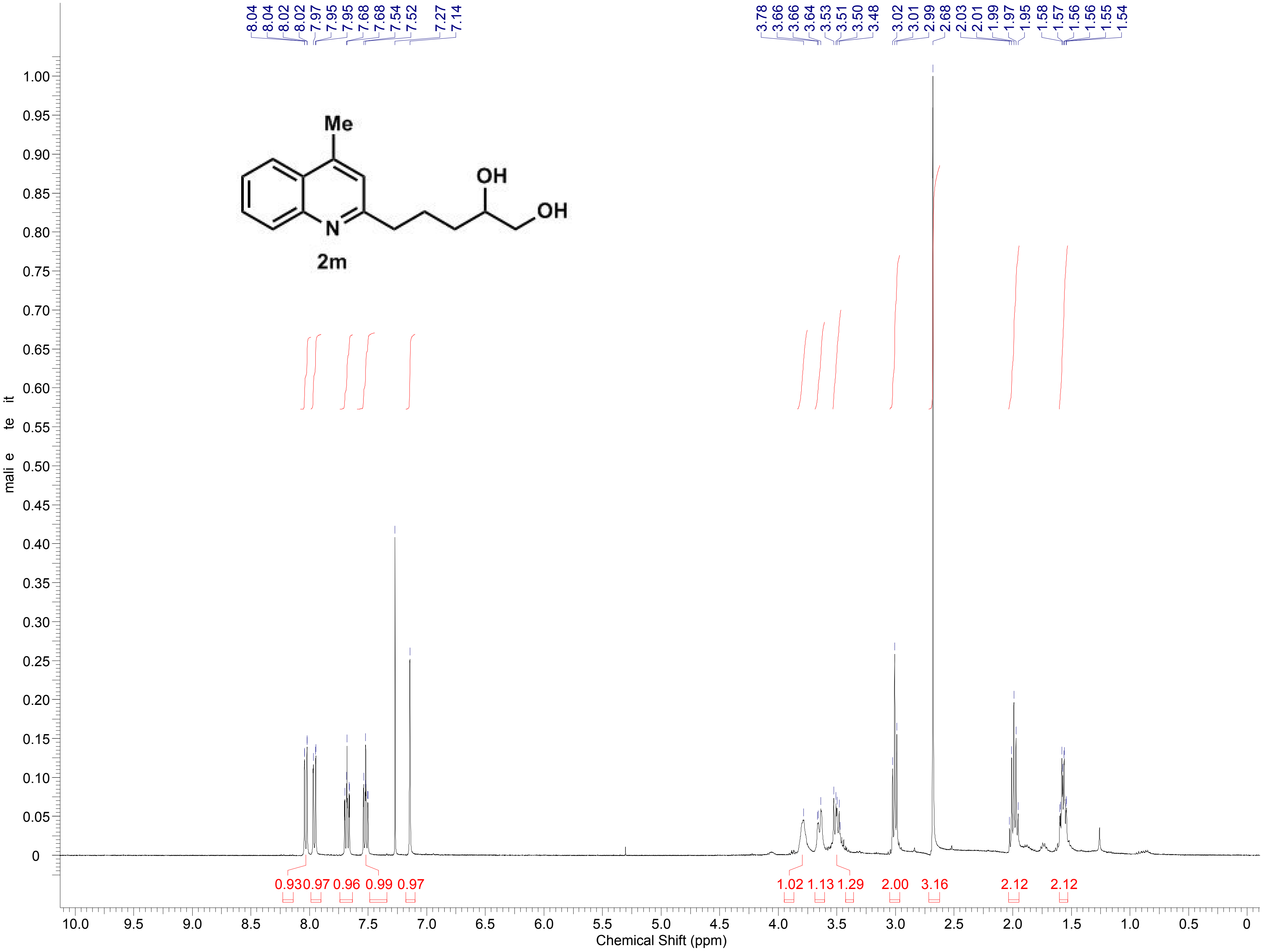
8.04 8.04 8.02 8.02 7.97 7.95 7.95 7.68 7.68 7.54 7.52 7.27 7.14
3.78 3.66 3.66 3.64 3.53 3.51 3.50 3.48 3.02 3.01 2.99 2.68 2.03 2.01 1.99 1.97 1.95 1.58 1.57 1.56 1.56 1.55 1.54
Me
OH
OH
N
2m
0.93 0.97 0.96 0.99 0.97
1.02 1.13 1.29 2.00 3.16 2.12 2.12
Chemical Shift (ppm)

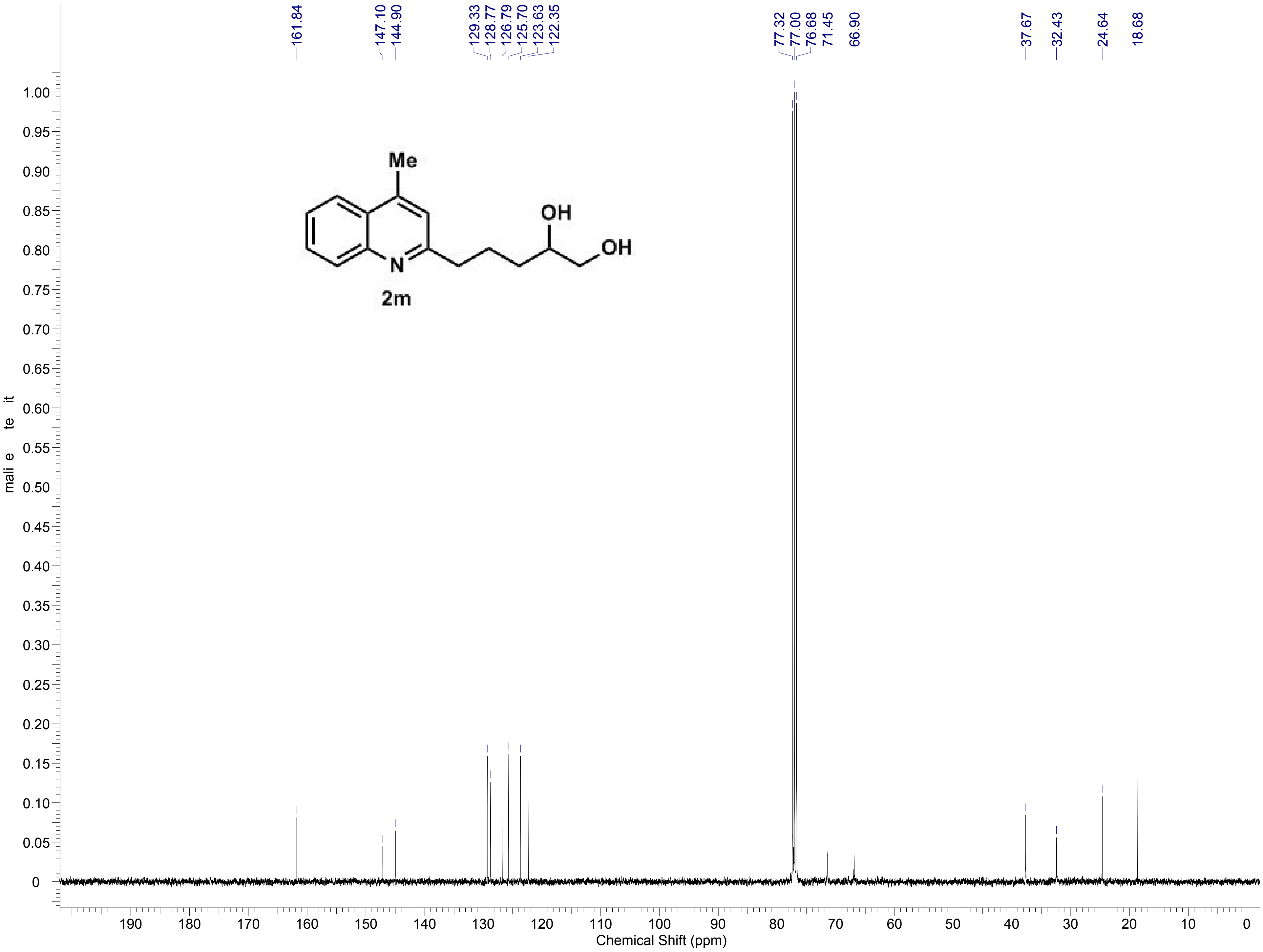
161.84
147.10
144.90
129.33
128.77
126.79
125.70
123.63
122.35
77.32
77.00
76.68
71.45
66.90
37.67
32.43
24.64
18.68
Me
OH
OH
N
2m
Chemical Shift (ppm)

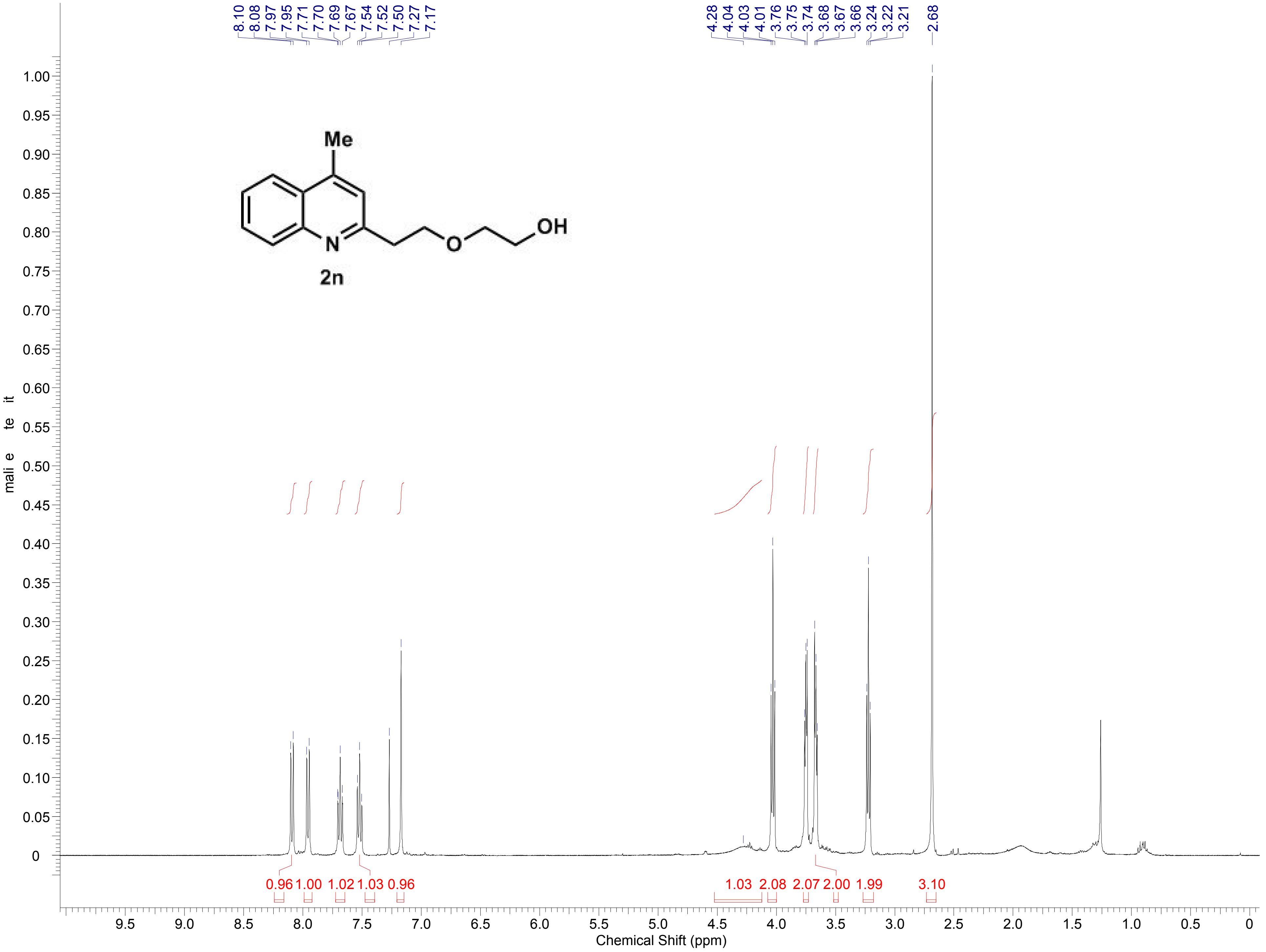

8.10
8.08
7.97
7.95
7.71
7.70
7.69
7.67
7.54
7.52
7.50
7.27
7.17
4.28
4.04
4.03
4.01
3.76
3.75
3.74
3.68
3.67
3.66
3.24
3.22
3.21
2.68
Me
N
O
OH
2n
0.96
1.00
1.02
1.03
0.96
1.03
2.08
2.07
2.00
1.99
3.10
Chemical Shift (ppm)

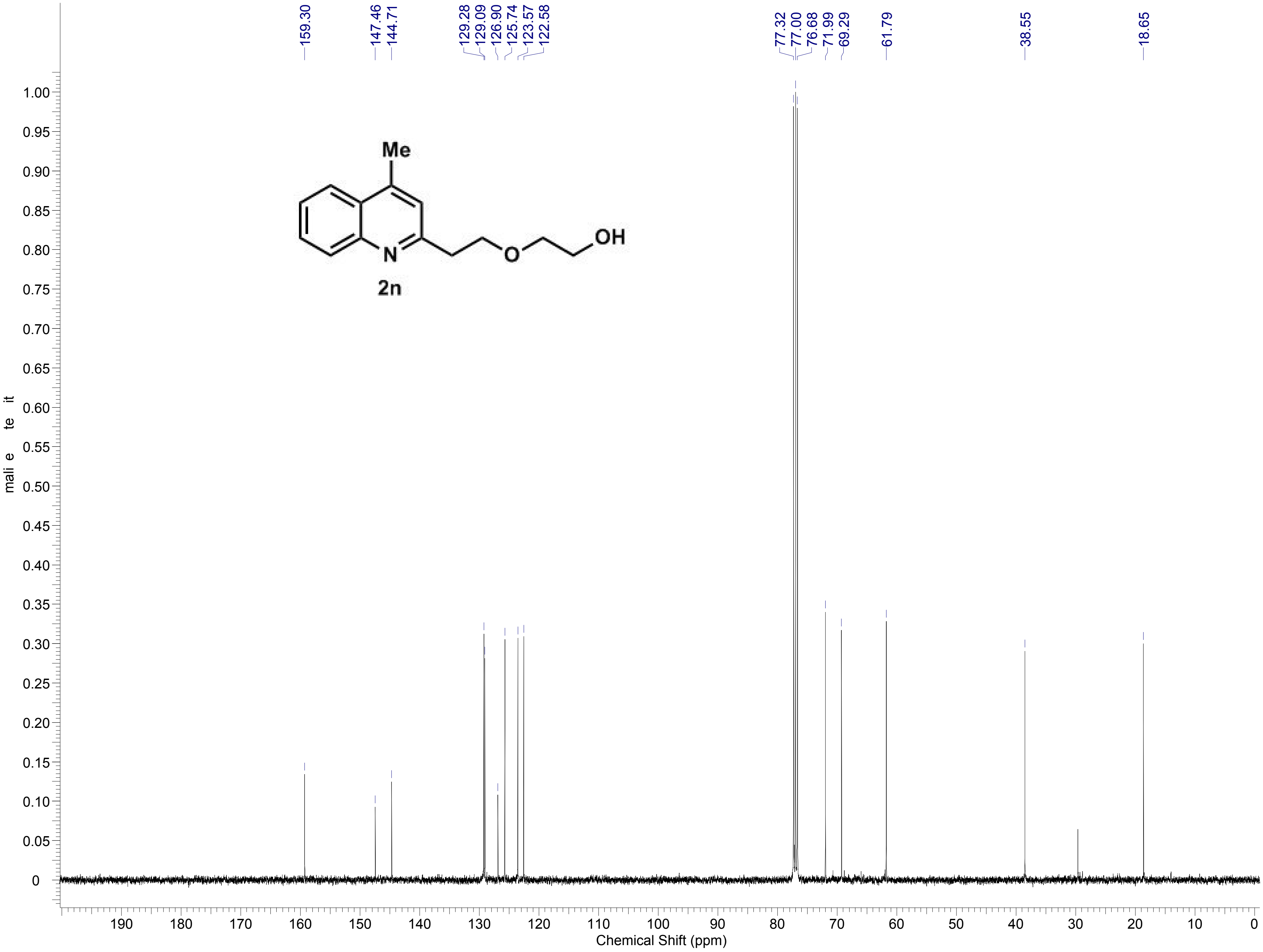

159.30
147.46
144.71
129.28
129.09
126.90
125.74
123.57
122.58
77.32
77.00
76.68
71.99
69.29
61.79
38.55
18.65
Me
N
O
OH
2n
Chemical Shift (ppm)

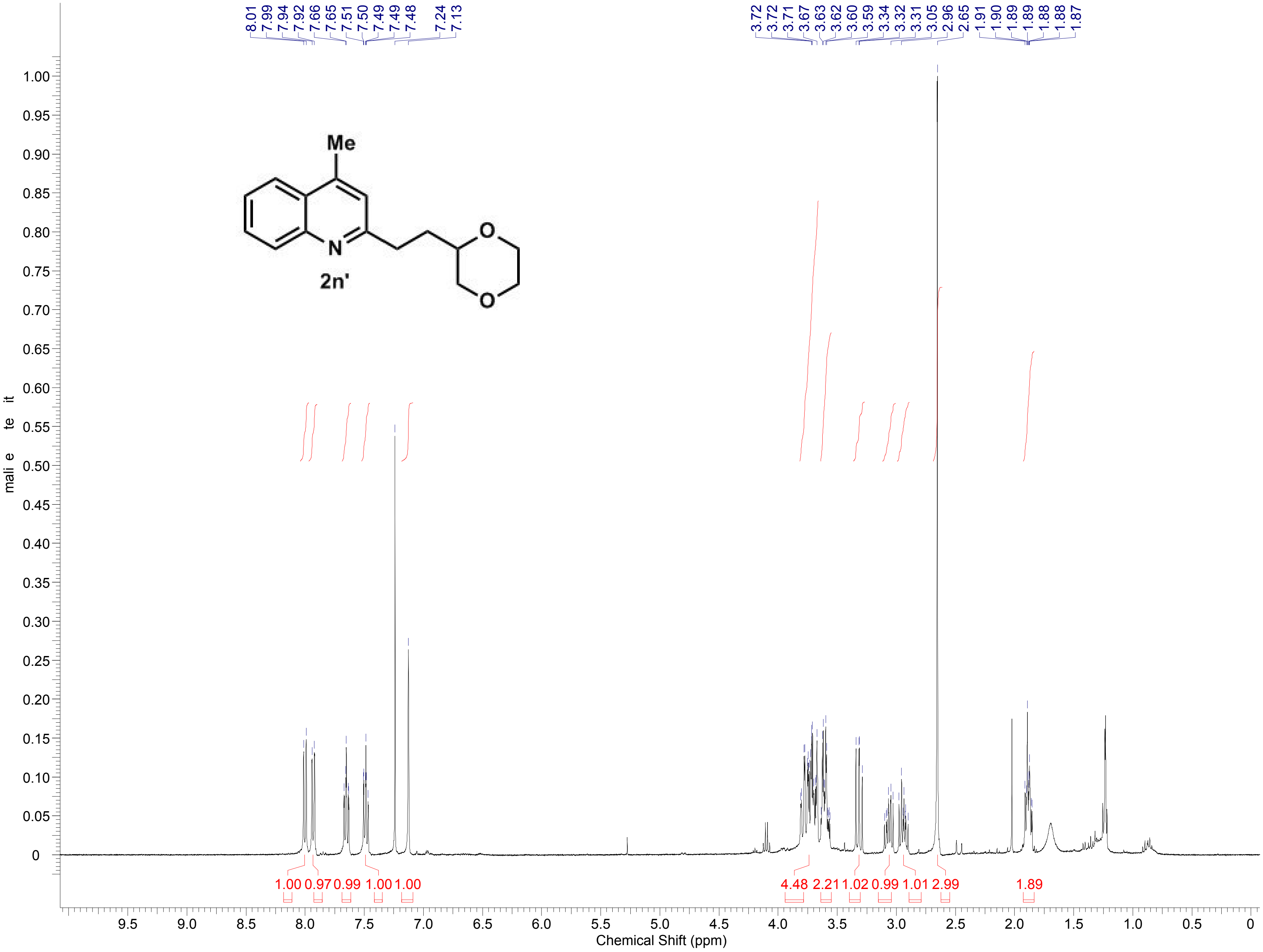
8.01 7.99 7.94 7.92 7.66 7.65 7.51 7.50 7.49 7.49 7.48 7.24 7.13
3.72 3.72 3.71 3.67 3.63 3.62 3.60 3.59 3.34 3.32 3.31 3.05 2.96 2.65 1.91 1.90 1.89 1.89 1.88 1.88 1.87
Me
N
O
O
2n'
1.00 0.97 0.99 1.00 1.00
4.48 2.21 1.02 0.99 1.01 2.99
1.89
Chemical Shift (ppm)

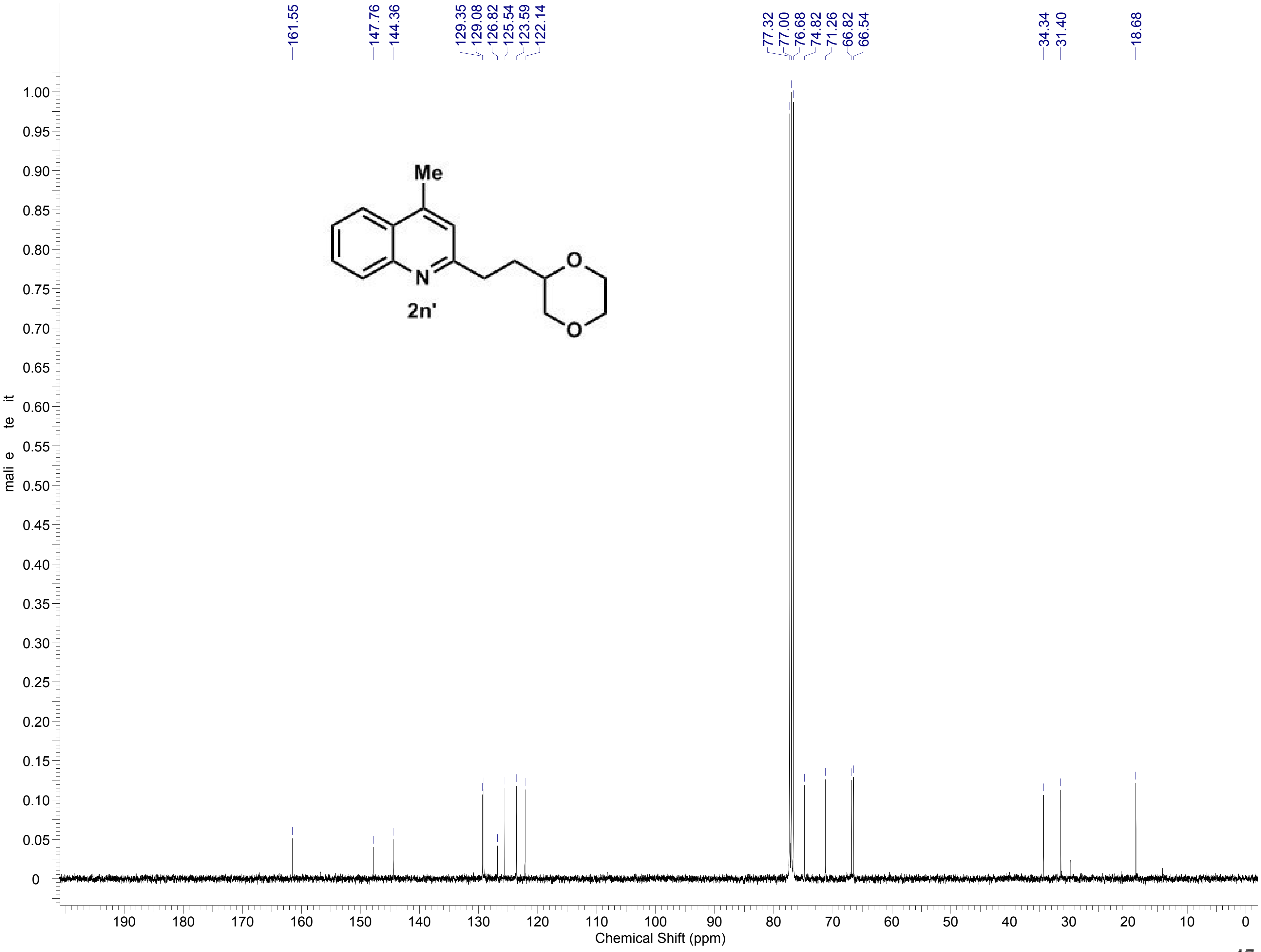
161.55
147.76
144.36
129.35
129.08
126.82
125.54
123.59
122.14
77.32
77.00
76.68
74.82
71.26
66.82
66.54
34.34
31.40
18.68
Me
N
O
O
2n'
Chemical Shift (ppm)

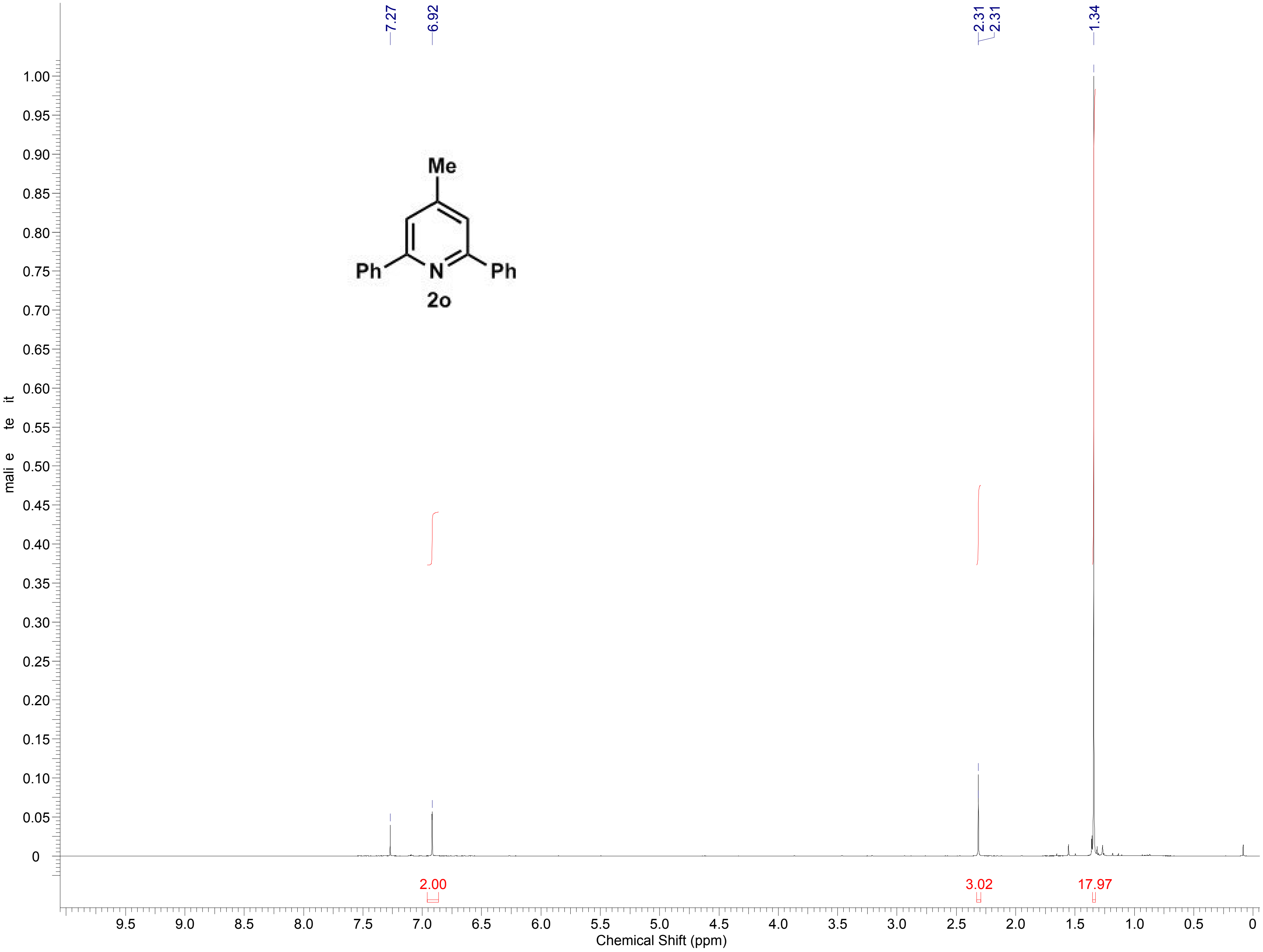
7.27
6.92
2.31
2.31
1.34
Me
Ph
N
Ph
2o
mali e te it
2.00
3.02
17.97
Chemical Shift (ppm)

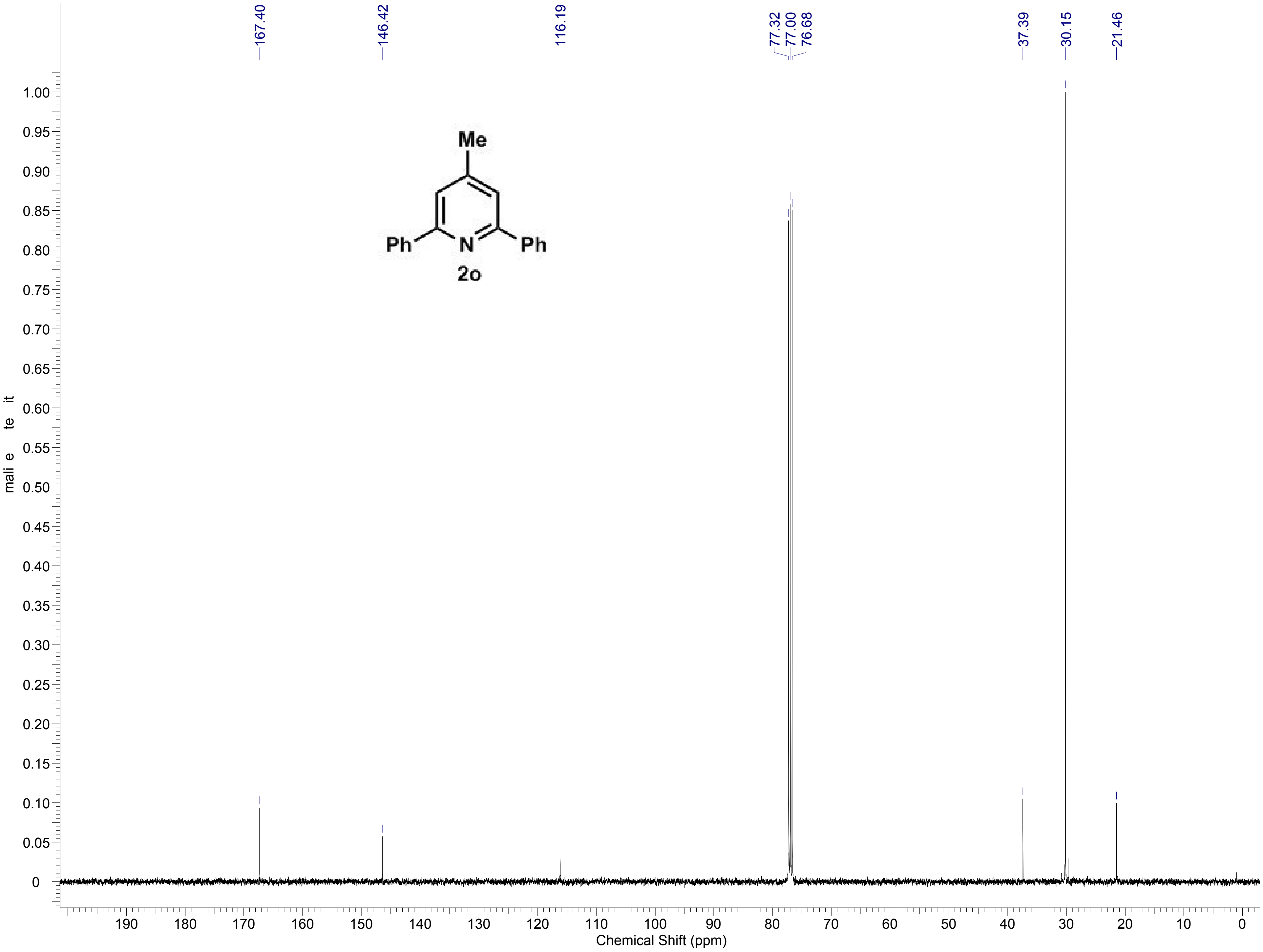
167.40
146.42
116.19
77.32
77.00
76.68
37.39
30.15
21.46
Me
Ph
N
Ph
2o
Chemical Shift (ppm)

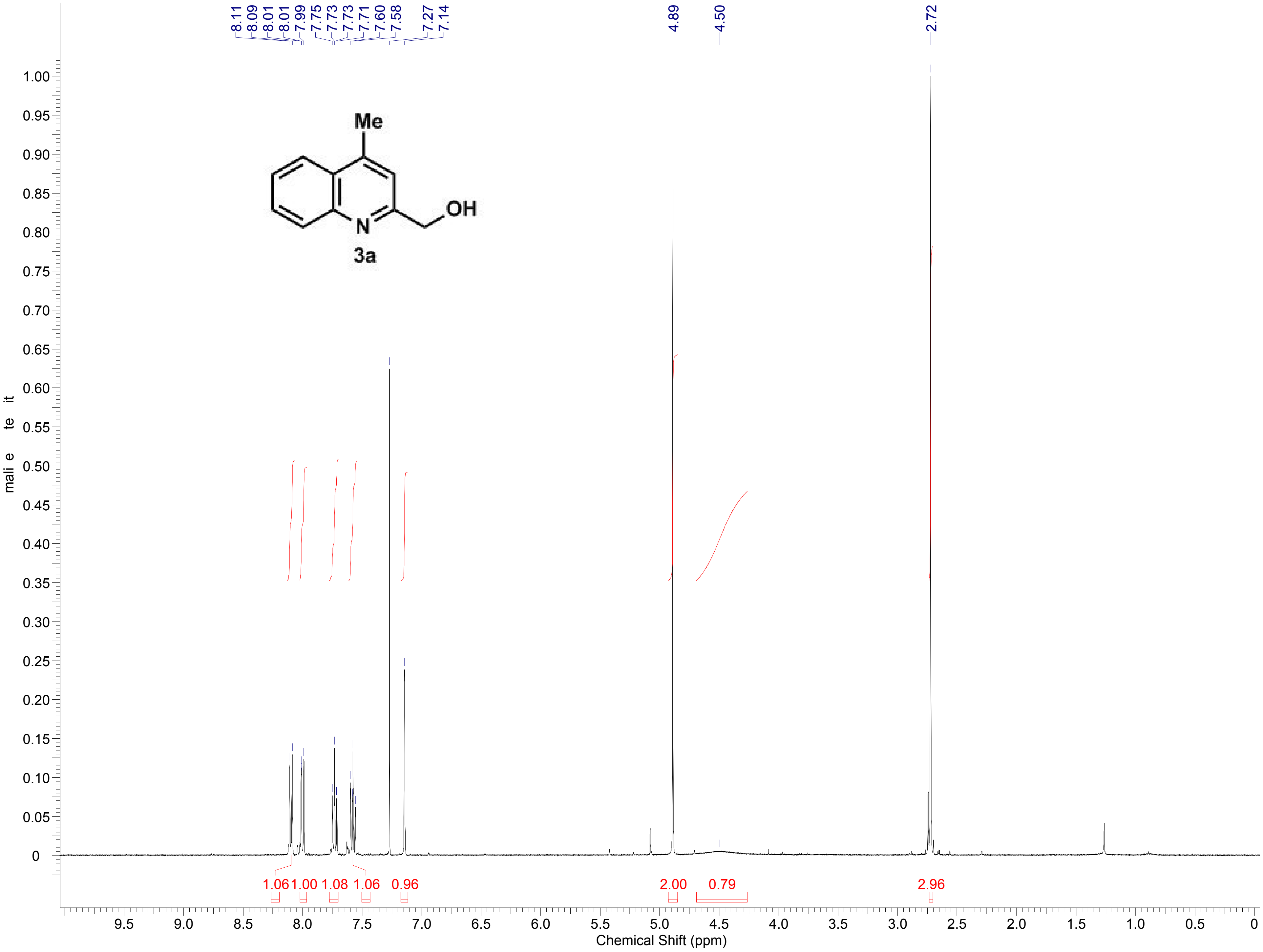
8.11
8.09
8.01
8.01
7.99
7.75
7.73
7.73
7.71
7.60
7.58
7.27
7.14
4.89
4.50
2.72
Me
OH
N
3a
1.06
1.00
1.08
1.06
0.96
2.00
0.79
2.96
Chemical Shift (ppm)

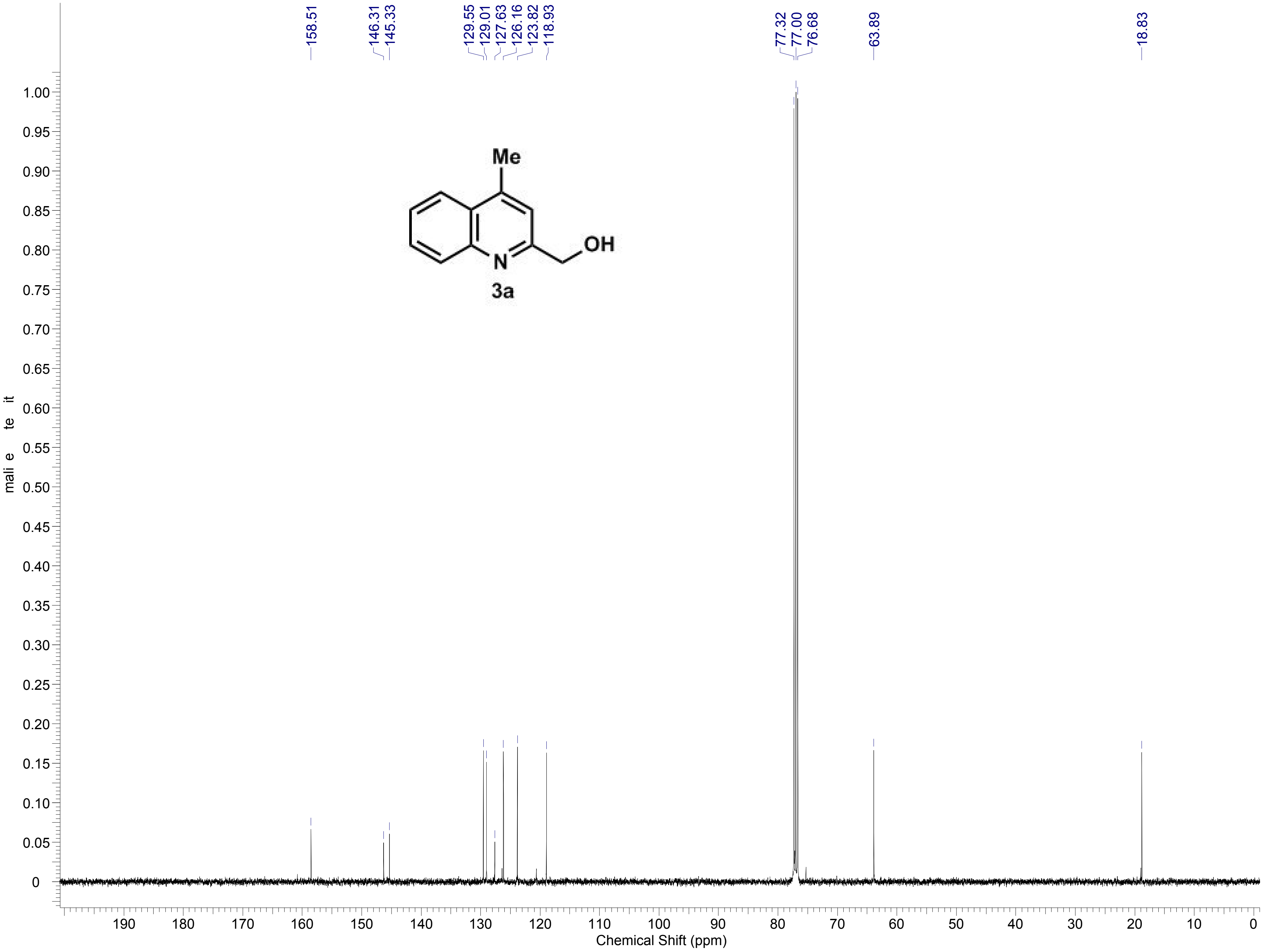
158.51
146.31
145.33
129.55
129.01
127.63
126.16
123.82
118.93
77.32
77.00
76.68
63.89
18.83
Me
OH
N
3a
Chemical Shift (ppm)

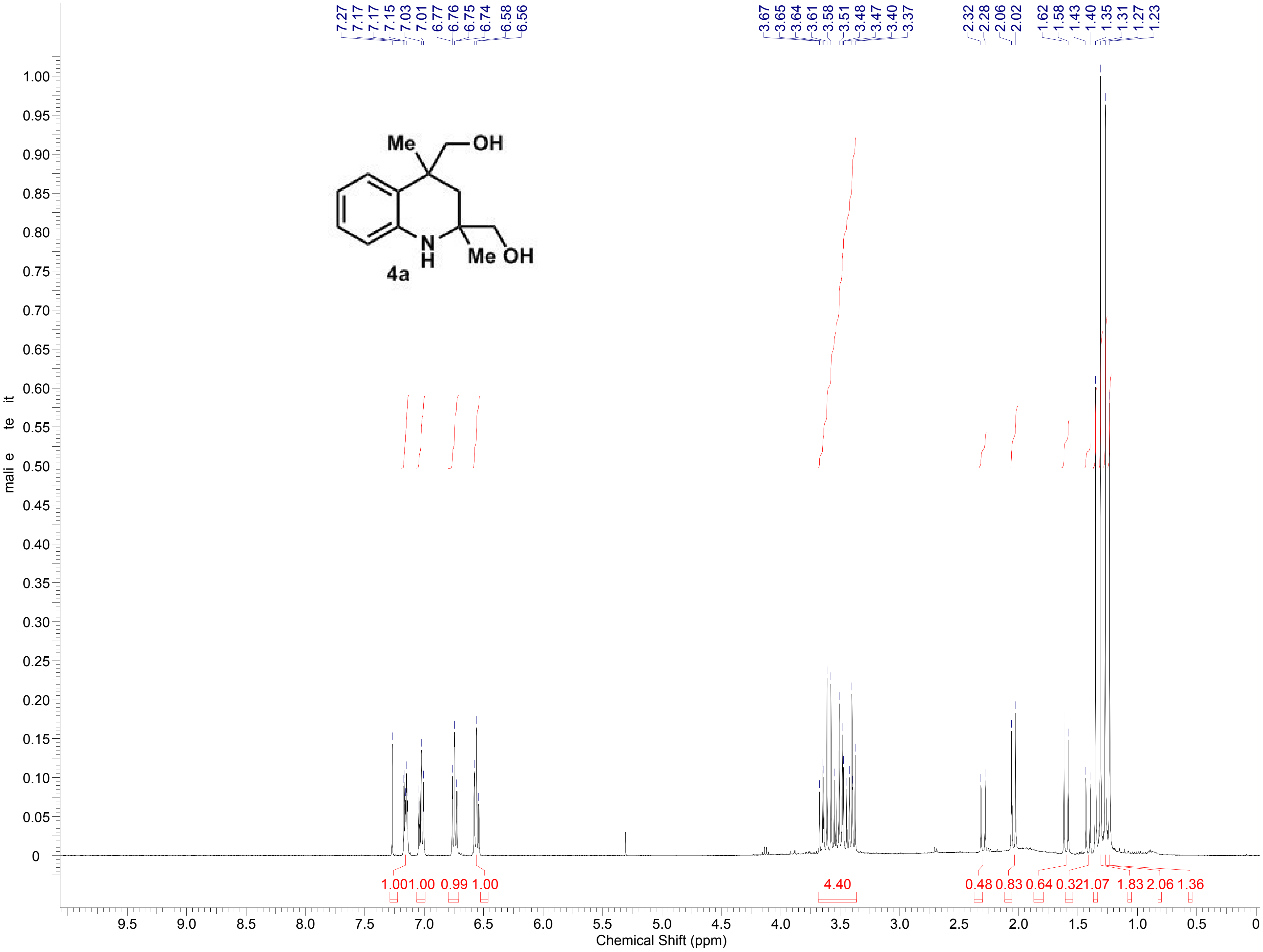
7.27
7.17
7.17
7.15
7.03
7.01
6.77
6.76
6.75
6.74
6.58
6.56
3.67
3.65
3.64
3.61
3.58
3.51
3.48
3.47
3.40
3.37
2.32
2.28
2.06
2.02
1.62
1.58
1.43
1.40
1.35
1.31
1.27
1.23
Me
OH
N
H
Me
OH
4a
1.00
1.00
0.99
1.00
4.40
0.48
0.83
0.64
0.32
1.07
1.83
2.06
1.36
Chemical Shift (ppm)

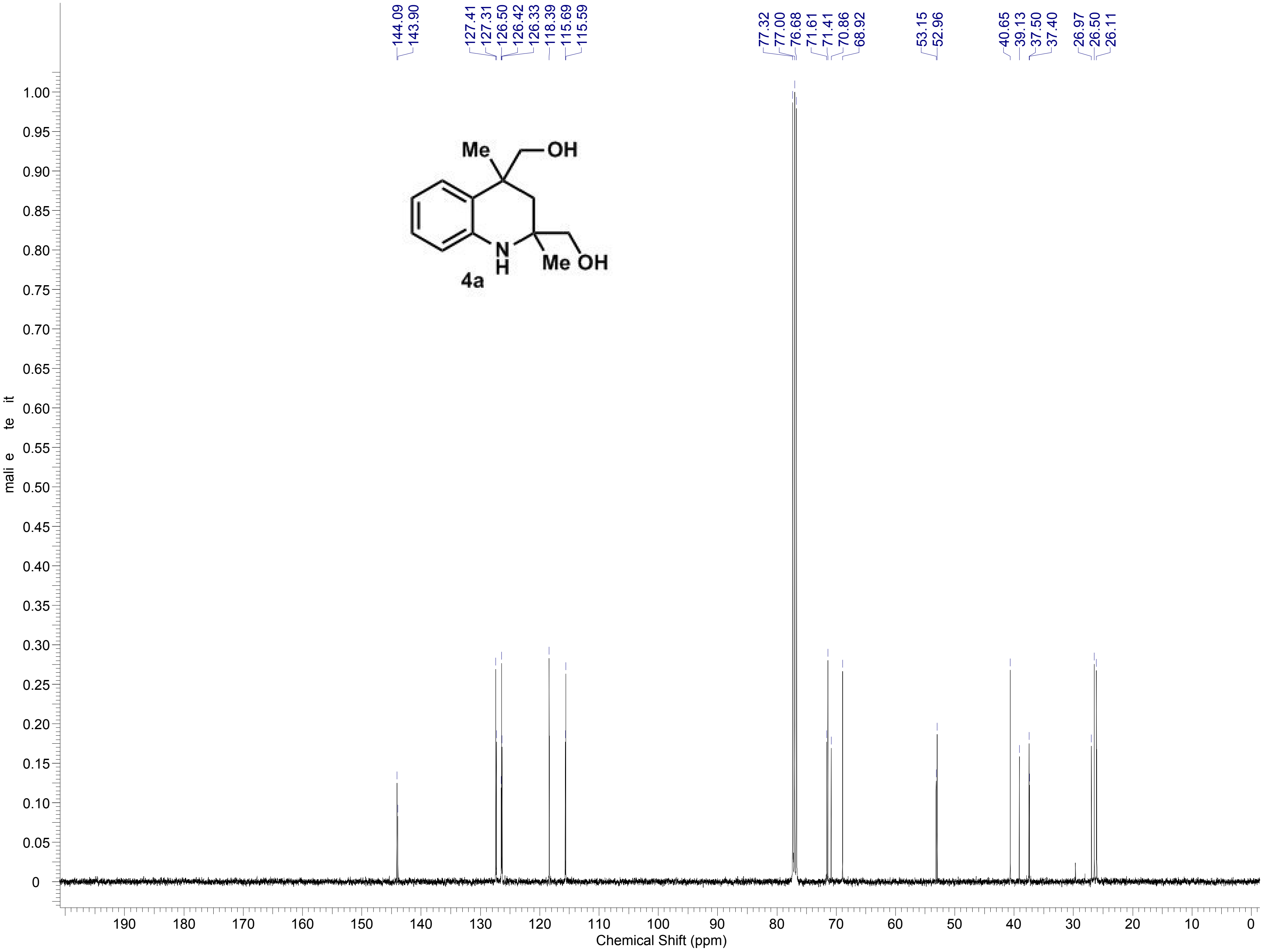
144.09
143.90
127.41
127.31
126.50
126.42
126.33
118.39
115.69
115.59
77.32
77.00
76.68
71.61
71.41
70.86
68.92
53.15
52.96
40.65
39.13
37.50
37.40
26.97
26.50
26.11
Me
OH
N
H
Me OH
4a
mali e te it
Chemical Shift (ppm)

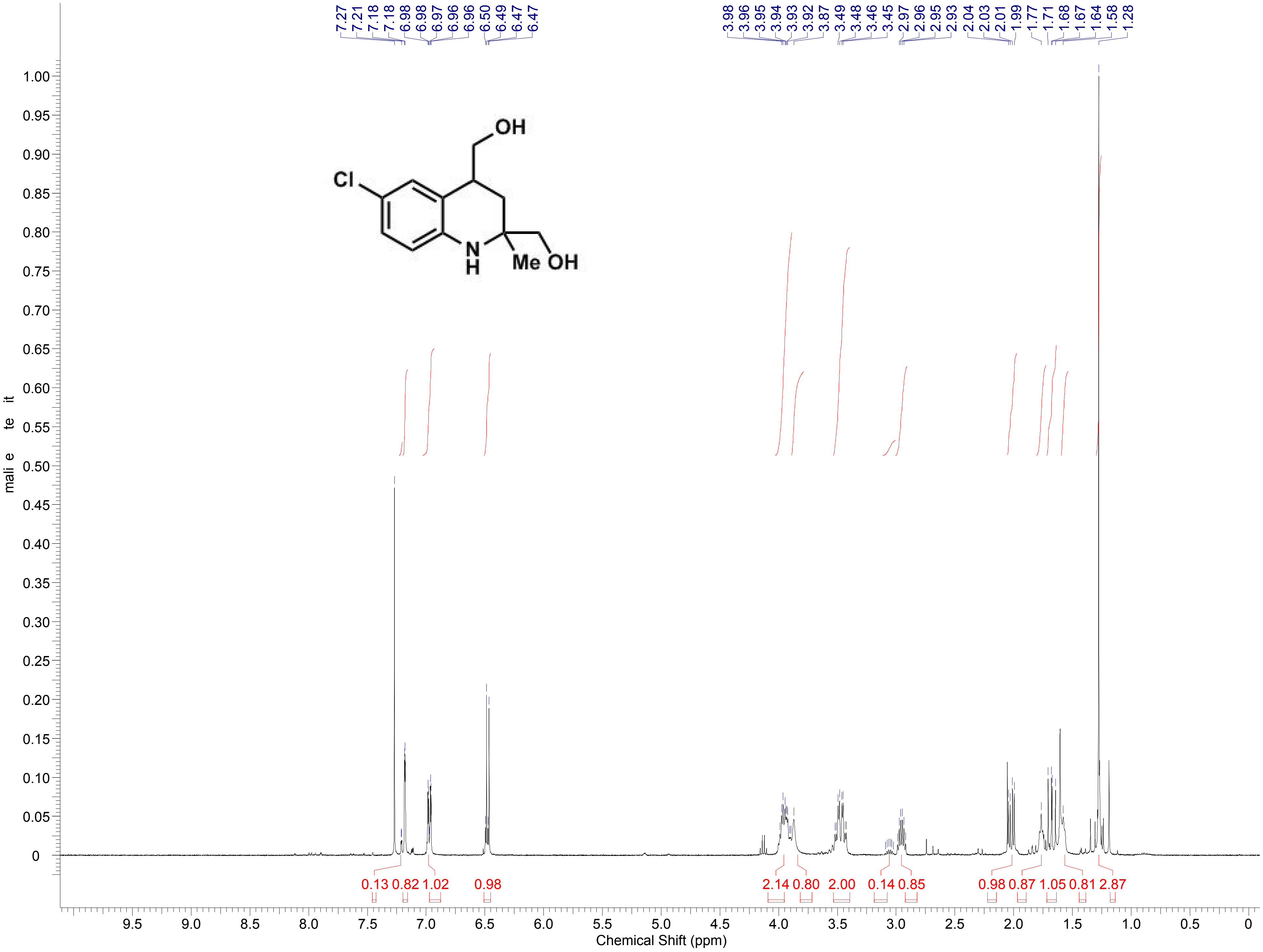
7.27 7.21 7.18 7.18 6.98 6.98 6.97 6.96 6.96 6.50 6.49 6.47 6.47
3.98 3.96 3.95 3.94 3.93 3.92 3.87 3.49 3.48 3.46 3.45 2.97 2.96 2.95 2.93 2.04 2.03 2.01 1.99 1.77 1.71 1.68 1.67 1.64 1.58 1.28
Cl
OH
N
H
Me
OH
0.13 0.82 1.02 0.98 2.14 0.80 2.00 0.14 0.85 0.98 0.87 1.05 0.81 2.87
Chemical Shift (ppm)

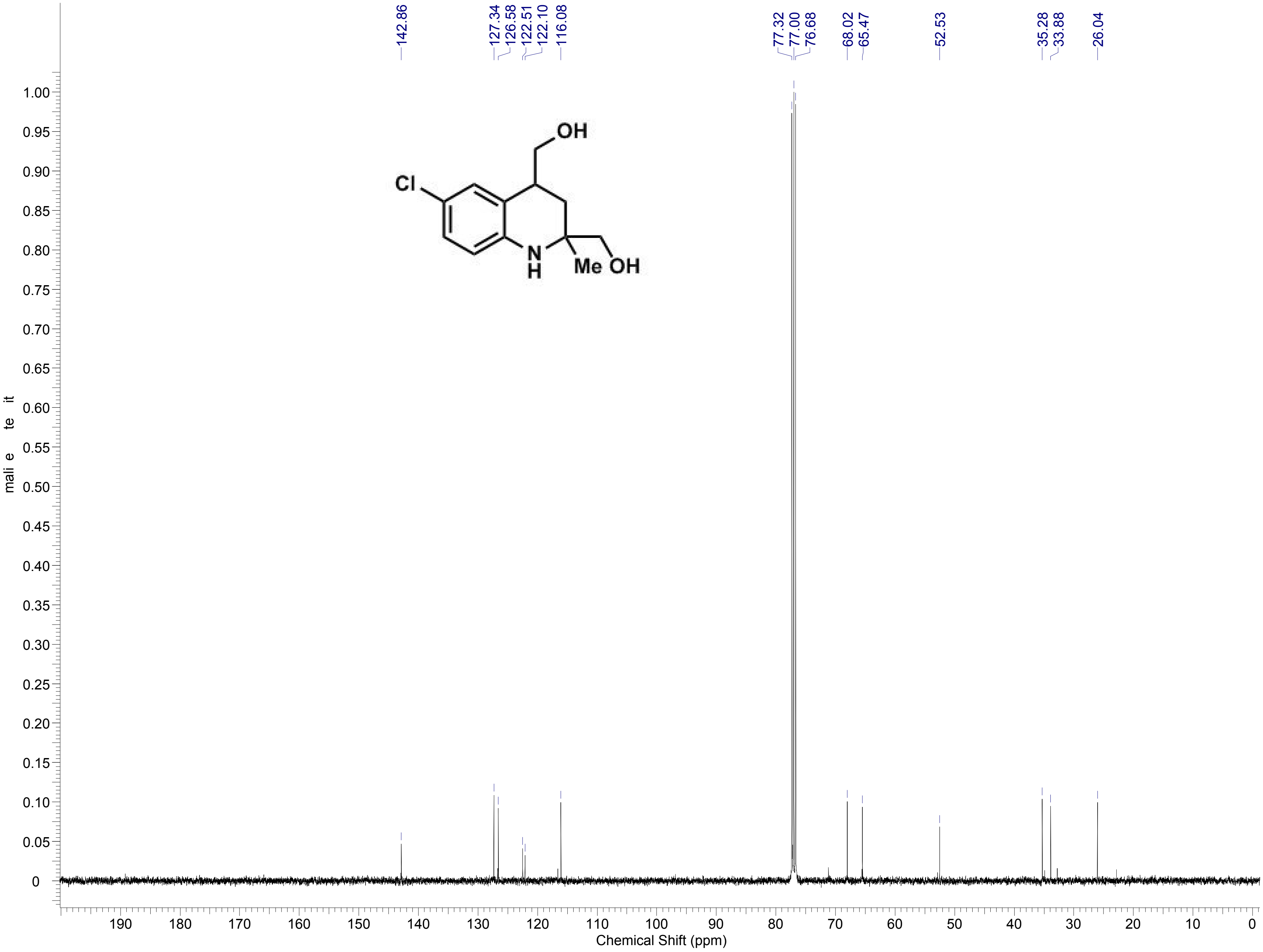
142.86
127.34
126.58
122.51
122.10
116.08
77.32
77.00
76.68
68.02
65.47
52.53
35.28
33.88
26.04
OH
Cl
N
H
Me
OH
Chemical Shift (ppm)

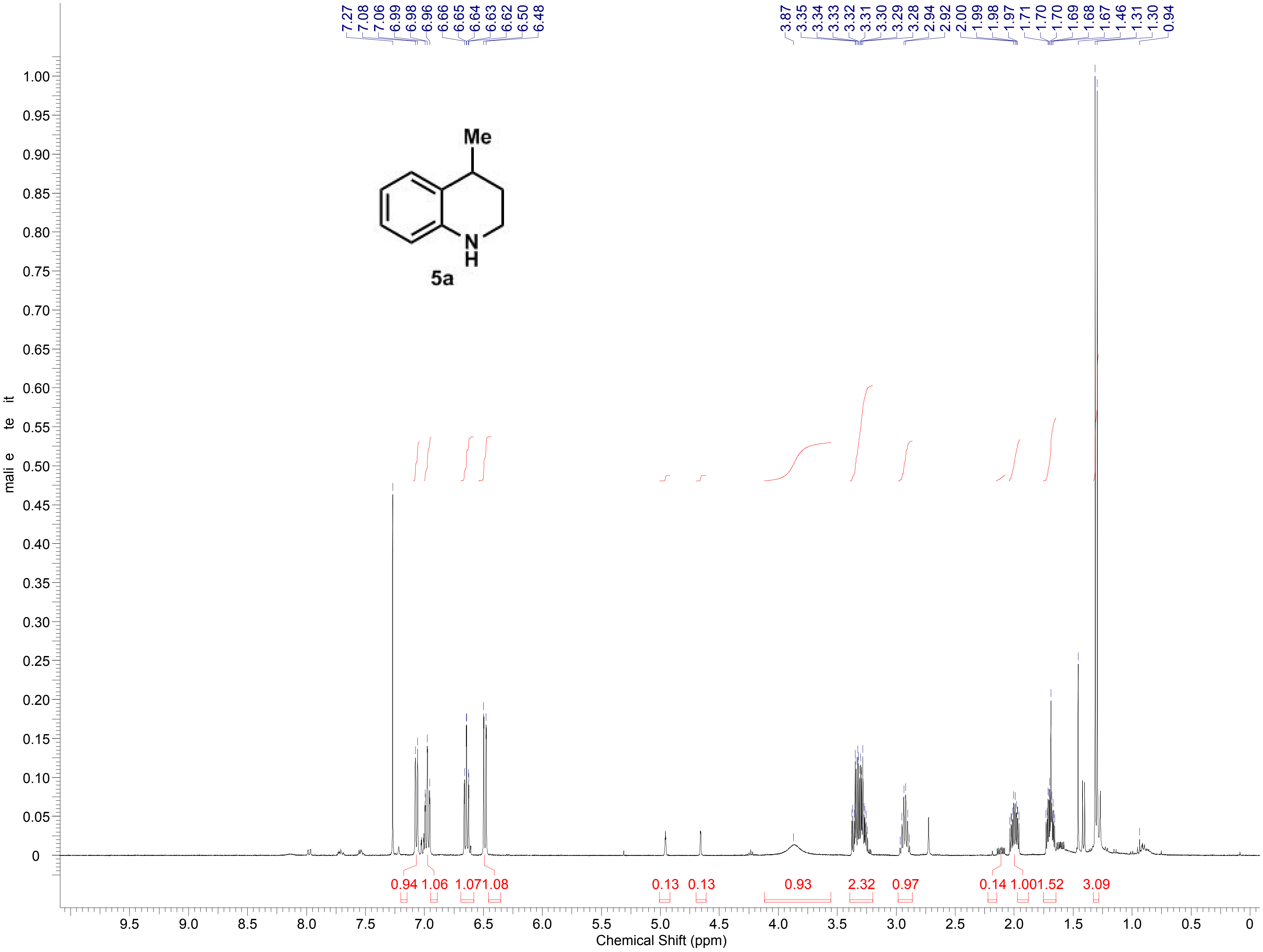
Me
N
H
5a
7.27
7.08
7.06
6.99
6.98
6.96
6.66
6.65
6.64
6.63
6.62
6.50
6.48
3.87
3.35
3.34
3.33
3.32
3.31
3.30
3.29
3.28
2.94
2.92
2.00
1.99
1.98
1.97
1.71
1.70
1.70
1.69
1.68
1.67
1.46
1.31
1.30
0.94
0.94
1.06
1.07
1.08
0.13
0.13
0.93
2.32
0.97
0.14
1.00
1.52
3.09
Chemical Shift (ppm)

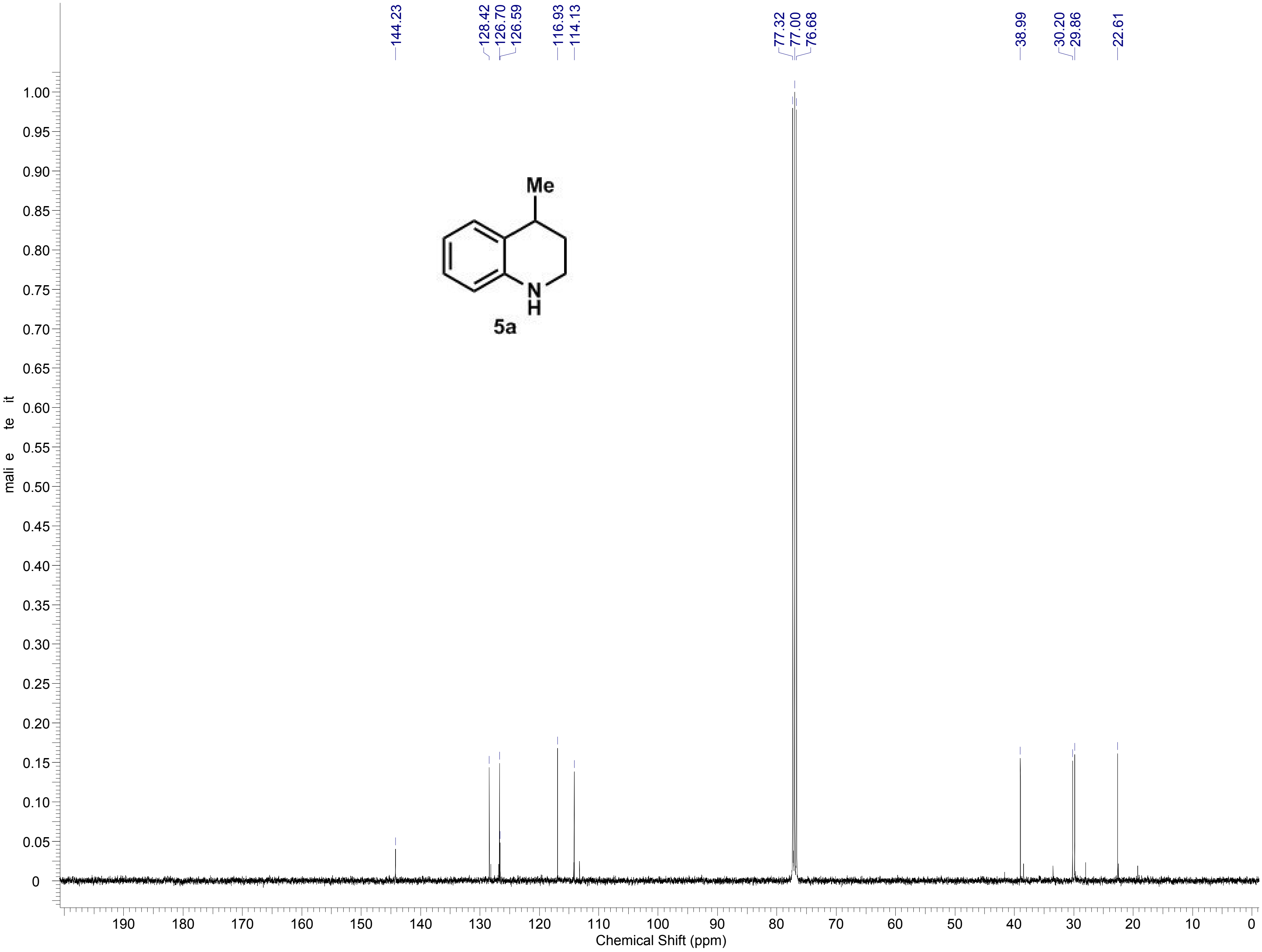
144.23
128.42
126.70
126.59
116.93
114.13
77.32
77.00
76.68
38.99
30.20
29.86
22.61
Me
N
H
5a
Chemical Shift (ppm)

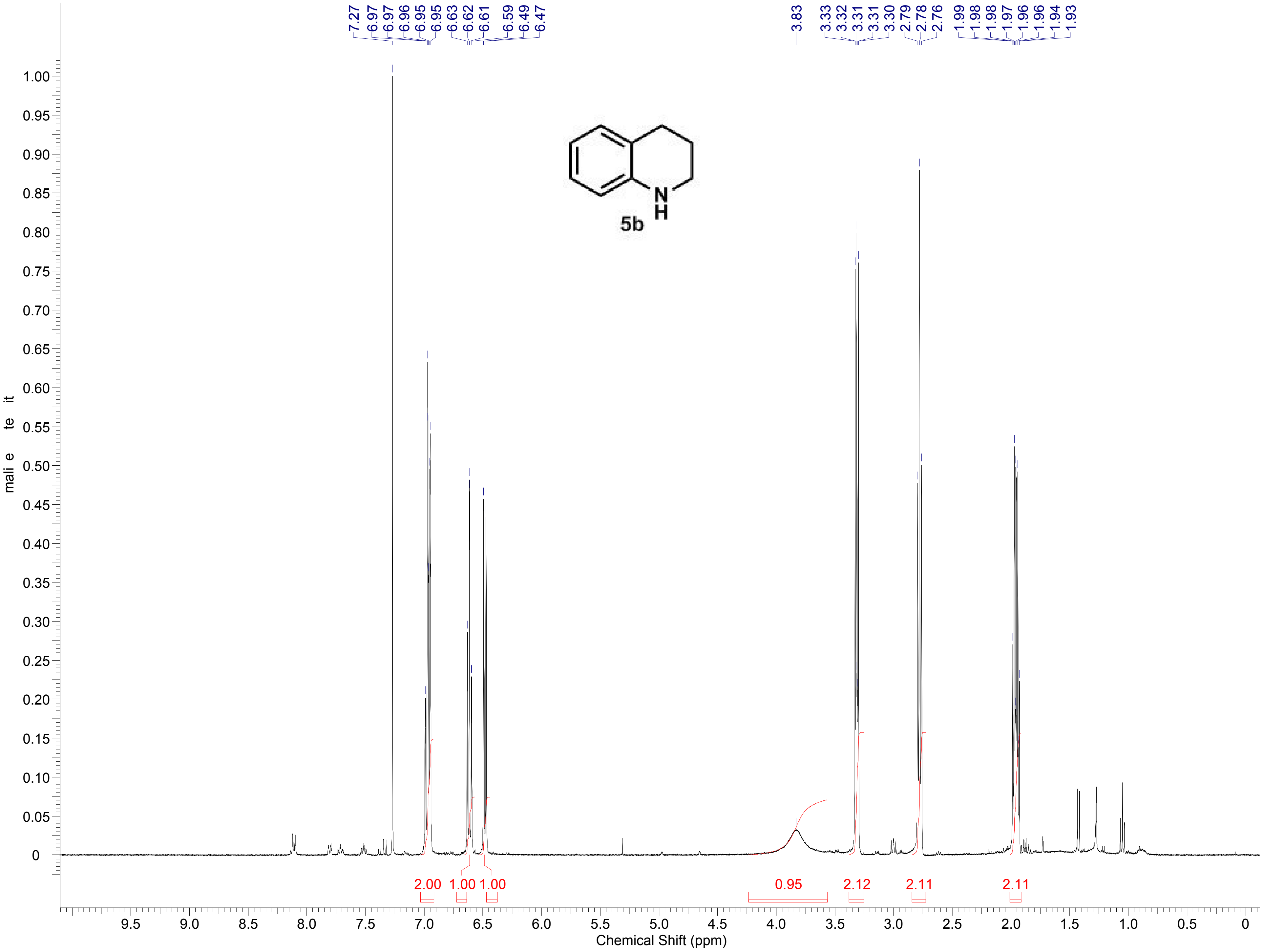

7.27 6.97 6.97 6.96 6.95 6.95 6.63 6.62 6.61 6.59 6.49 6.47
3.83
3.33 3.32 3.31 3.31 3.30 2.79 2.78 2.76
1.99 1.98 1.98 1.97 1.96 1.96 1.94 1.93
5b
N
H
2.00 1.00 1.00
0.95
2.12
2.11
2.11
Chemical Shift (ppm)

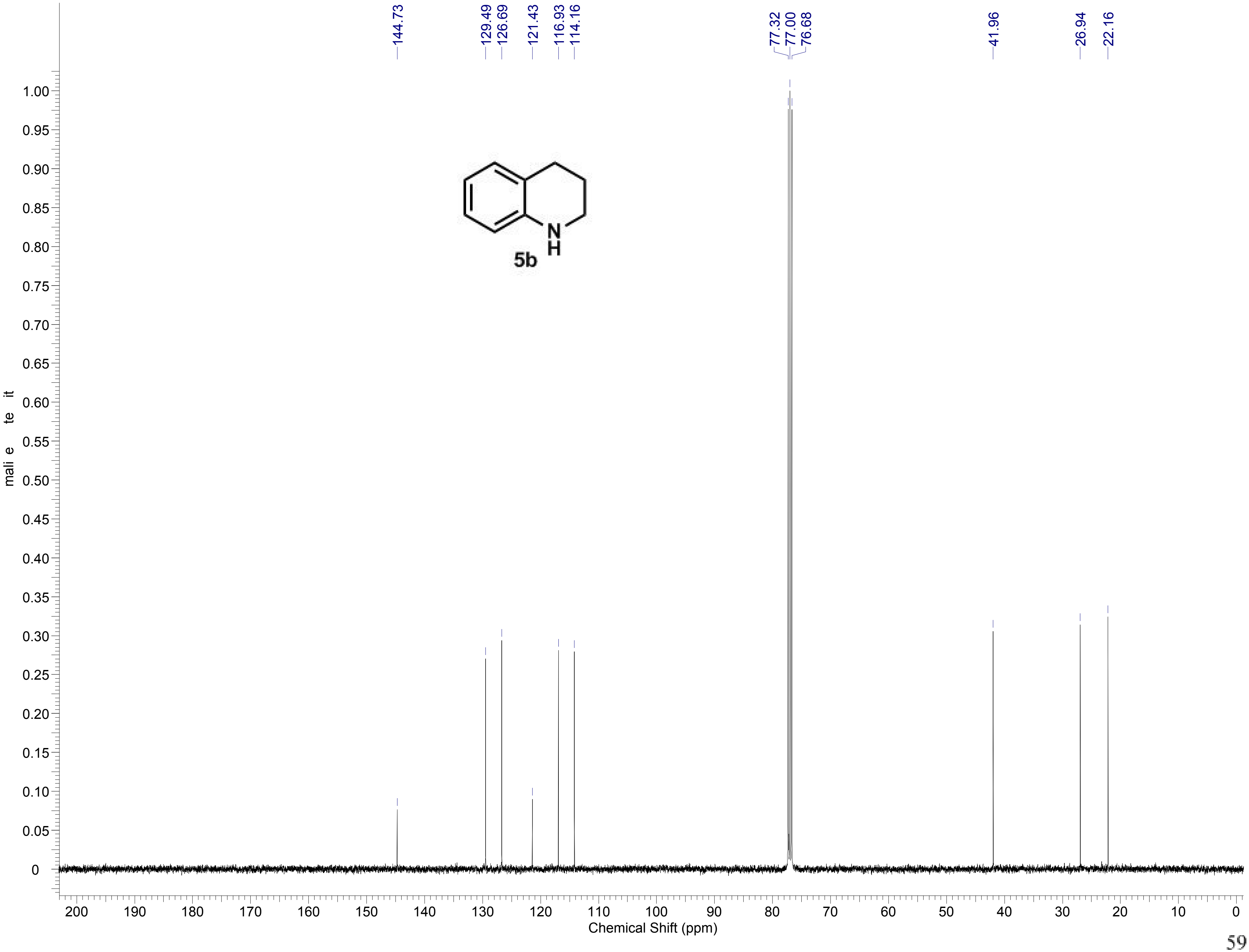
144.73
129.49
126.69
121.43
116.93
114.16
77.32
77.00
76.68
41.96
26.94
22.16
5b
N
H
1.00
0.95
0.90
0.85
0.80
0.75
0.70
0.65
0.60
0.55
0.50
0.45
0.40
0.35
0.30
0.25
0.20
0.15
0.10
0.05
0
200 190 180 170 160 150 140 130 120 110 100 90 80 70 60 50 40 30 20 10 0
Chemical Shift (ppm)

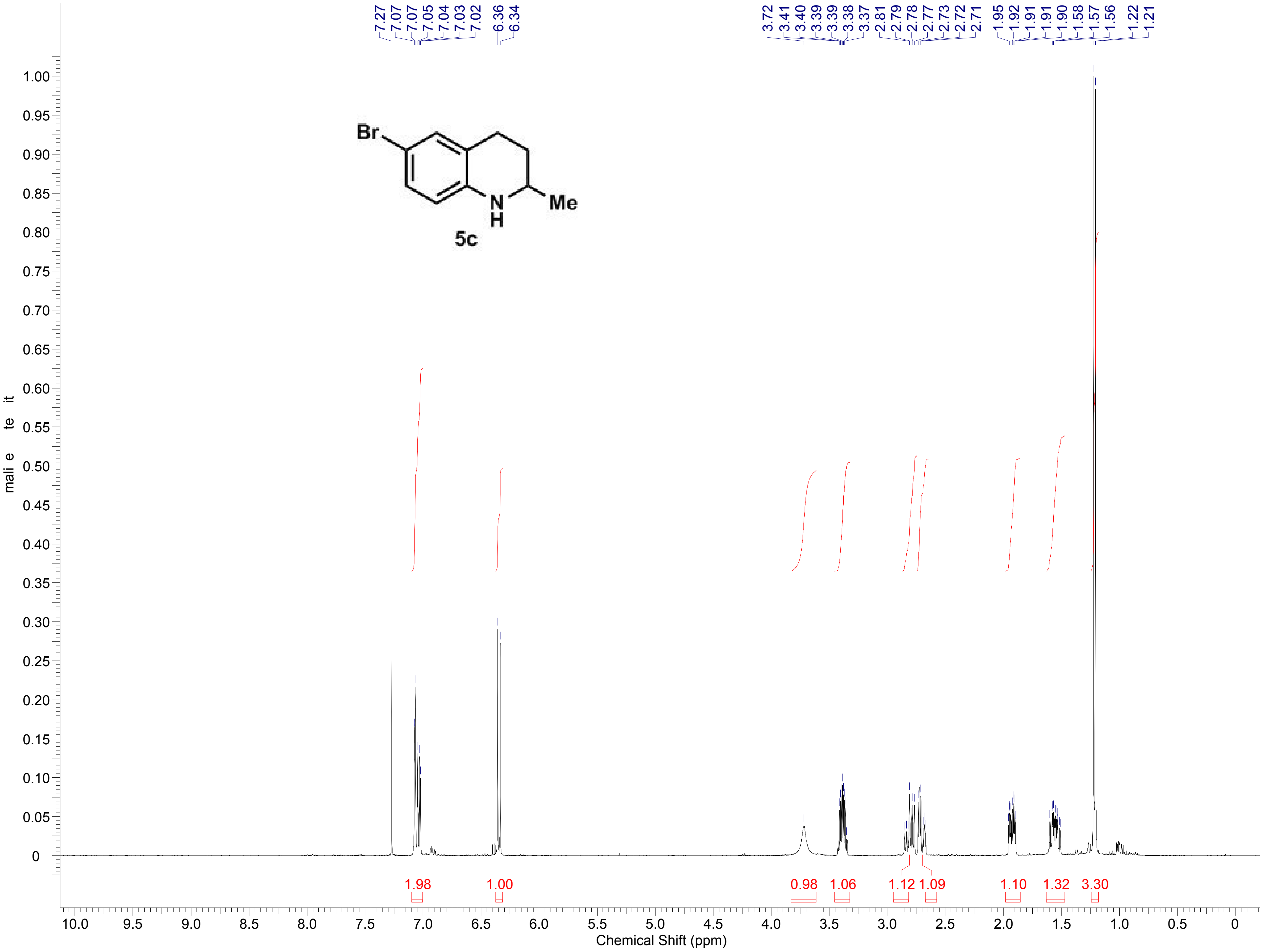

7.27 7.07 7.07 7.05 7.04 7.03 7.02 6.36 6.34
3.72 3.41 3.40 3.39 3.39 3.38 3.37 2.81 2.79 2.78 2.77 2.73 2.72 2.71 1.95 1.92 1.91 1.91 1.90 1.58 1.57 1.56 1.22 1.21
Br
N
H
Me
5c
1.98 1.00 0.98 1.06 1.12 1.09 1.10 1.32 3.30
Chemical Shift (ppm)

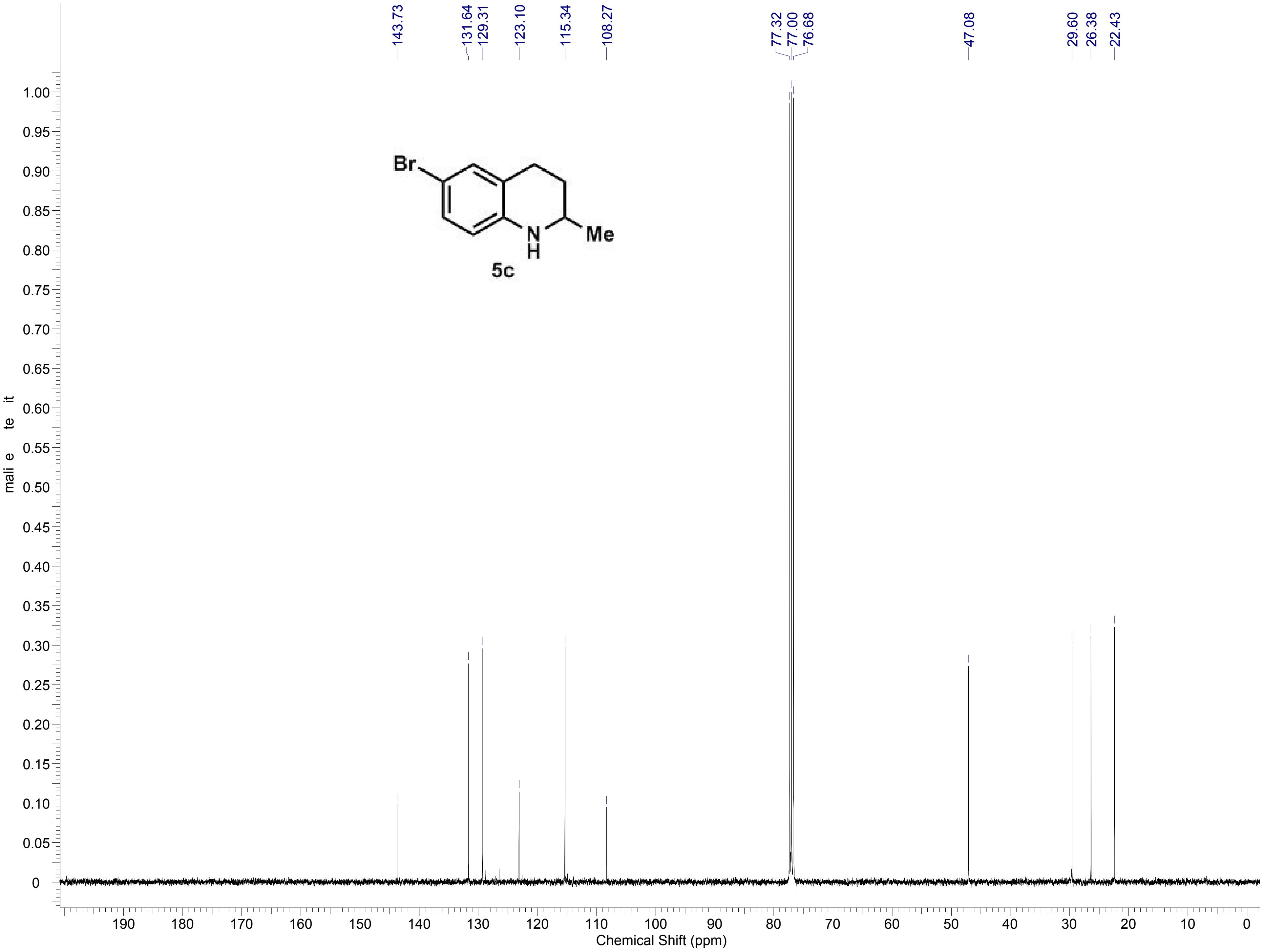
143.73
131.64
129.31
123.10
115.34
108.27
77.32
77.00
76.68
47.08
29.60
26.38
22.43
Br
N
H
Me
5c
mali e te it
1.00
0.95
0.90
0.85
0.80
0.75
0.70
0.65
0.60
0.55
0.50
0.45
0.40
0.35
0.30
0.25
0.20
0.15
0.10
0.05
0
190 180 170 160 150 140 130 120 110 100 90 80 70 60 50 40 30 20 10 0
Chemical Shift (ppm)

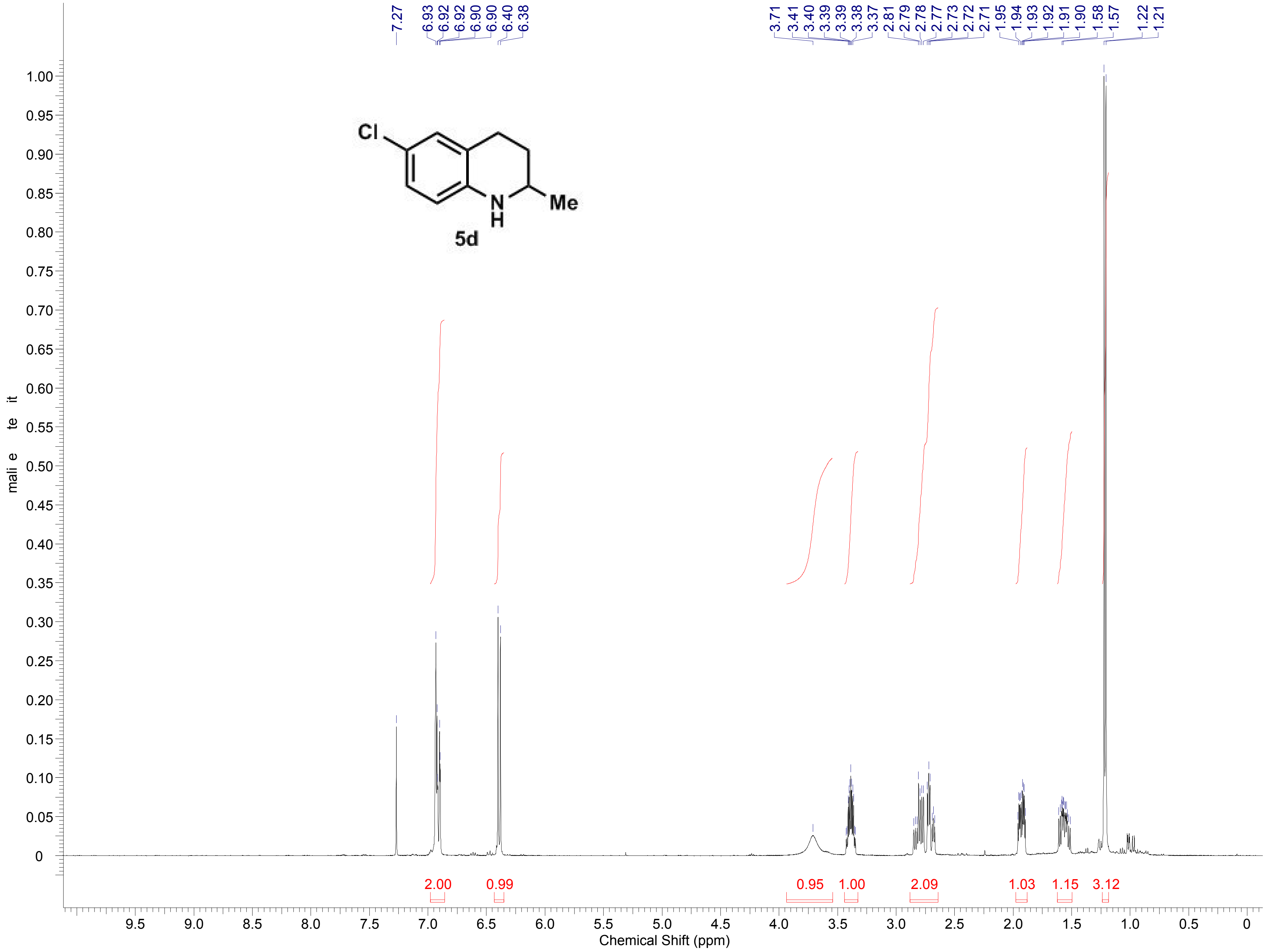
7.27
6.93
6.92
6.92
6.90
6.90
6.40
6.38
3.71
3.41
3.40
3.39
3.39
3.38
3.37
2.81
2.79
2.78
2.77
2.73
2.72
2.71
1.95
1.94
1.93
1.92
1.91
1.90
1.58
1.57
1.22
1.21
Cl
N
H
Me
5d
2.00
0.99
0.95
1.00
2.09
1.03
1.15
3.12
Chemical Shift (ppm)

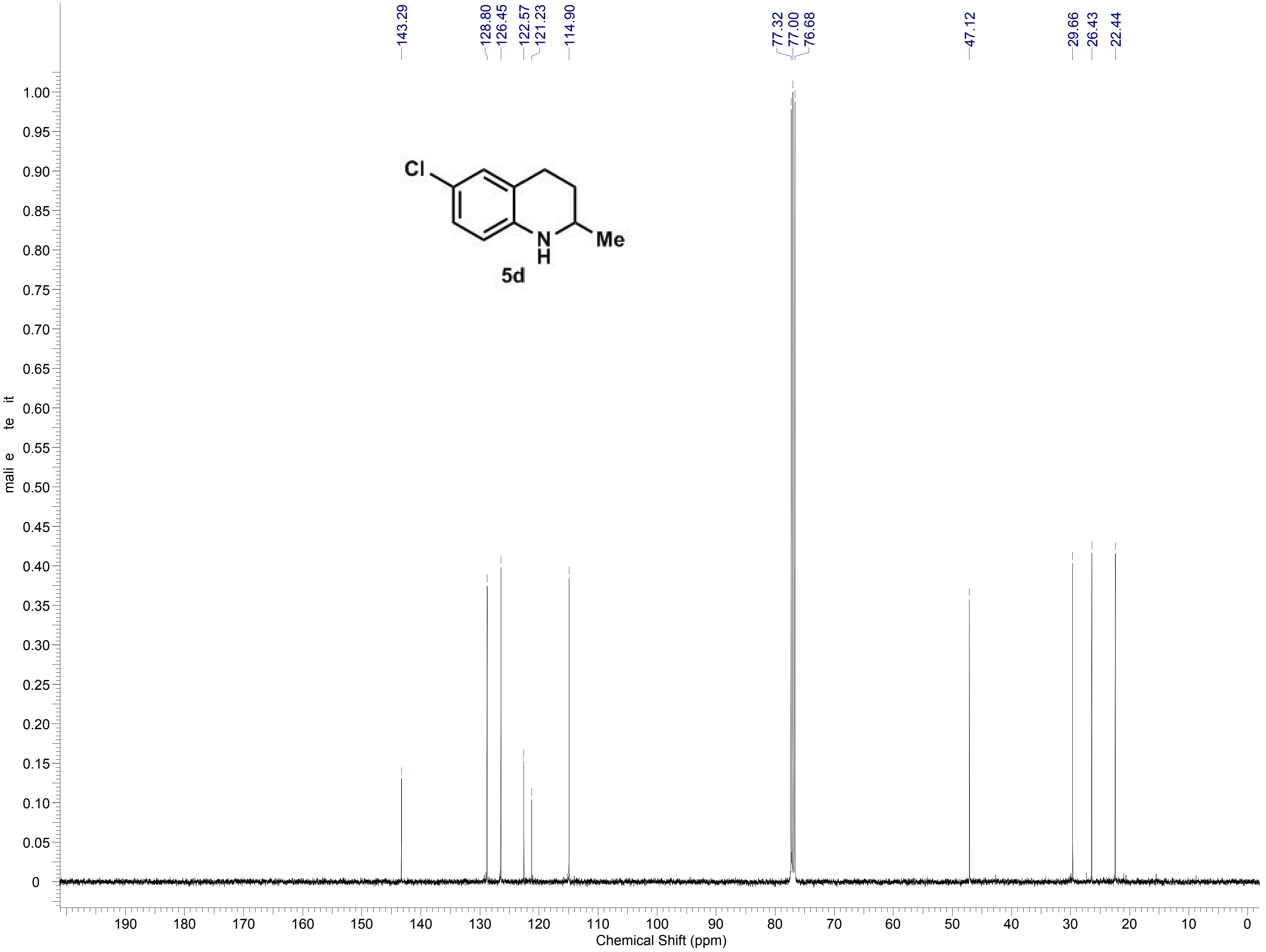
143.29
128.80
126.45
122.57
121.23
114.90
77.32
77.00
76.68
47.12
29.66
26.43
22.44
Cl
N
H
Me
5d
Chemical Shift (ppm)

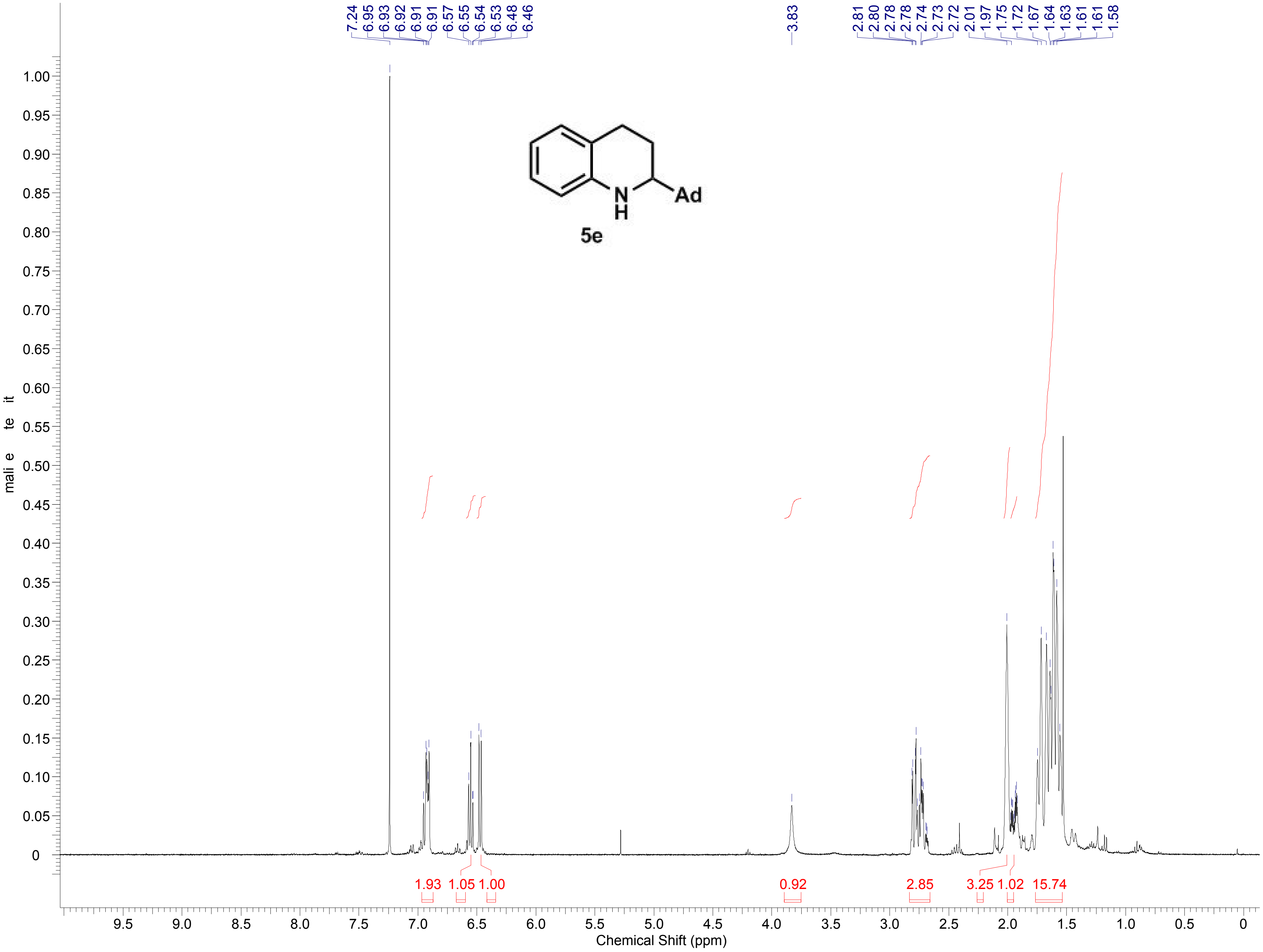

7.24
6.95
6.93
6.92
6.91
6.91
6.57
6.55
6.54
6.53
6.48
6.46
3.83
2.81
2.80
2.78
2.78
2.74
2.73
2.72
2.01
1.97
1.75
1.72
1.67
1.64
1.63
1.61
1.61
1.58
N
H
Ad
5e
1.93
1.05
1.00
0.92
2.85
3.25
1.02
15.74
Chemical Shift (ppm)

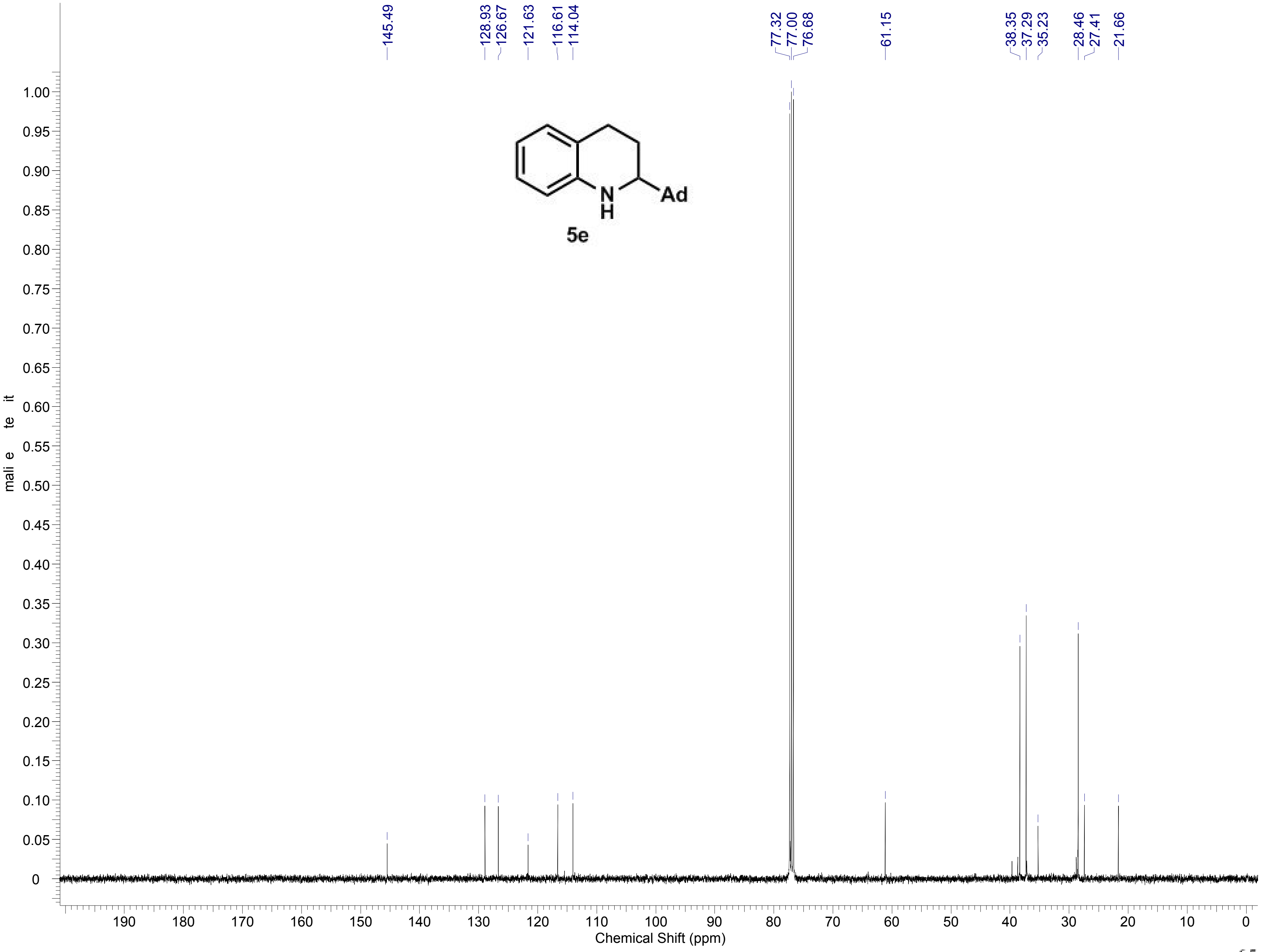
145.49
128.93
126.67
121.63
116.61
114.04
77.32
77.00
76.68
61.15
38.35
37.29
35.23
28.46
27.41
21.66
N
H
Ad
5e
1.00
0.95
0.90
0.85
0.80
0.75
0.70
0.65
0.60
0.55
0.50
0.45
0.40
0.35
0.30
0.25
0.20
0.15
0.10
0.05
0
190
180
170
160
150
140
130
120
110
100
90
80
70
60
50
40
30
20
10
0
Chemical Shift (ppm)

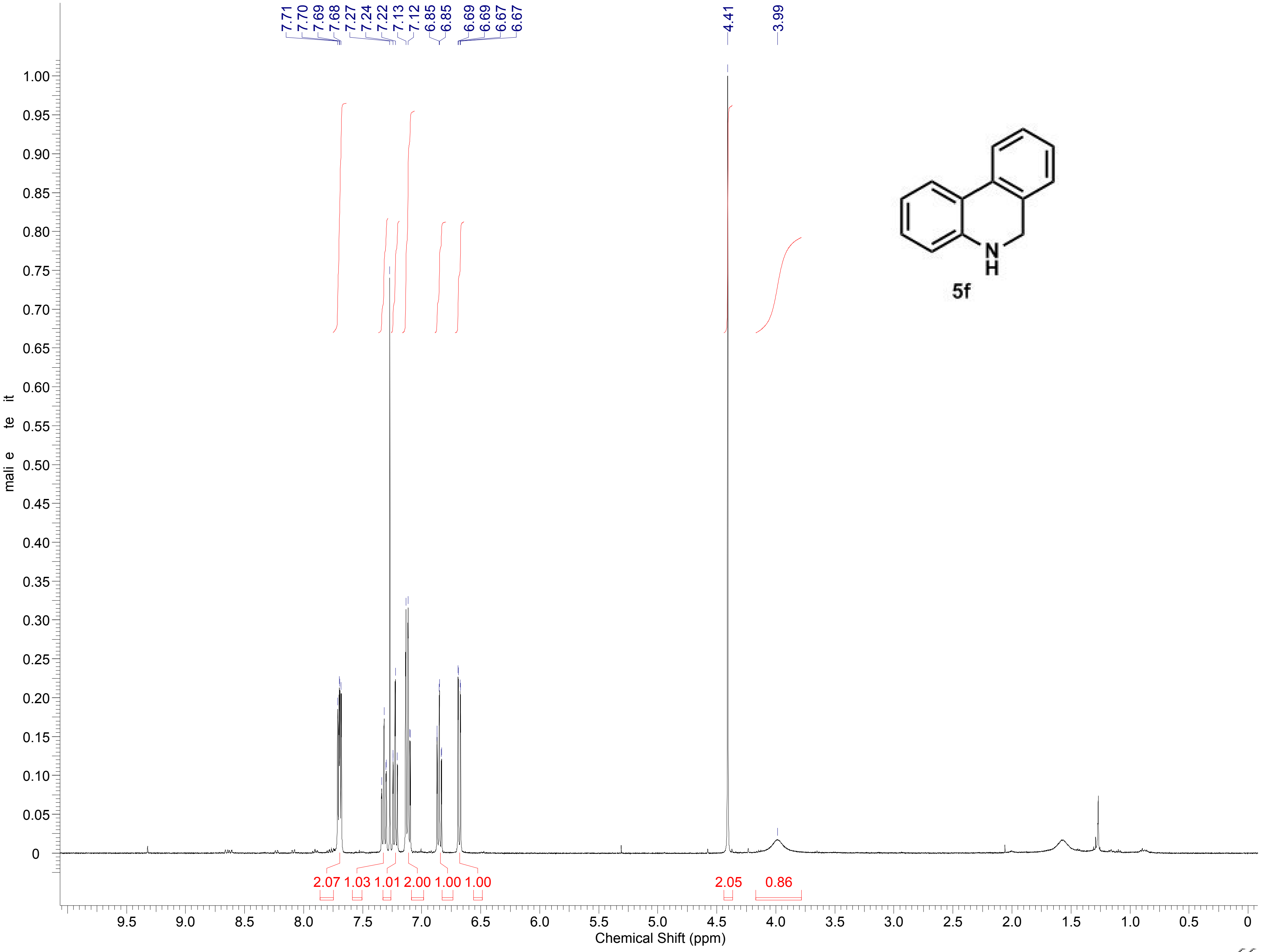
7.71
7.70
7.69
7.68
7.27
7.24
7.22
7.13
7.12
6.85
6.85
6.69
6.69
6.67
6.67
4.41
3.99
5f
2.07 1.03 1.01 2.00 1.00 1.00
2.05
0.86
Chemical Shift (ppm)

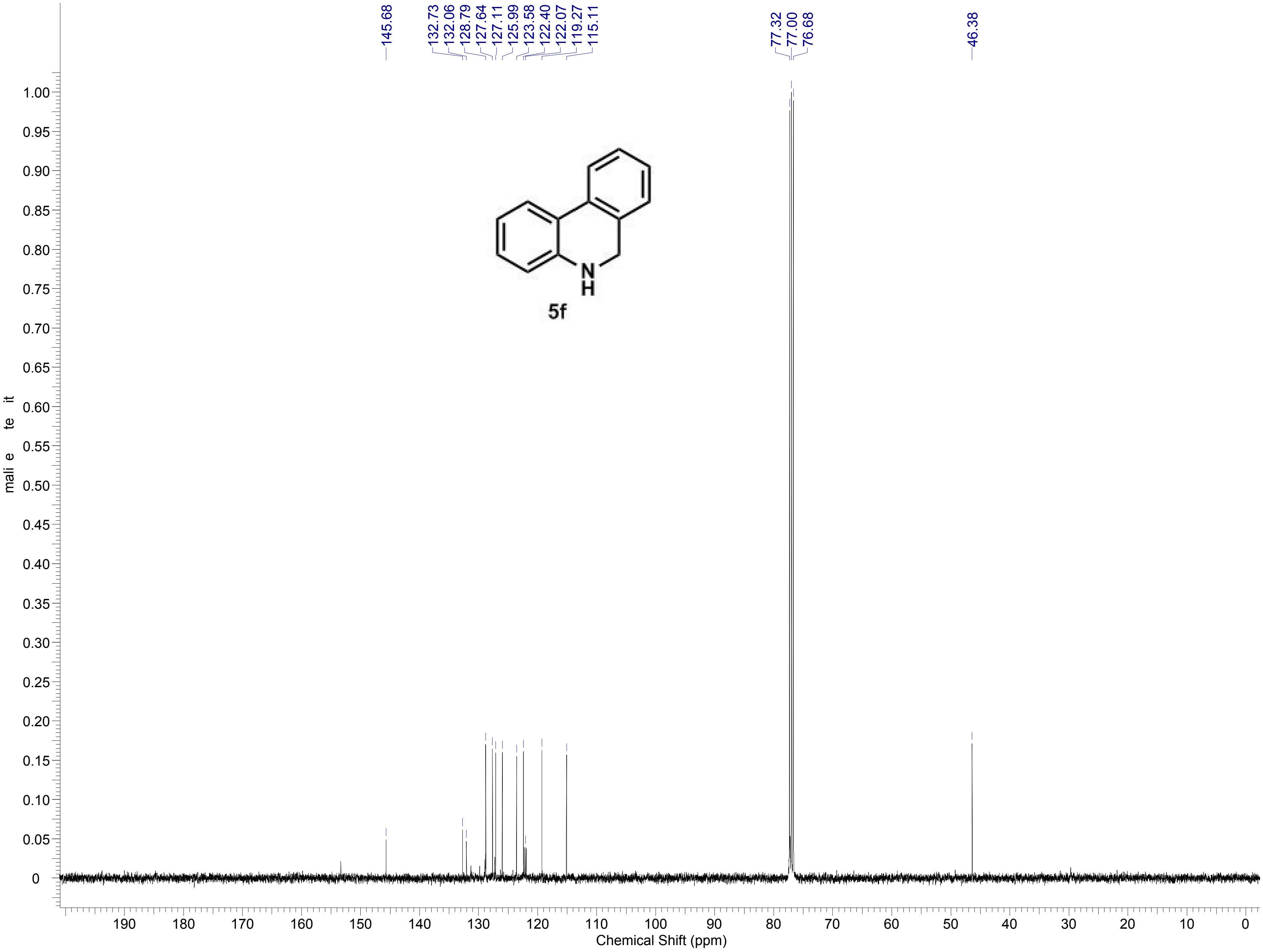
145.68
132.73
132.06
128.79
127.64
127.11
125.99
123.58
122.40
122.07
119.27
115.11
77.32
77.00
76.68
46.38
N
H
5f
Chemical Shift (ppm)

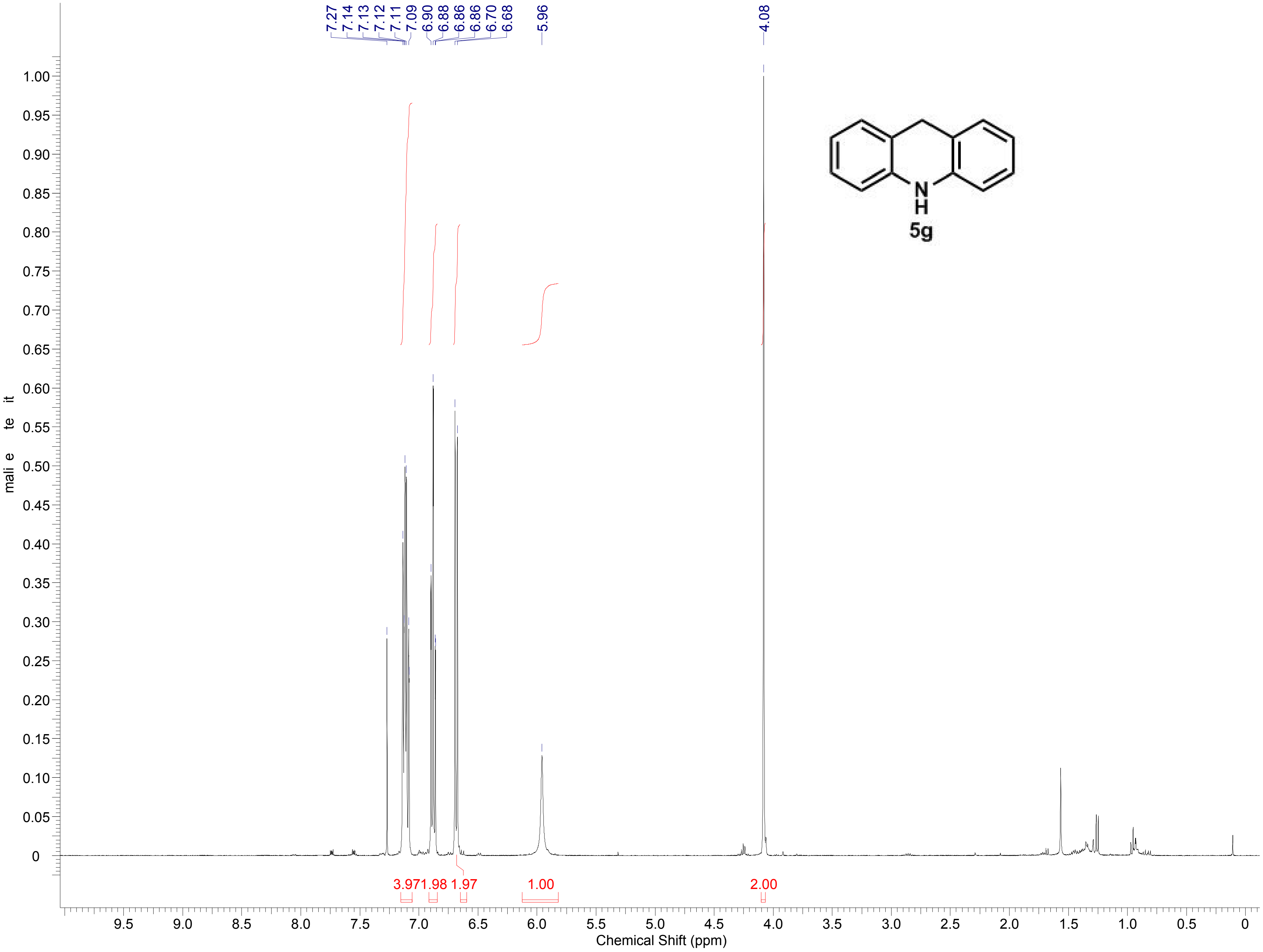
7.27
7.14
7.13
7.12
7.11
7.09
6.90
6.88
6.86
6.86
6.70
6.68
5.96
4.08
N
H
5g
3.97
1.98
1.97
1.00
2.00
Chemical Shift (ppm)

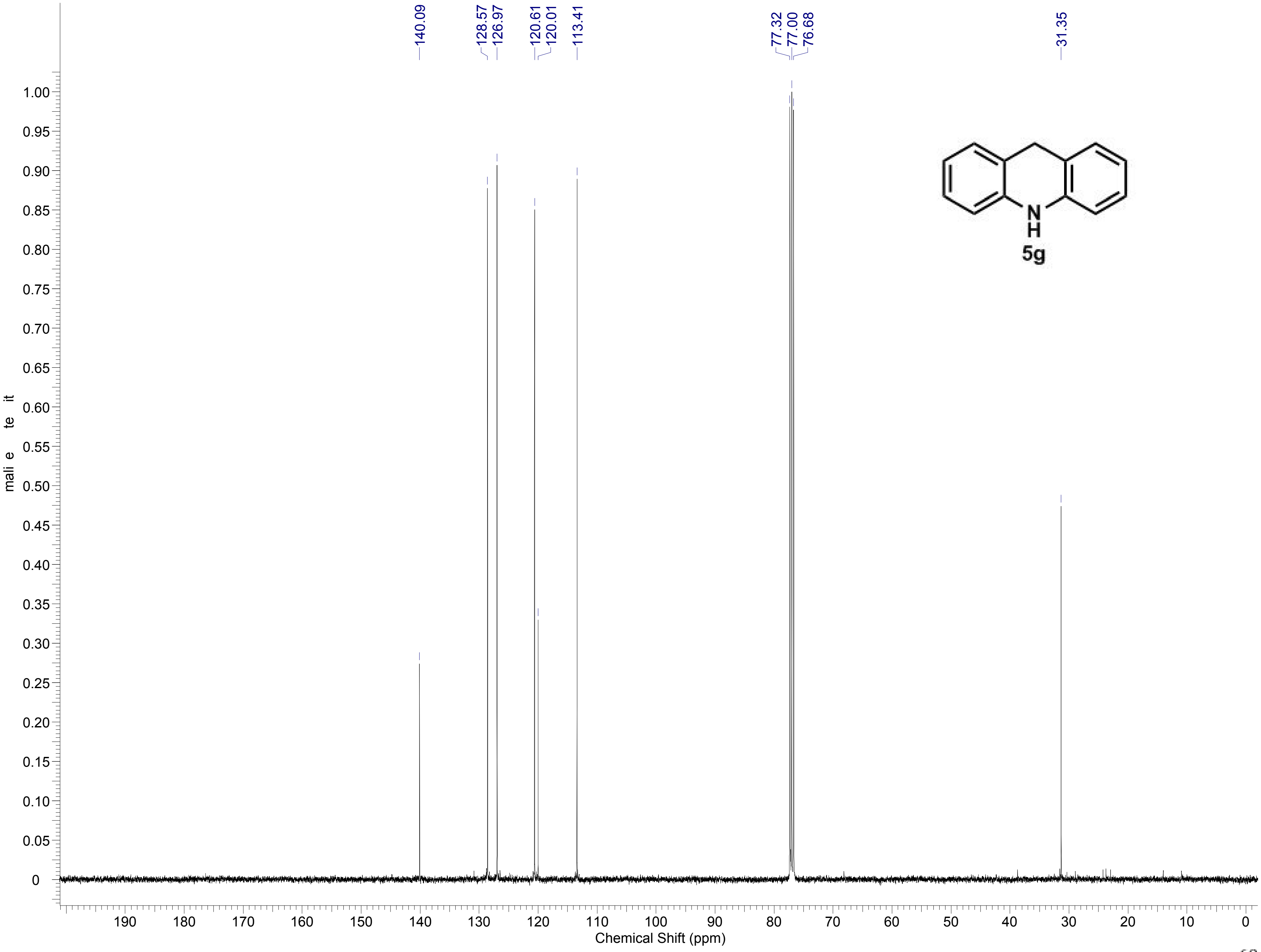
140.09
128.57
126.97
120.61
120.01
113.41
77.32
77.00
76.68
31.35
N
H
5g
Chemical Shift (ppm)

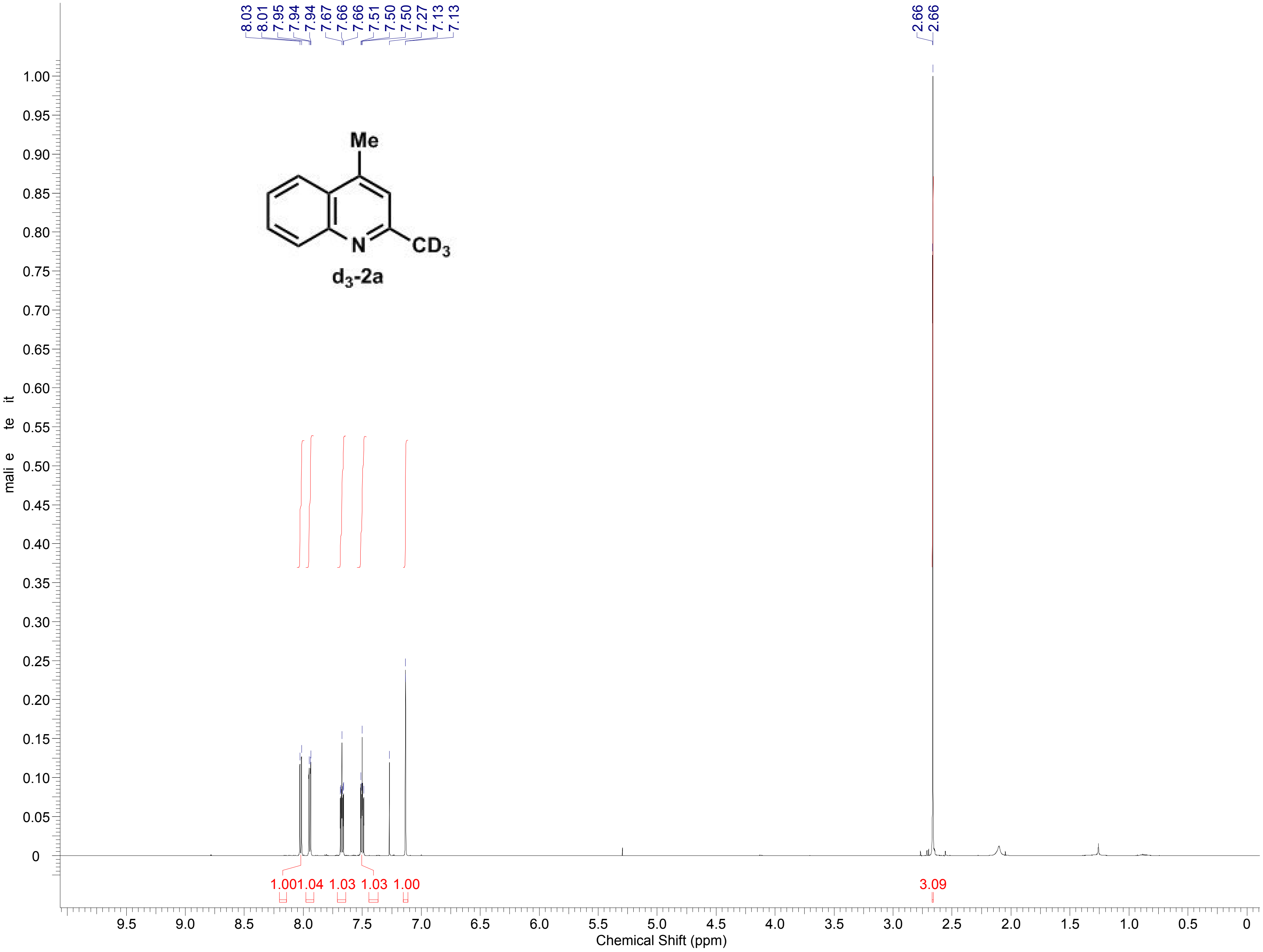
8.03
8.01
7.95
7.94
7.94
7.67
7.66
7.66
7.51
7.50
7.50
7.27
7.13
7.13
2.66
2.66
Me
N
CD3
d3-2a
1.00
1.04
1.03
1.03
1.00
3.09
Chemical Shift (ppm)

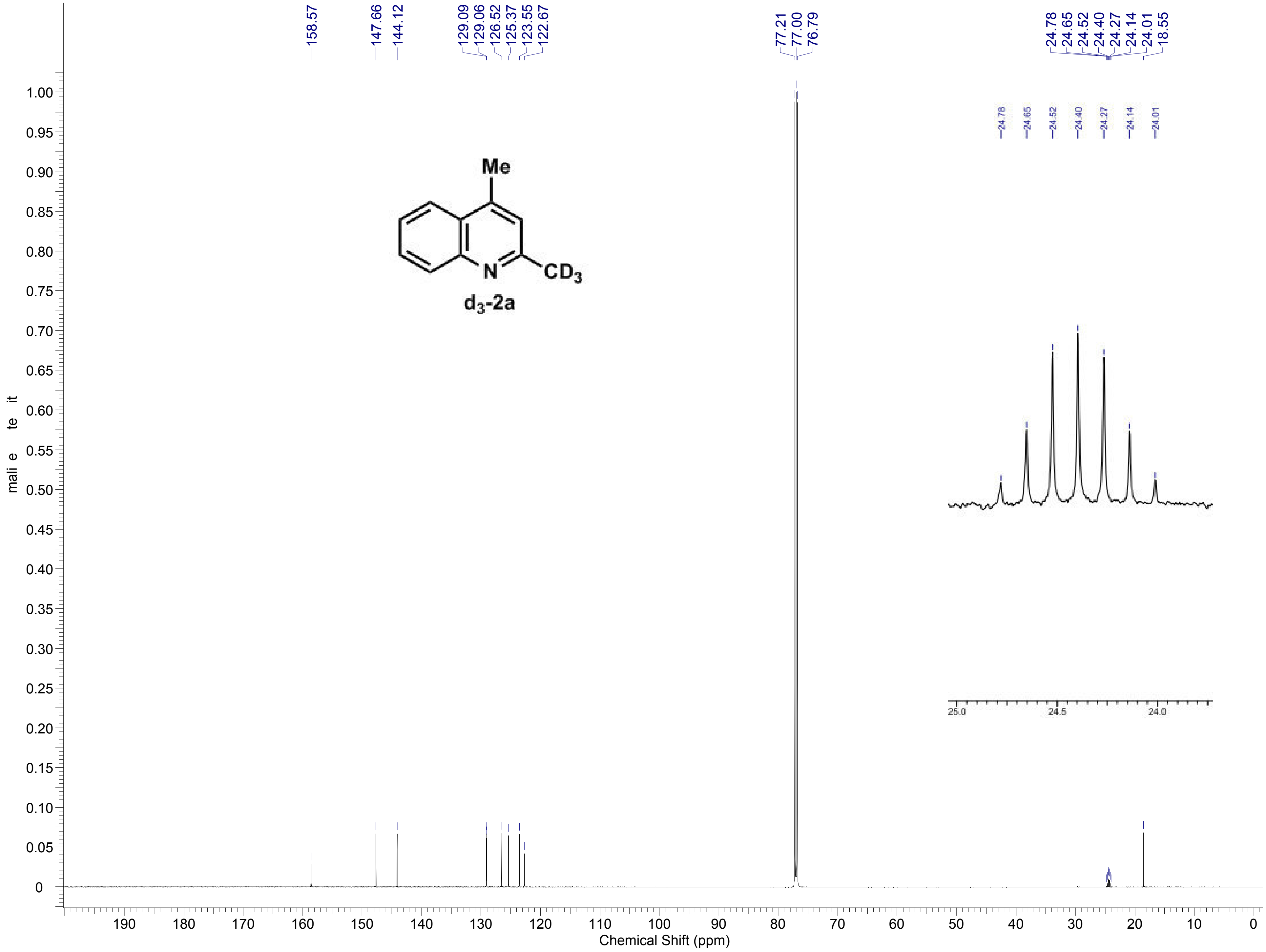

158.57
147.66
144.12
129.09
129.06
126.52
125.37
123.55
122.67
77.21
77.00
76.79
24.78
24.65
24.52
24.40
24.27
24.14
24.01
18.55
Me
N
CD3
d3-2a
Chemical Shift (ppm)

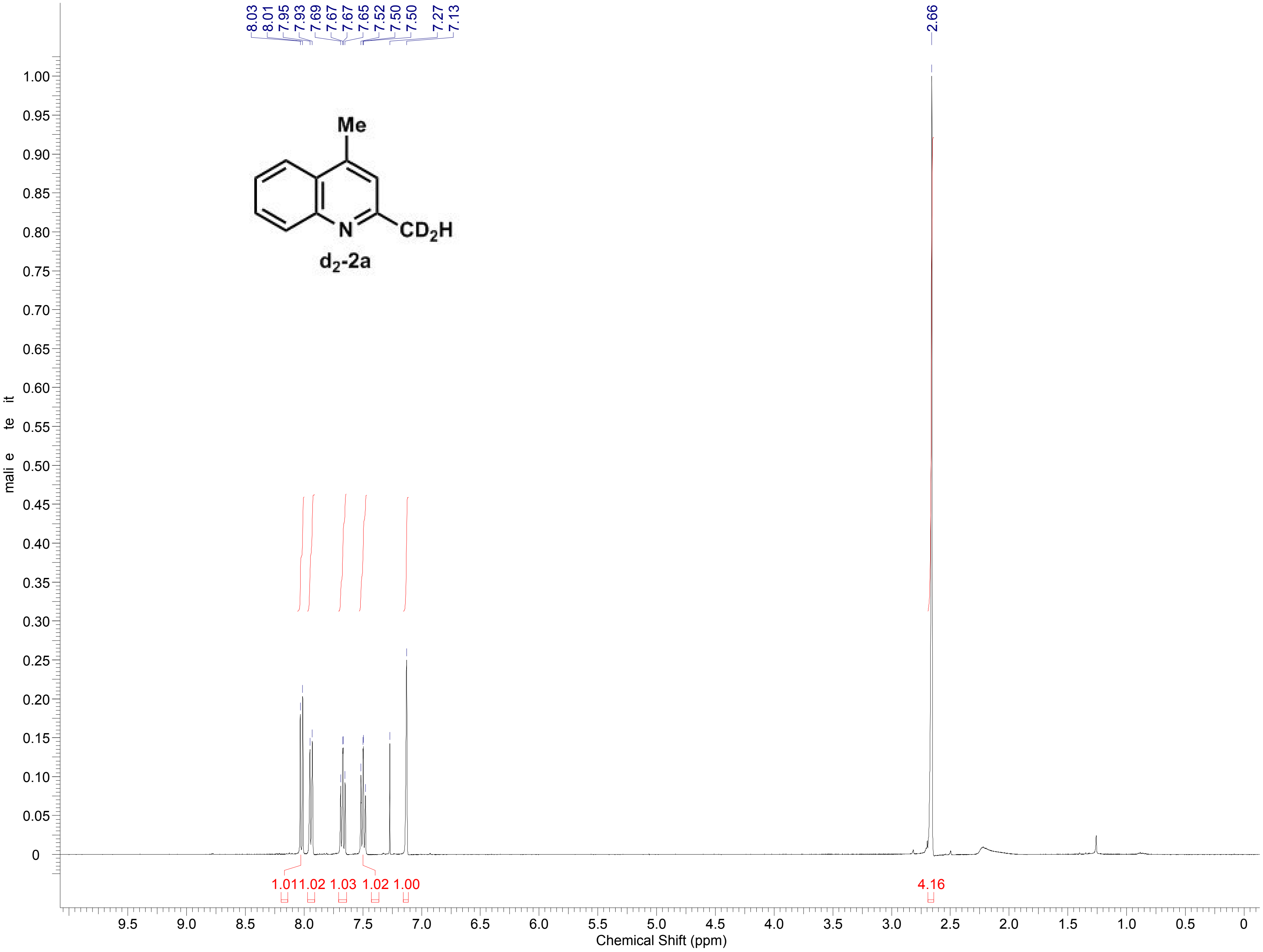
8.03
8.01
7.95
7.93
7.69
7.67
7.67
7.65
7.52
7.50
7.50
7.27
7.13
2.66
Me
N
CD2H
d2-2a
1.01
1.02
1.03
1.02
1.00
4.16
Chemical Shift (ppm)

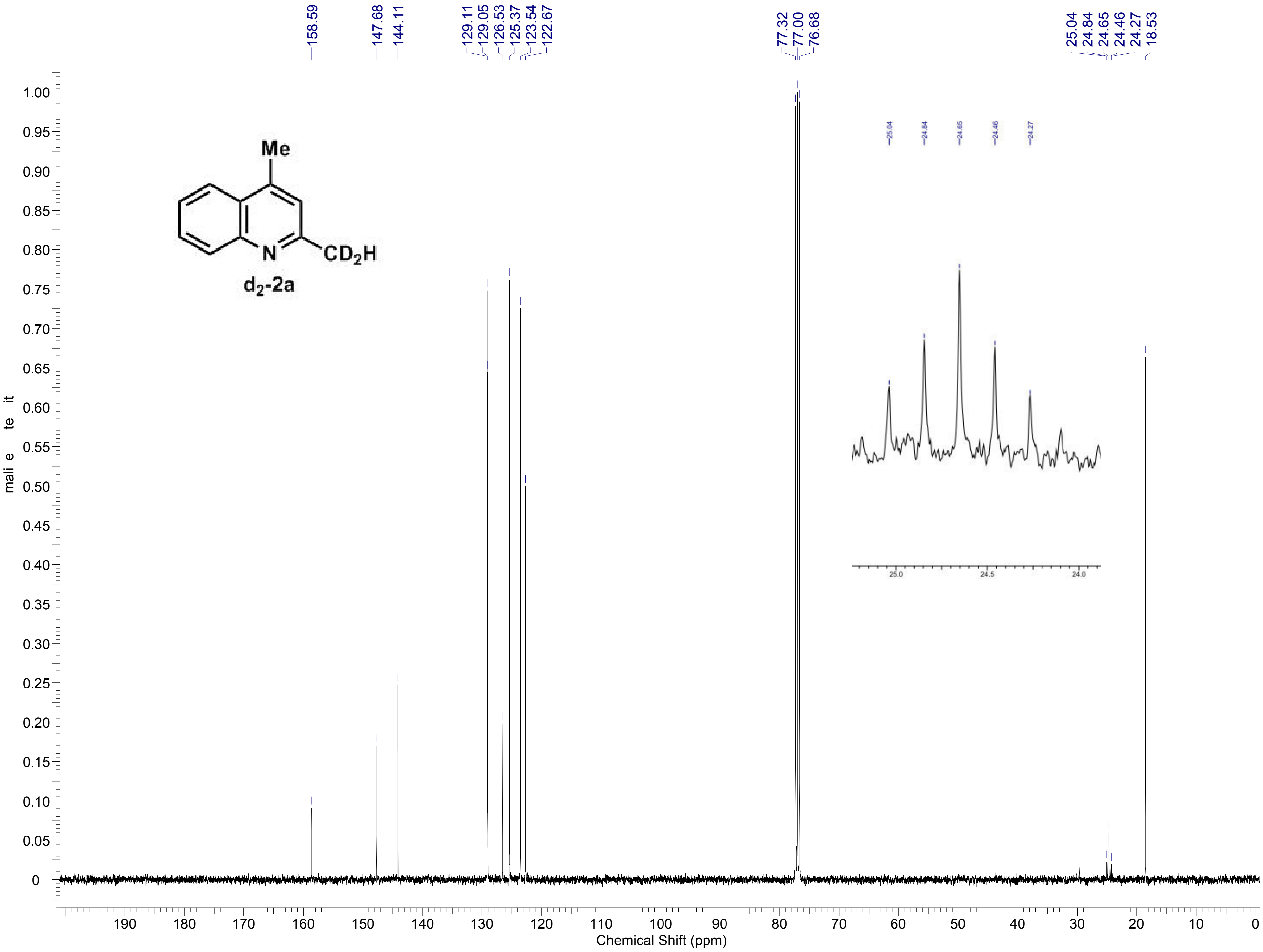

158.59
147.68
144.11
129.11
129.05
126.53
125.37
123.54
122.67
77.32
77.00
76.68
25.04
24.84
24.65
24.46
24.27
18.53
Me
N
CD2H
d2-2a
Chemical Shift (ppm)

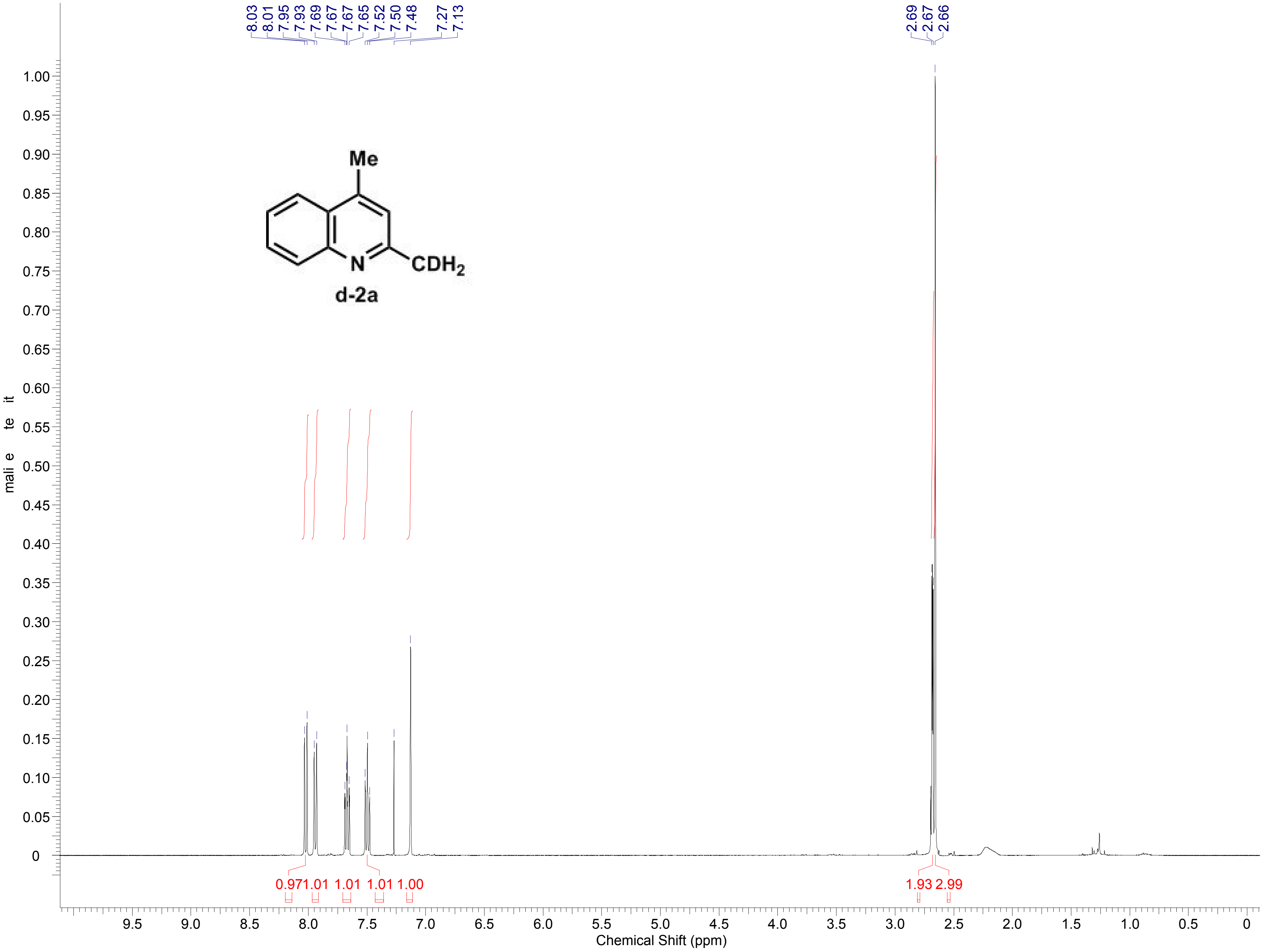
8.03
8.01
7.95
7.93
7.69
7.67
7.67
7.65
7.52
7.50
7.48
7.27
7.13
2.69
2.67
2.66
Me
N
CDH2
d-2a
0.97
1.01
1.01
1.01
1.00
1.93
2.99
Chemical Shift (ppm)

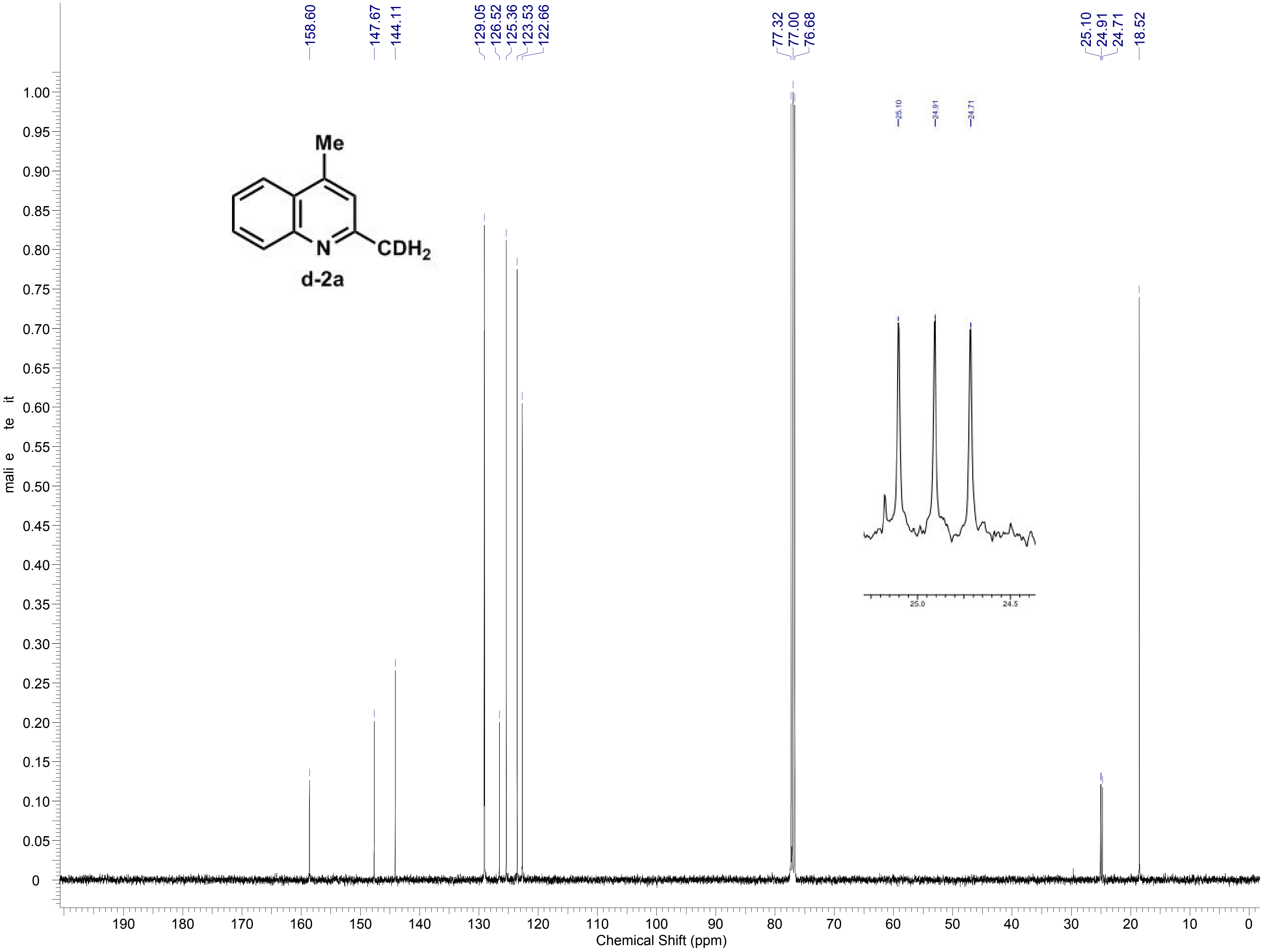
158.60
147.67
144.11
129.05
126.52
125.36
123.53
122.66
77.32
77.00
76.68
25.10
24.91
24.71
18.52
Me
N
CDH2
d-2a
Chemical Shift (ppm)

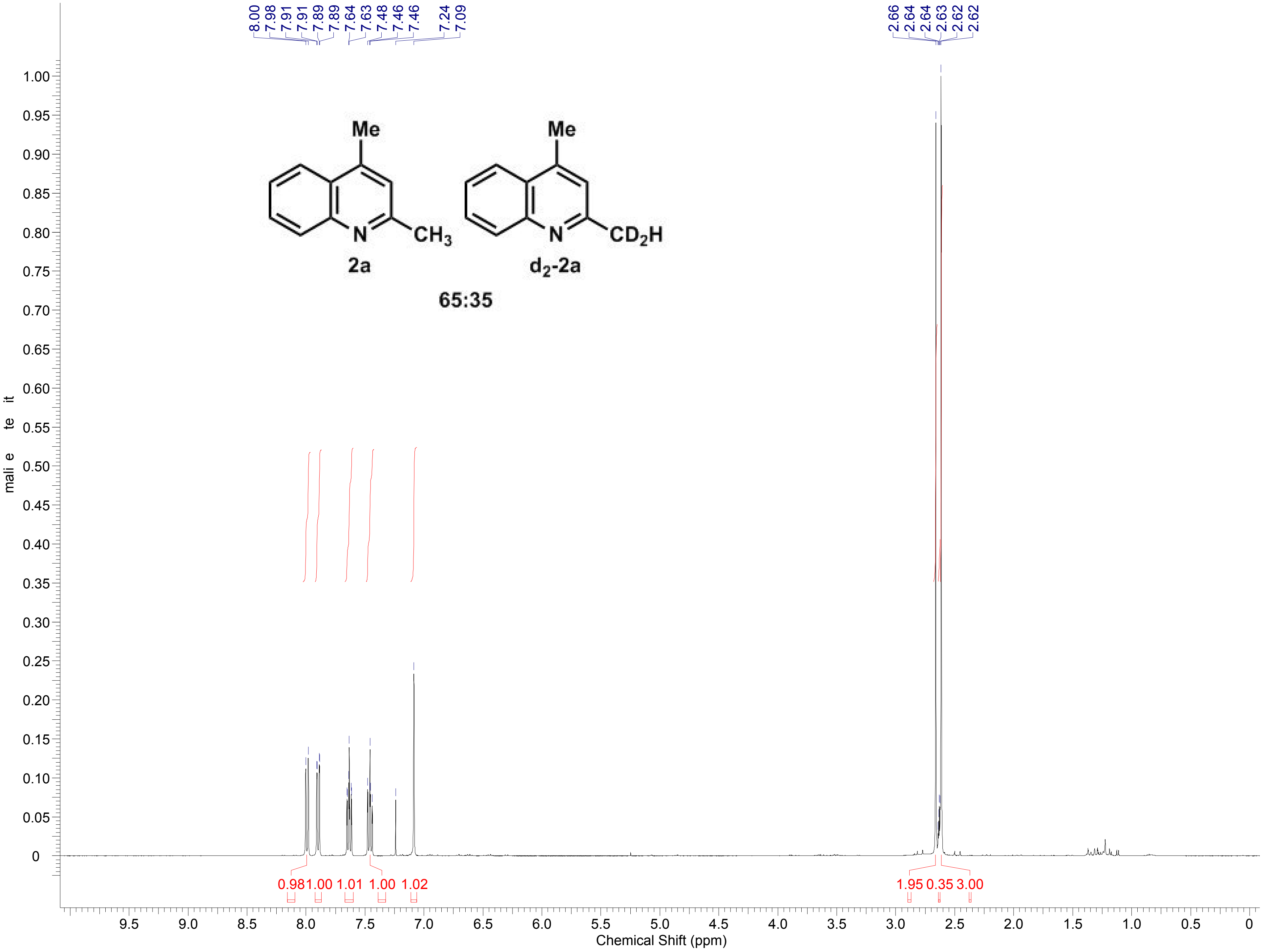

8.00 7.98 7.91 7.91 7.89 7.89 7.64 7.63 7.48 7.46 7.46 7.24 7.09
2.66 2.64 2.64 2.63 2.62 2.62
Me
N
CH3
2a
Me
N
CD2H
d2-2a
65:35
0.98 1.00 1.01 1.00 1.02
1.95 0.35 3.00
Chemical Shift (ppm)

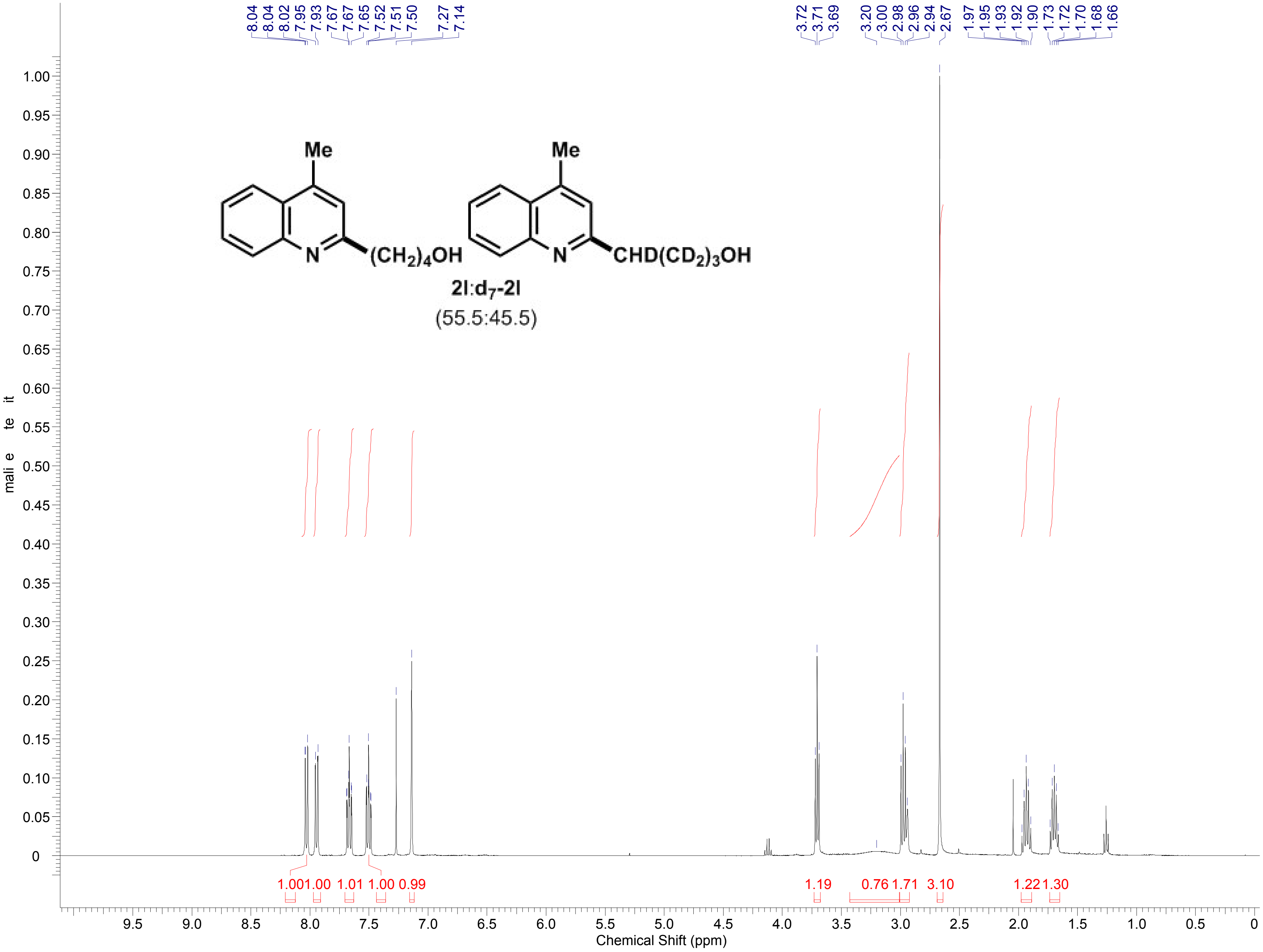
8.04 8.04 8.02 7.95 7.93 7.67 7.67 7.65 7.52 7.51 7.50 7.27 7.14
3.72 3.71 3.69 3.20 3.00 2.98 2.96 2.94 2.67 1.97 1.95 1.93 1.92 1.90 1.73 1.72 1.70 1.68 1.66
Me
(CH2)4OH
CHD(CD2)3OH
N
2l:d7-2l
(55.5:45.5)
1.00 1.00 1.01 1.00 0.99 1.19 0.76 1.71 3.10 1.22 1.30
Chemical Shift (ppm)

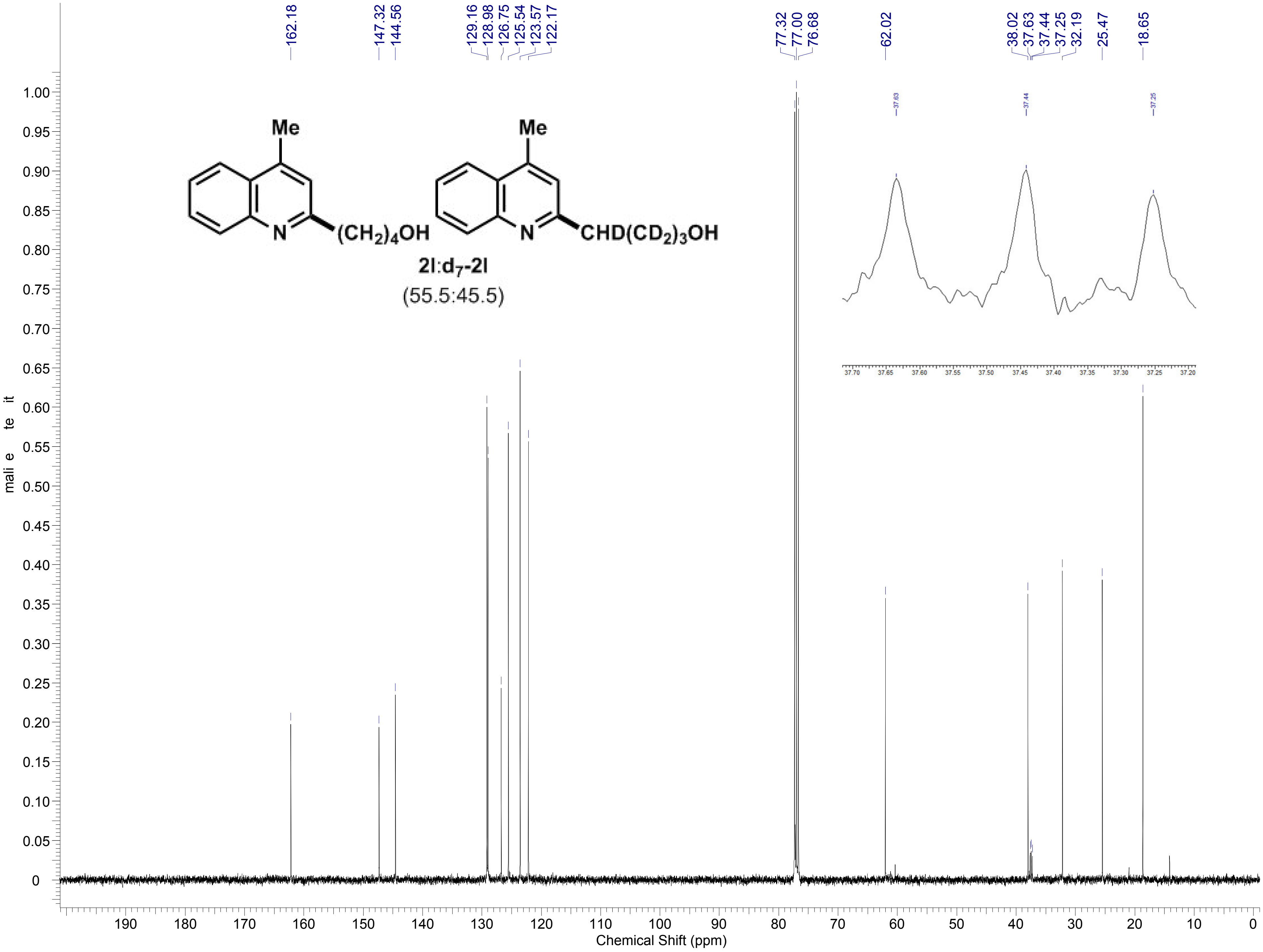
162.18
147.32
144.56
129.16
128.98
126.75
125.54
123.57
122.17
77.32
77.00
76.68
62.02
38.02
37.63
37.44
37.25
32.19
25.47
18.65
Me
N
(CH2)4OH
CHD(CD2)3OH
2l:d7-2l
(55.5:45.5)
Chemical Shift (ppm)

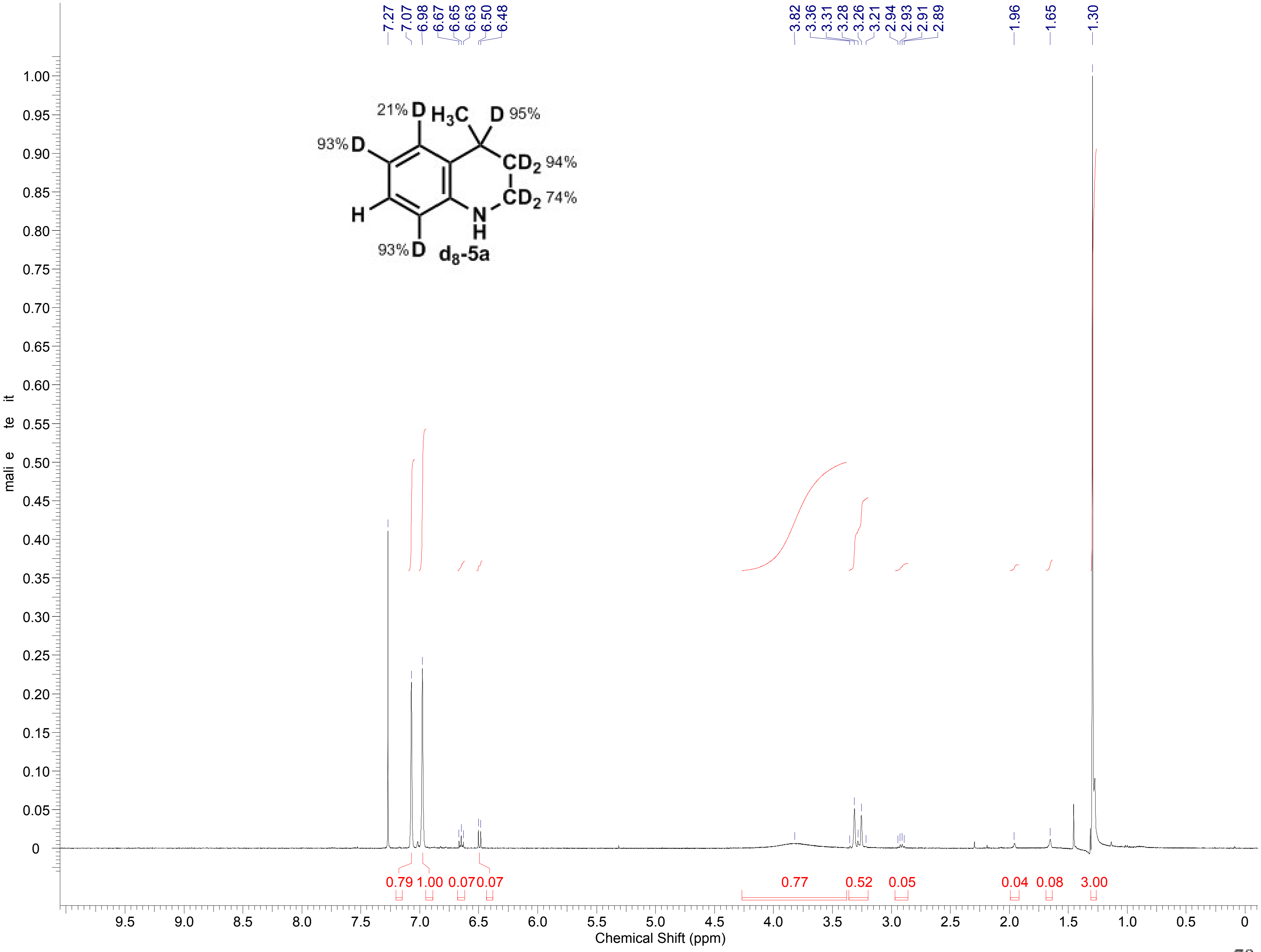
7.27
7.07
6.98
6.67
6.65
6.63
6.50
6.48
3.82
3.36
3.31
3.28
3.26
3.21
2.94
2.93
2.91
2.89
1.96
1.65
1.30
21% D
H_3C
D 95%
93% D
CD_2 94%
CD_2 74%
H
N
H
93% D
d_8-5a
0.79
1.00
0.07
0.07
0.77
0.52
0.05
0.04
0.08
3.00
Chemical Shift (ppm)

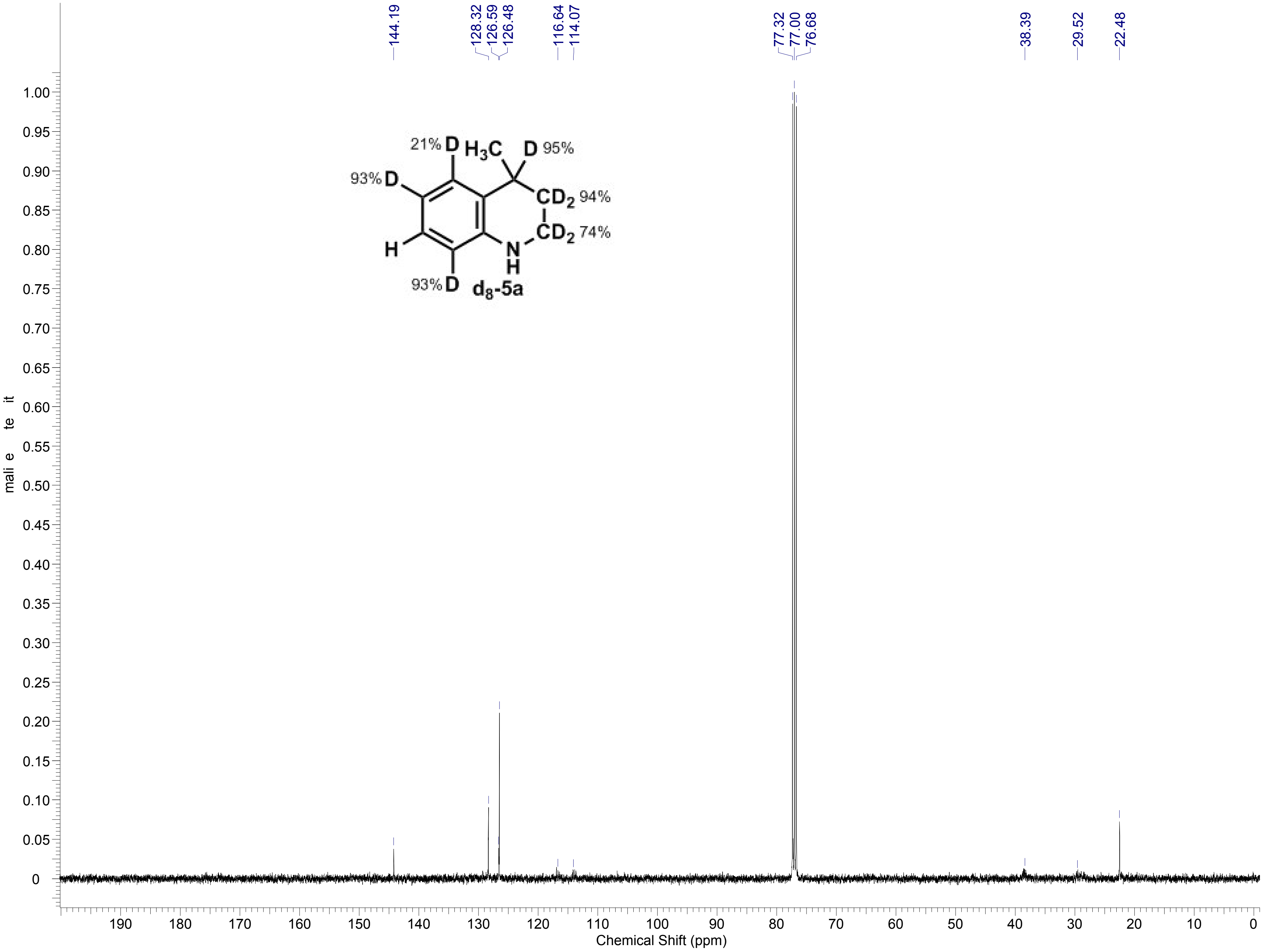

144.19
128.32
126.59
126.48
116.64
114.07
77.32
77.00
76.68
38.39
29.52
22.48
21% D
H3C
D 95%
93% D
CD2 94%
CD2 74%
H
N
H
93% D
d8-5a
Chemical Shift (ppm)

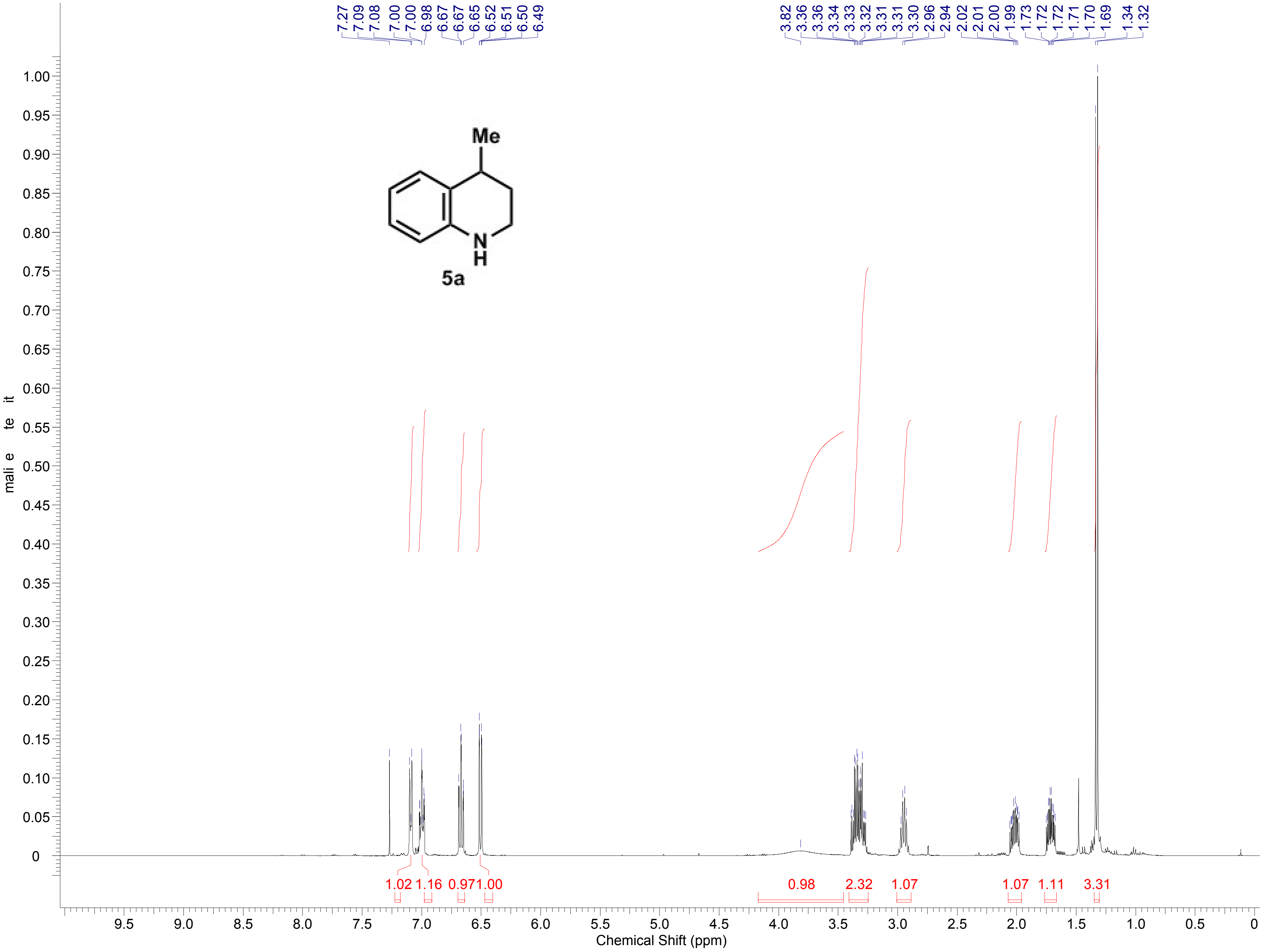
7.27
7.09
7.08
7.00
7.00
6.98
6.67
6.67
6.65
6.52
6.51
6.50
6.49
3.82
3.36
3.36
3.34
3.33
3.32
3.31
3.31
3.30
2.96
2.94
2.02
2.01
2.00
1.99
1.73
1.72
1.72
1.71
1.70
1.69
1.34
1.32
Me
N
H
5a
1.02
1.16
0.97
1.00
0.98
2.32
1.07
1.07
1.11
3.31
Chemical Shift (ppm)

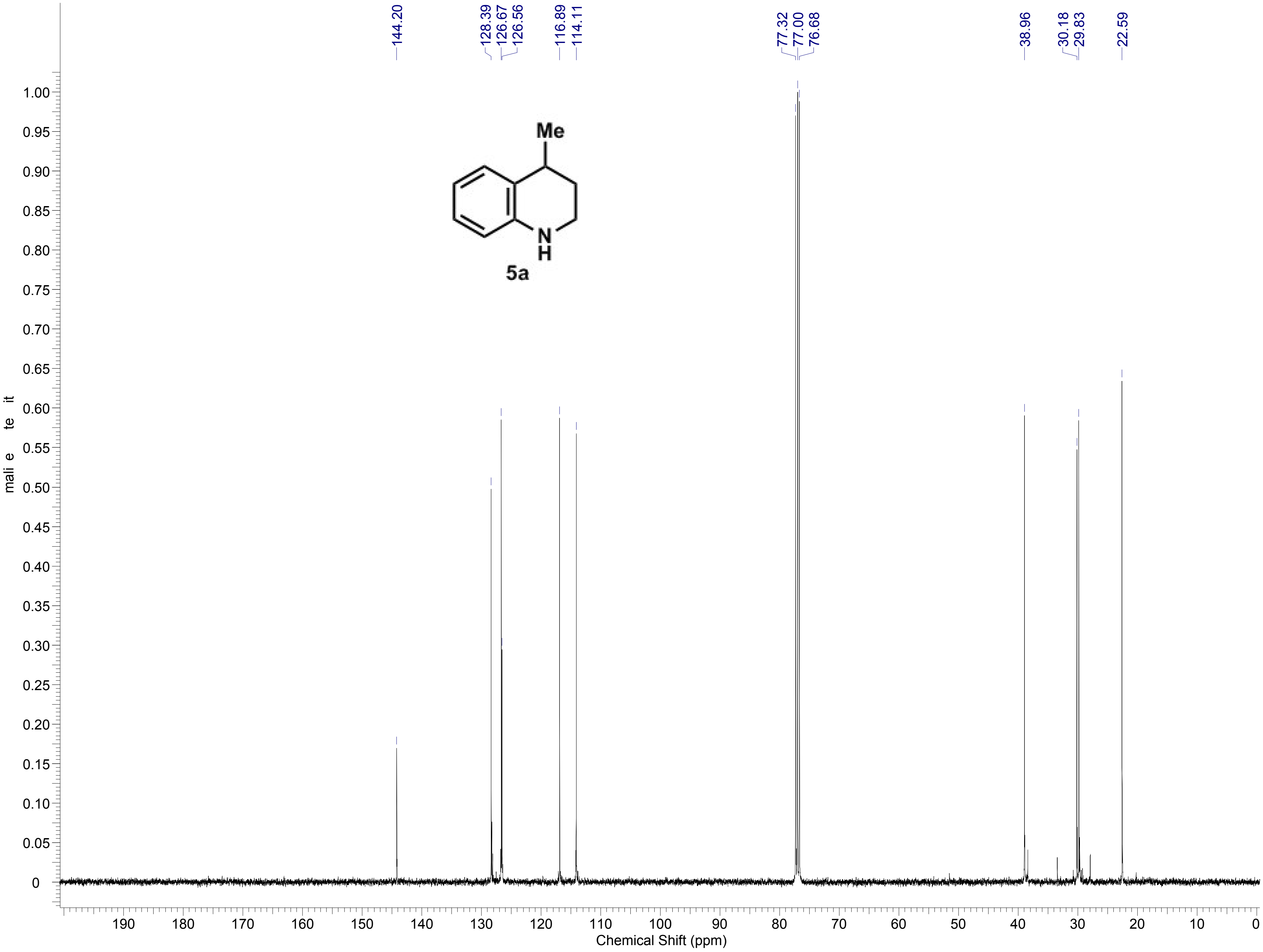
144.20
128.39
126.67
126.56
116.89
114.11
77.32
77.00
76.68
38.96
30.18
29.83
22.59
Me
N
H
5a
Chemical Shift (ppm)

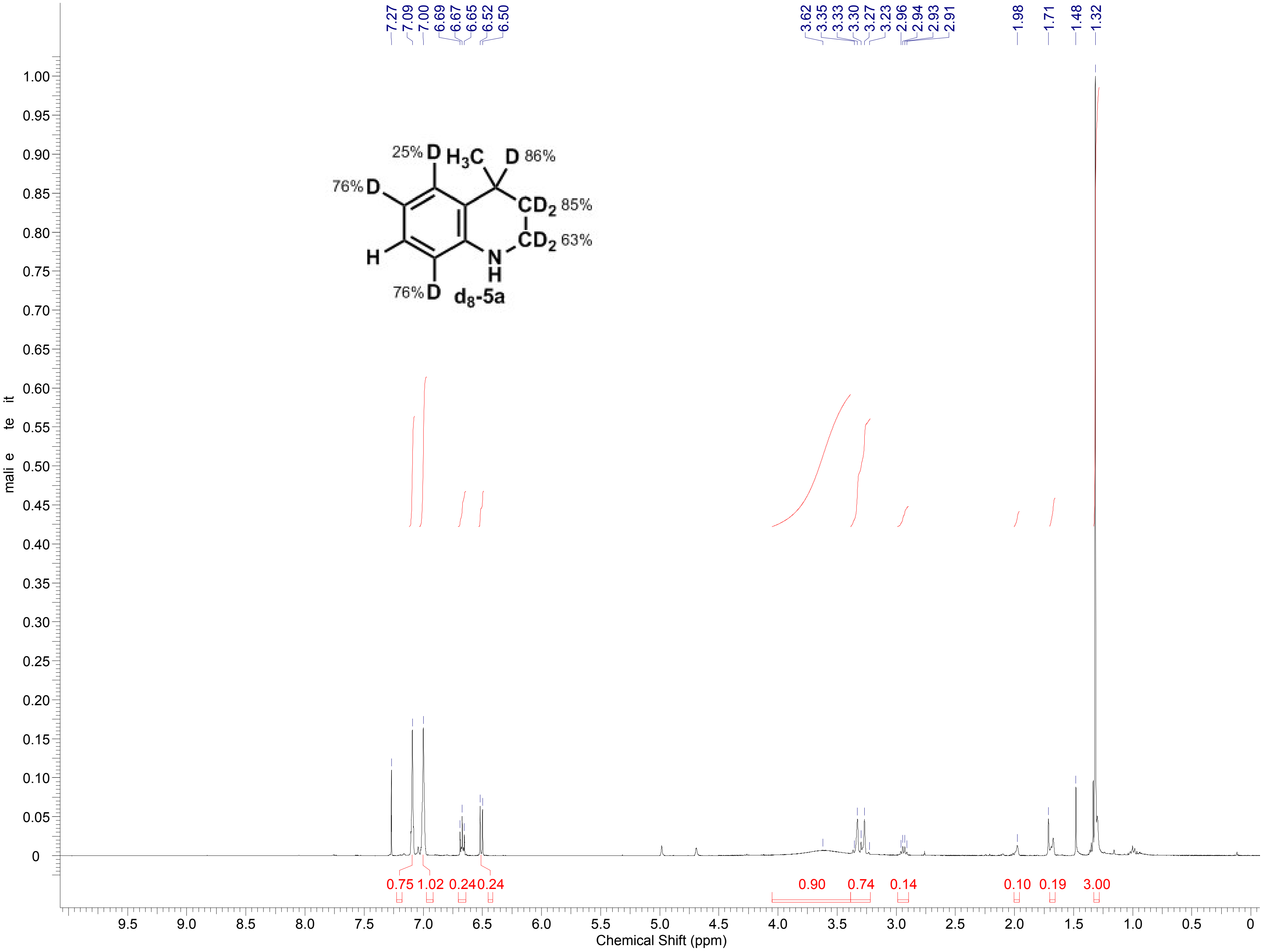
7.27 7.09 7.00 6.69 6.67 6.65 6.52 6.50
3.62 3.35 3.33 3.30 3.27 3.23 2.96 2.94 2.93 2.91
1.98 1.71 1.48 1.32
25% D
76% D
H
76% D
H_3C
D 86%
CD_2 85%
CD_2 63%
N
H
d_8-5a
0.75 1.02 0.24 0.24
0.90 0.74 0.14
0.10 0.19 3.00
1.00 0.95 0.90 0.85 0.80 0.75 0.70 0.65 0.60 0.55 0.50 0.45 0.40 0.35 0.30 0.25 0.20 0.15 0.10 0.05 0
9.5 9.0 8.5 8.0 7.5 7.0 6.5 6.0 5.5 5.0 4.5 4.0 3.5 3.0 2.5 2.0 1.5 1.0 0.5 0
Chemical Shift (ppm)

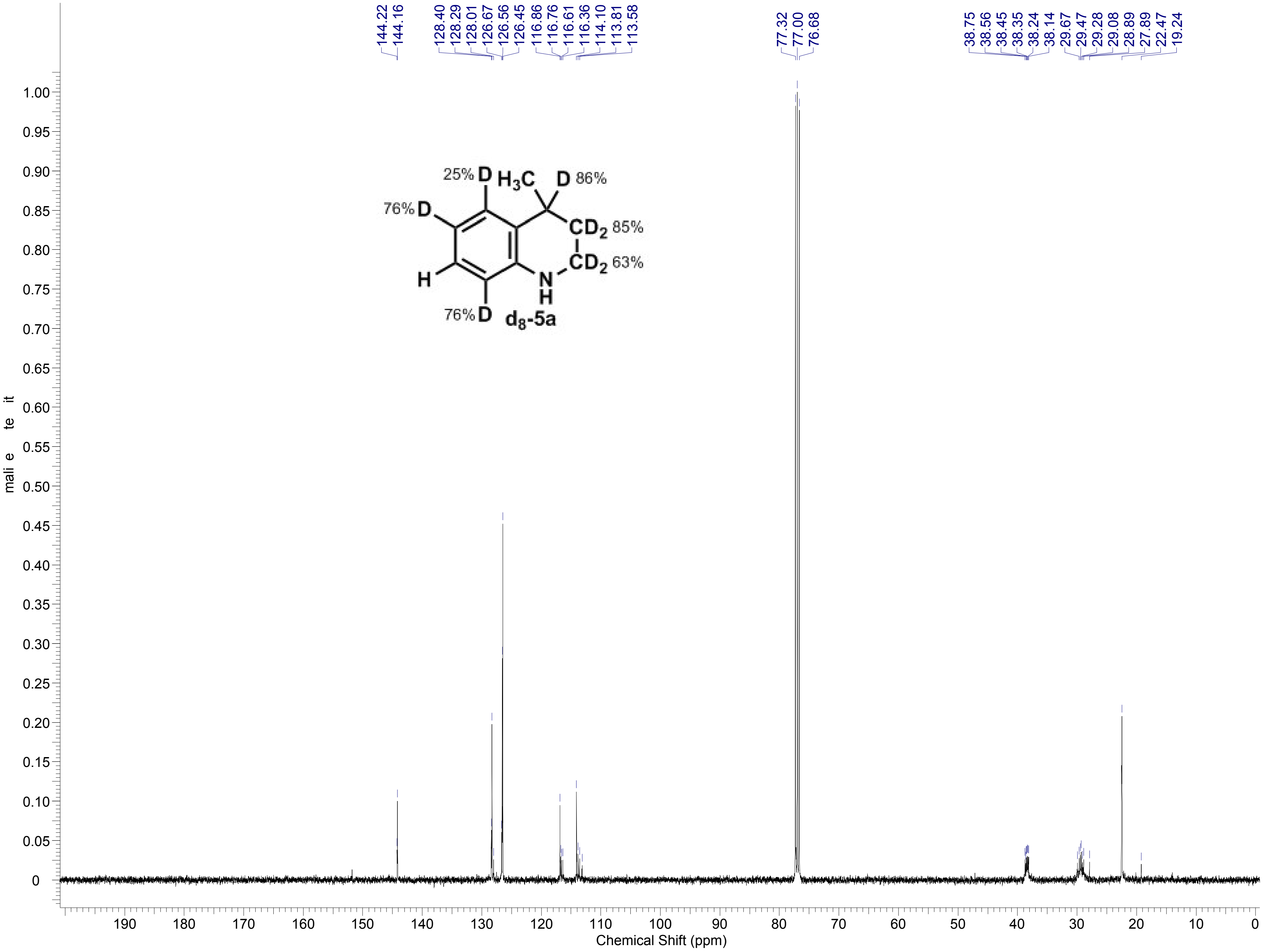
144.22
144.16
128.40
128.29
128.01
126.67
126.56
126.45
116.86
116.76
116.61
116.36
114.10
113.81
113.58
77.32
77.00
76.68
38.75
38.56
38.45
38.35
38.24
38.14
29.67
29.47
29.28
29.08
28.89
27.89
22.47
19.24
25% D
H3C
D 86%
76% D
CD2 85%
CD2 63%
H
N
H
76% D
d8-5a
Chemical Shift (ppm)
